# Supplementary material for: Intronic ATTTC repeat expansions in STARD7 in familial adult myoclonic epilepsy linked to chromosome 2
Source: Nat Commun. 2019 Oct 29;10:4920. doi: 10.1038/s41467-019-12671-y (PMC6820779; doi:10.1038/s41467-019-12671-y)
Supplement: Supplementary file 1 — Supplementary Information [file 41467_2019_12671_MOESM1_ESM.pdf]

Supplementary Information

Intronic ATTTC repeat expansions in *STARD7* in familial adult myoclonic epilepsy linked to chromosome 2

Corbett, *et al.*

Supplementary Figures

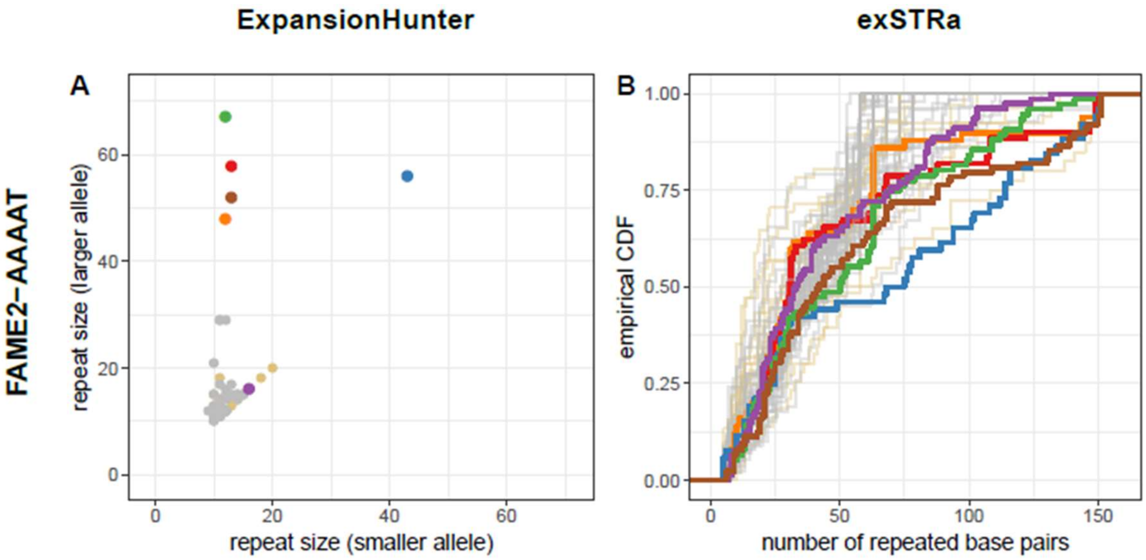

Supplementary Figure. 1. AAAAT repeat sizes predicted by ExpansionHunter and exSTRA.

Estimated sizes of the AAAAT repeats in two affected individuals from Family 1 (red, orange), one from Family 3 (brown) and three affected individuals from Family 19 (blue, green, purple), compared to 69 individuals without FAME using TruSeq Nano (grey) or KAPA Hyper (tan) library preparation. Panels A displays estimated repeat size by Expansion Hunter while panel B shows the empirical cumulative distribution functions from exSTRa. Note the high variability of allele sizes of the endogenous AAAAT repeat. Data underlying this figure is provided in the Source Data file.

Supplementary Figure 2. Pedigrees of 22 families typically affected by FAME (Fig. S2a-2v follow on multiple pages). Pedigrees for 22 tested families with FAME as per Table 1 in the main text. Keys on each page apply to all pedigrees on that page. Chromosome 2 markers are shown in mapped families where available.

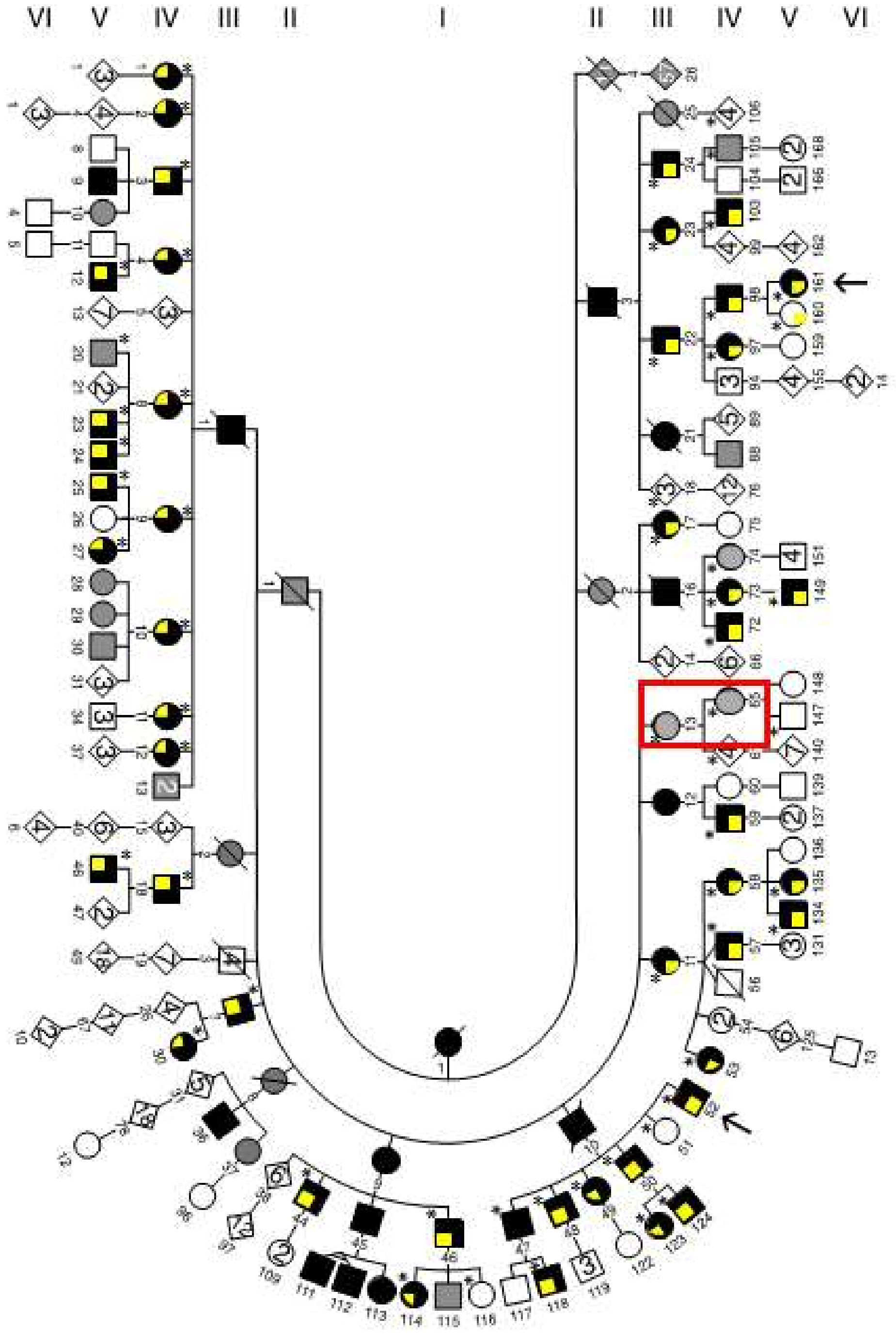

a. Family 1

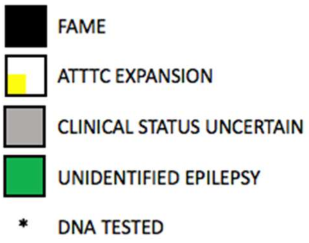

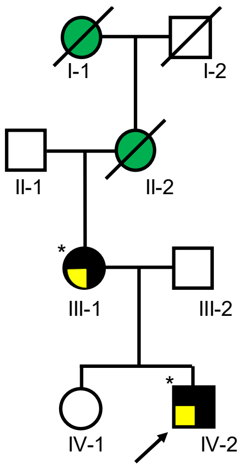

b. Family 2

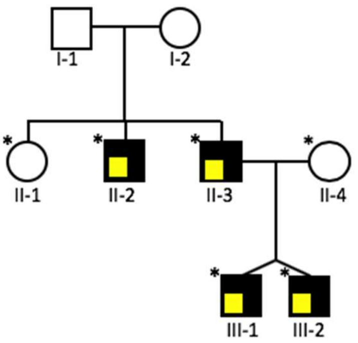

c. Family 3

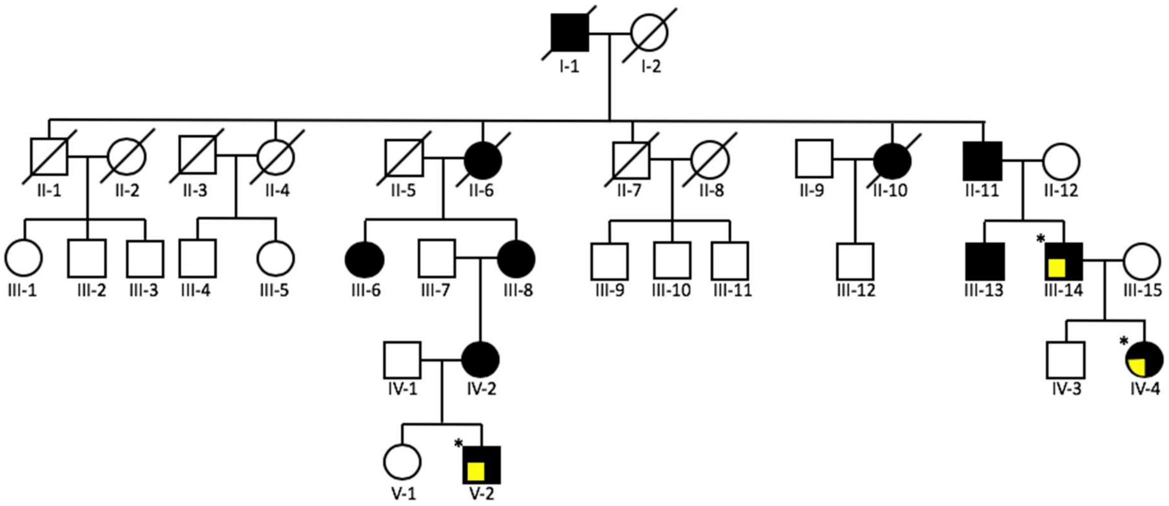

d. Family 4

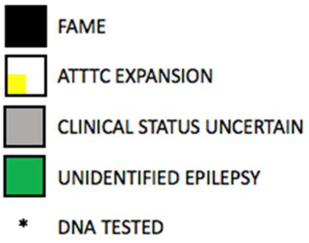

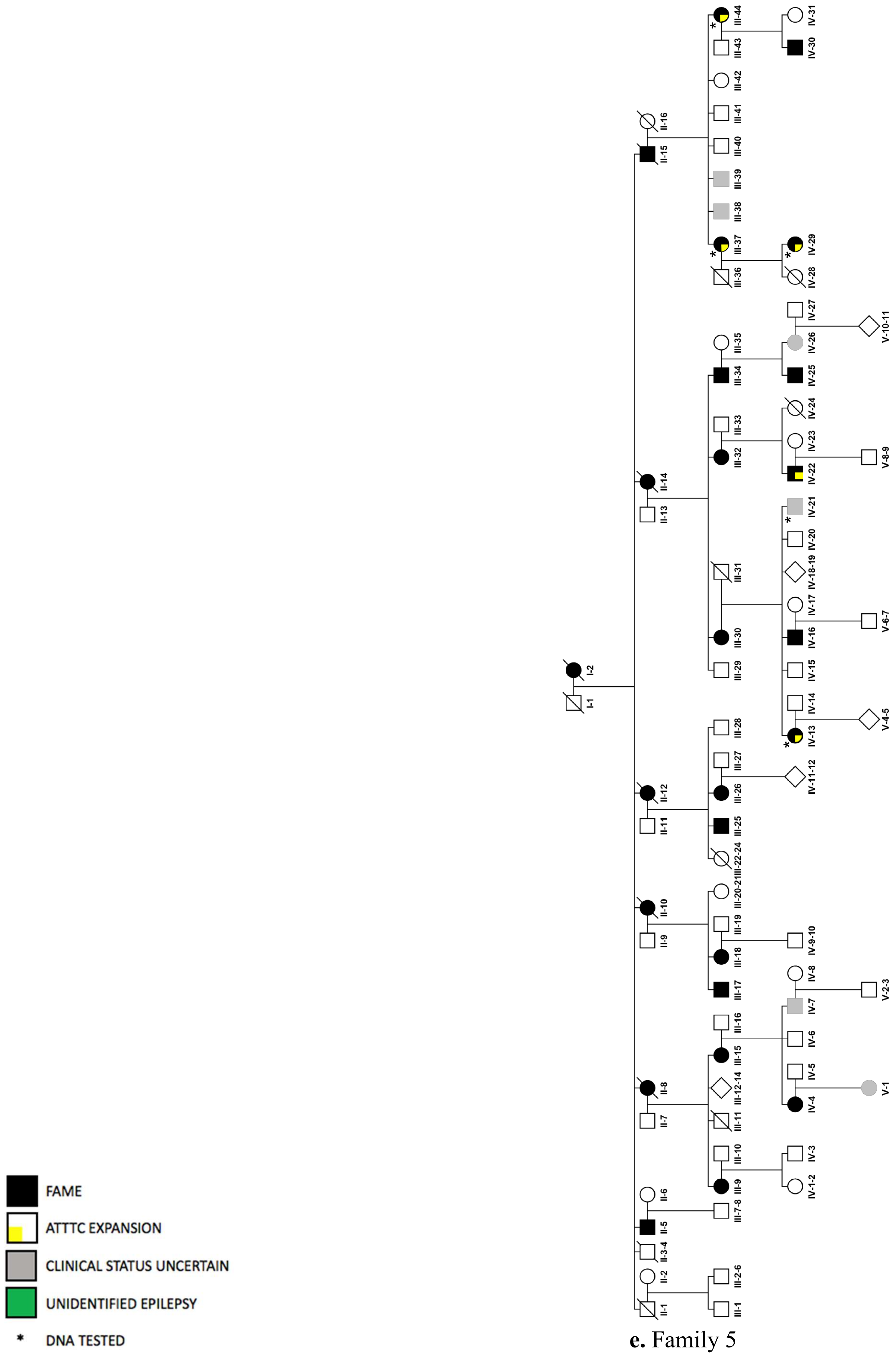
FAME
ATTTC EXPANSION
CLINICAL STATUS UNCERTAIN
UNIDENTIFIED EPILEPSY
* DNA TESTED

e. Family 5

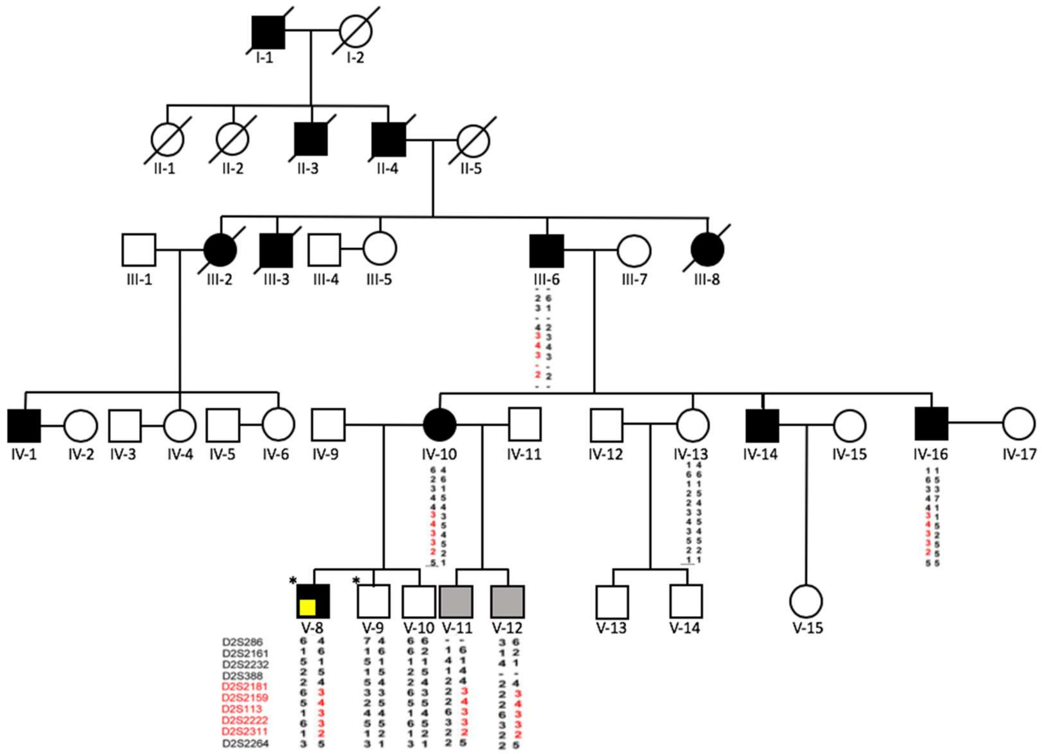

f. Family 6

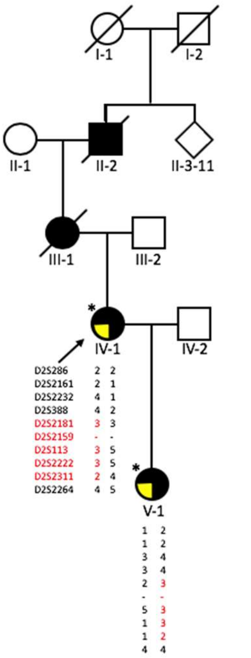

g. Family 7

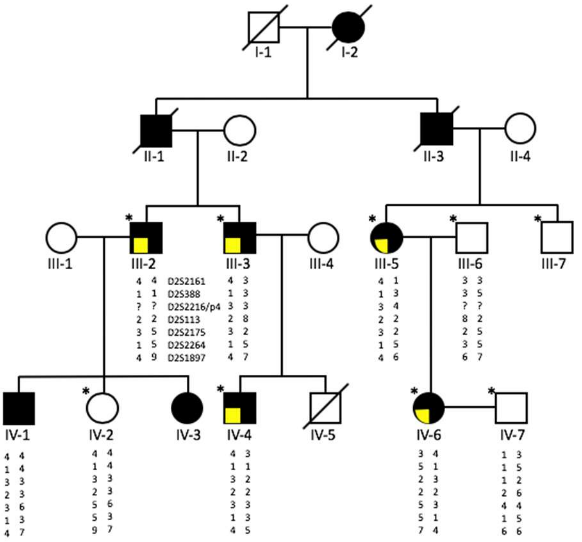

h. Family 8

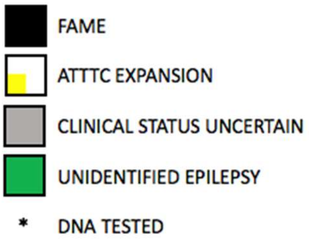

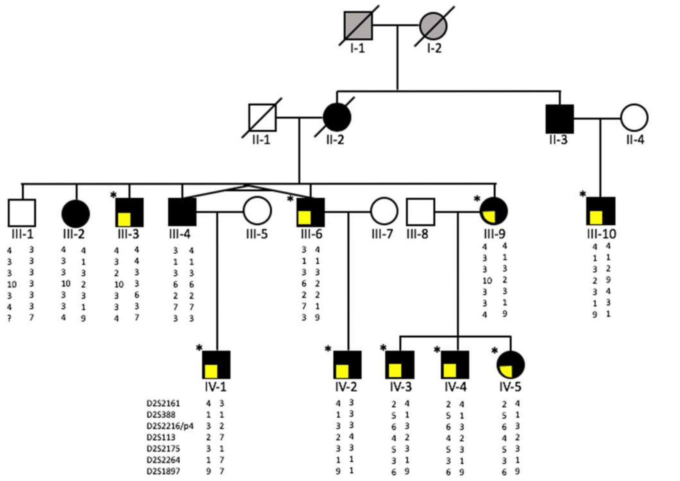

i. Family 9

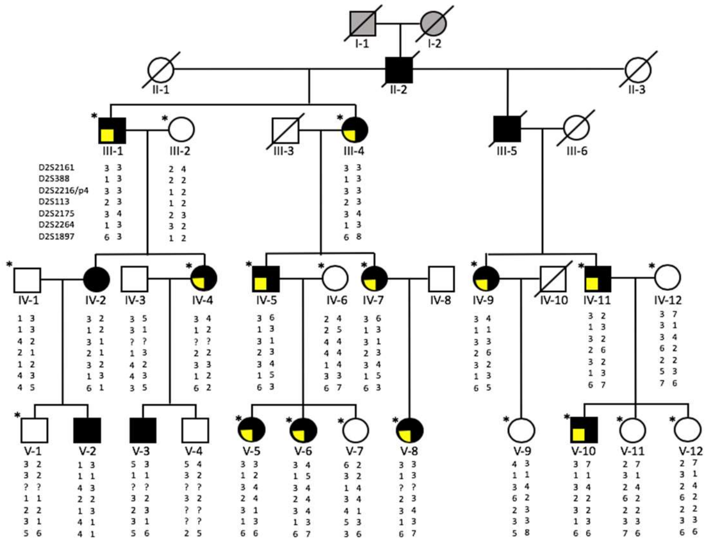

j. Family 10

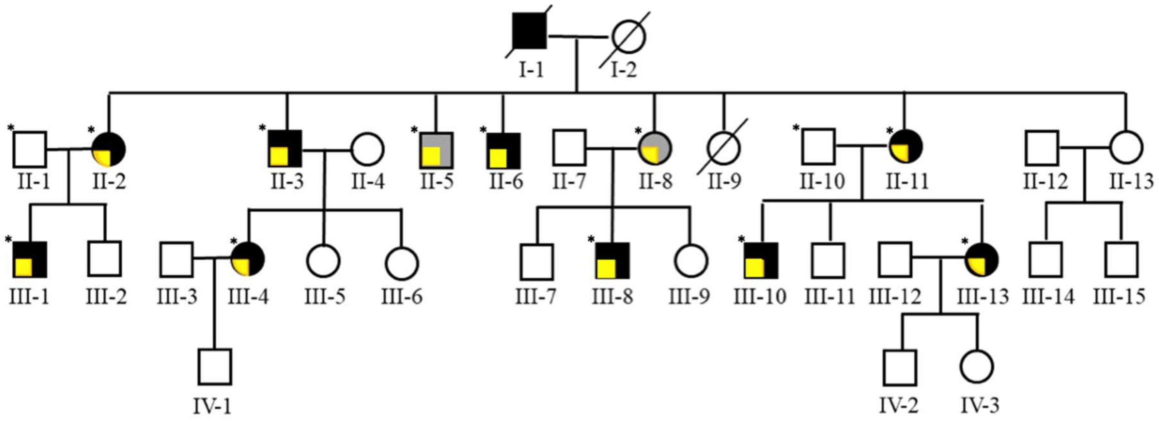

k. Family 11

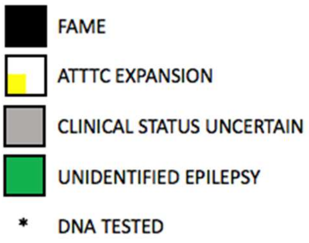

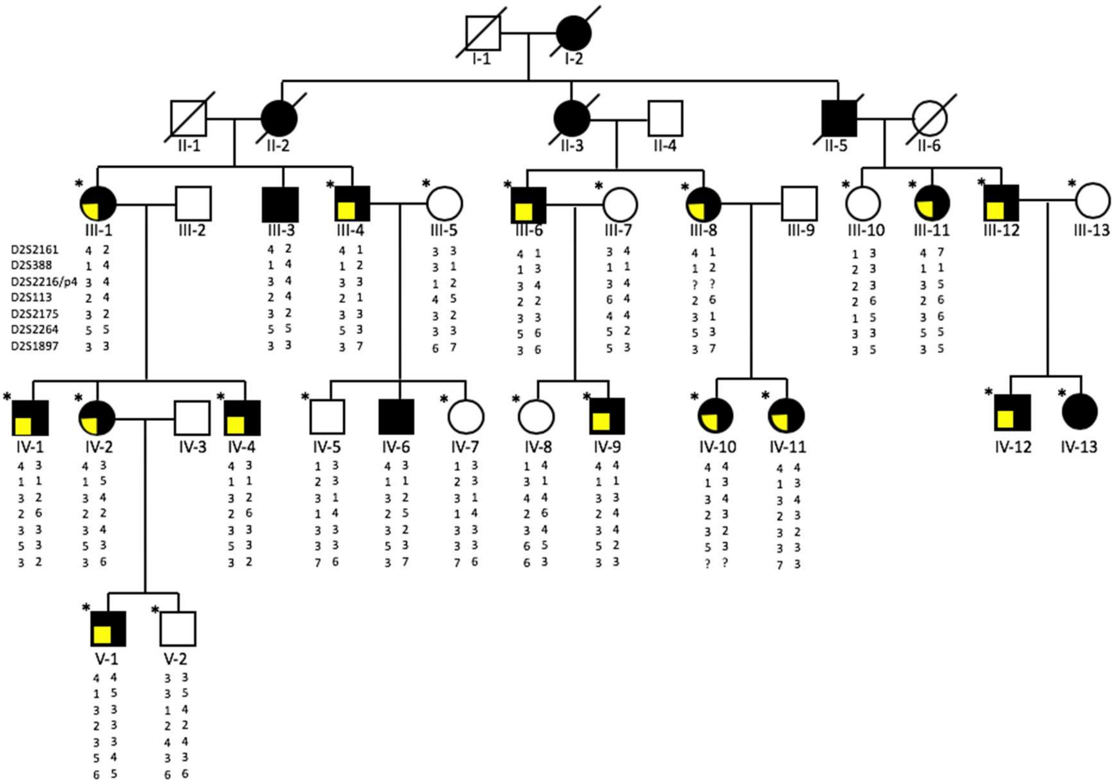

l. Family 12

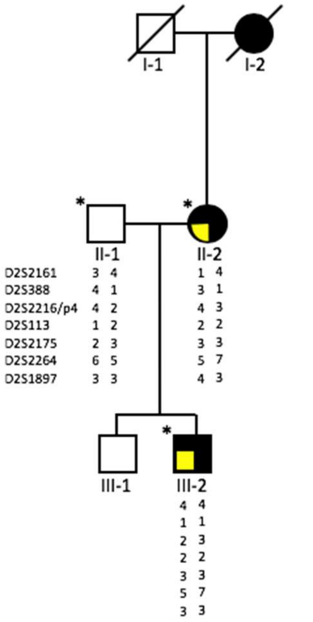

m. Family 13

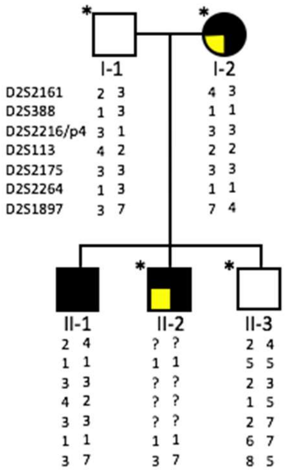

n. Family 14

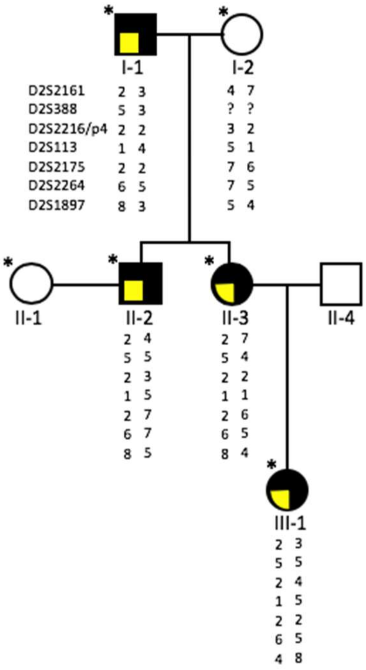

o. Family 15

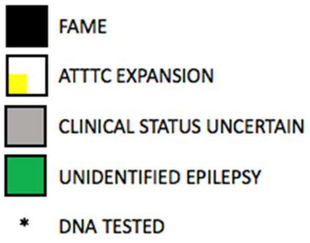

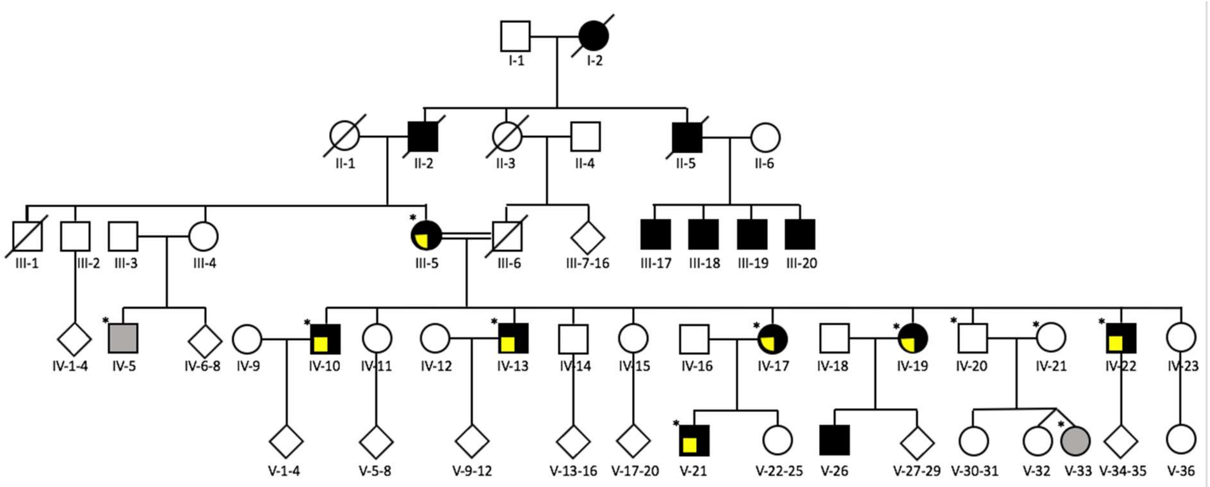

p. Family 16

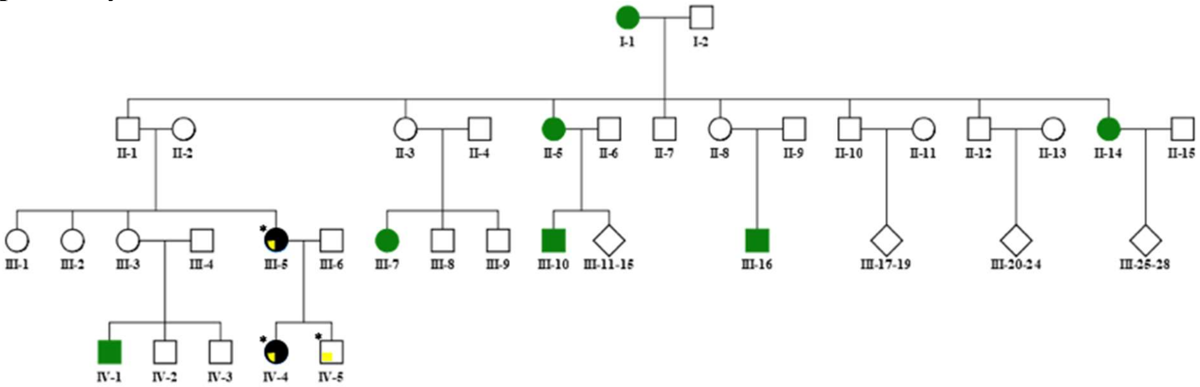

q. Family 17

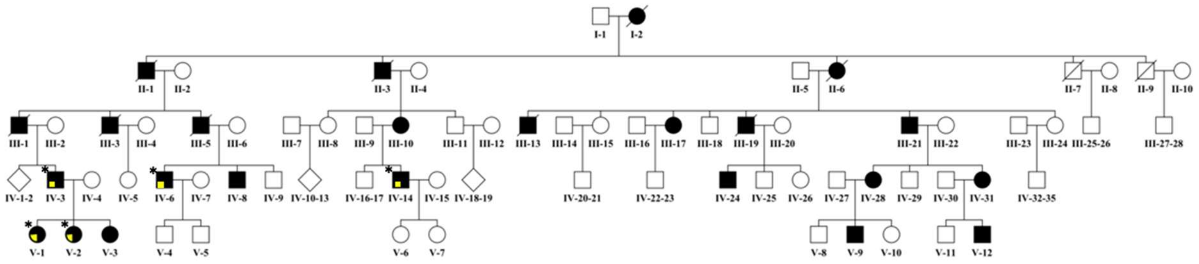

r. Family 18

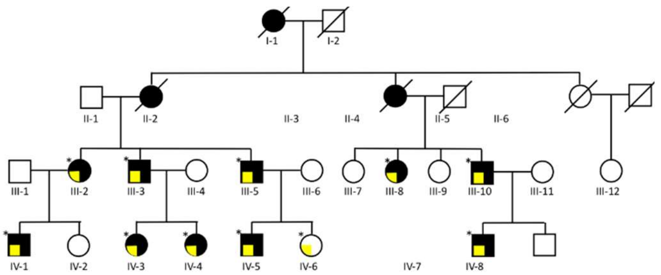

s. Family 19

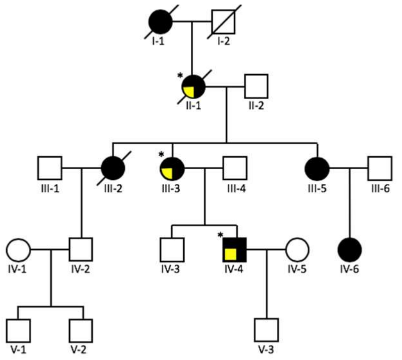

t. Family 20

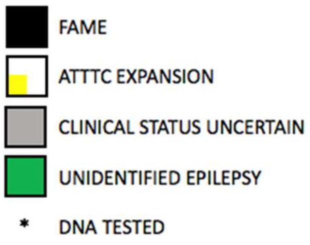

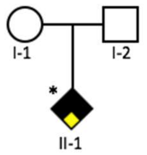

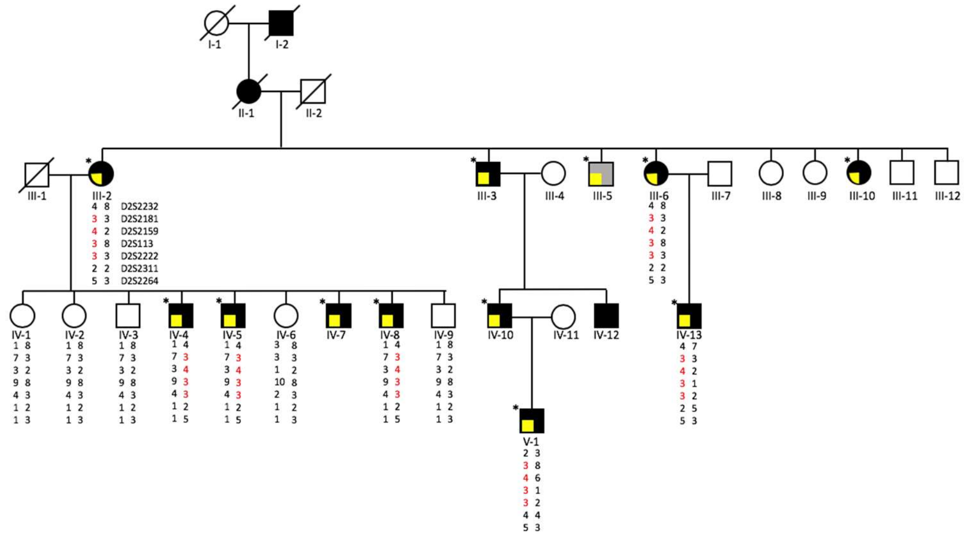

u. Family 21 **v**. Family 22

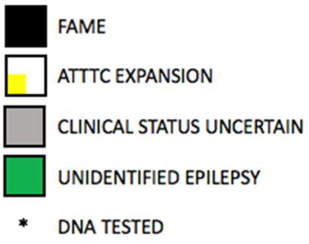

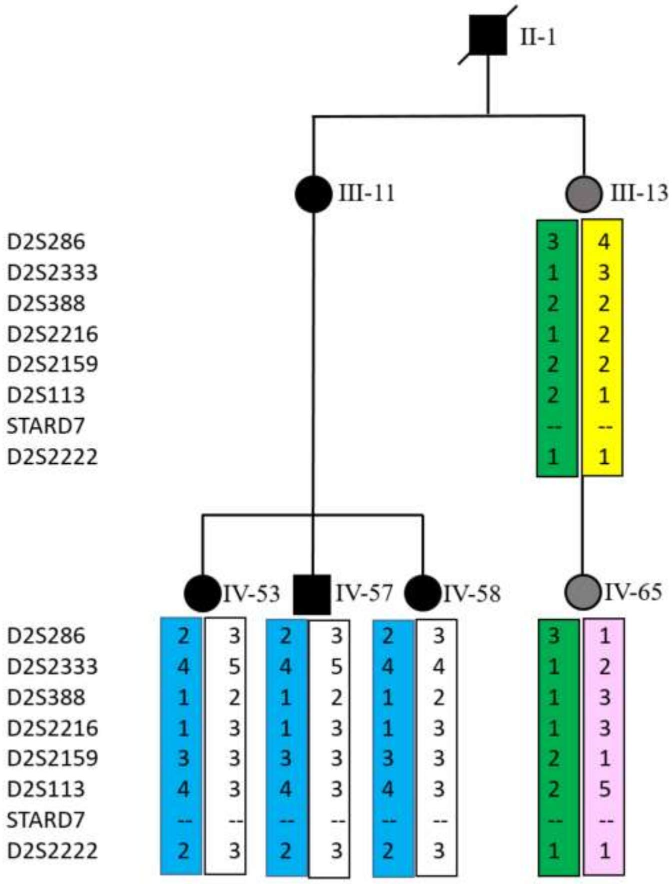

Supplemetary Figure 3. Family 1 microsattelite markers
Microsatellite markers for selected members of Family 1 show family members III-13 and IV-65 do not share the same haplotypes as IV-53, IV-57 and IV-58 with confirmed diagnosis and positive for the STARD7 ATTTC expansion.

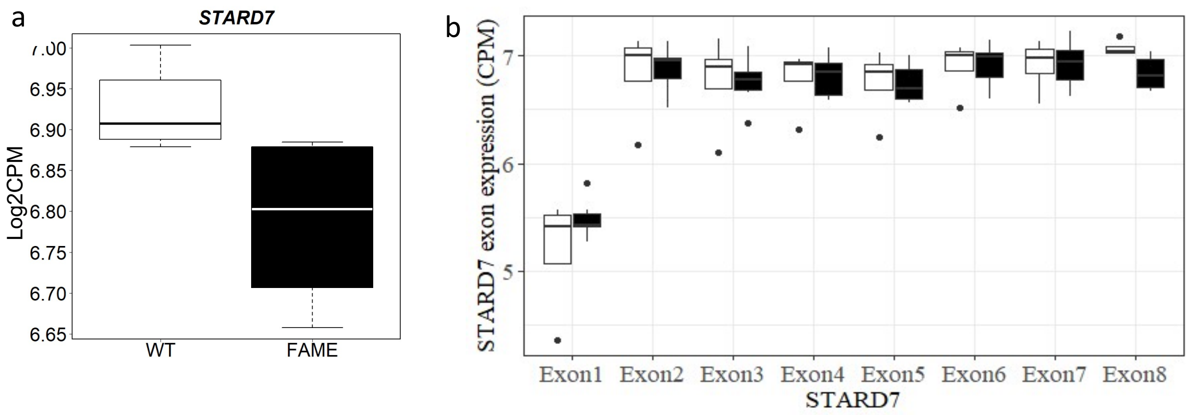

Supplementary Figure 4. RNA-Seq Expression of STARD7

a. Boxplot of STARD7 gene expression in fibroblasts represented by log2 transformed CPM (read counts per million mapped reads) values. Data represent control fibroblast (white box; n=4) and FAME fibroblasts (black box; n=6). The difference between groups is not significant (log base 2-fold change 0.0432, p=0.838 FDR=1). **b**. Log2 CPM values of each of the eight exons of *STARD7* transcript ENST00000337288.9. Data underlying this figure is provided in the Source Data file.

Supplementary Tables

Supplementary Table 1. Additional phenotypes and mapping information

Family	Nationality	Total affected	ID	Dementia	DRE	Ataxia	Evidence for chr2 linkage	Other	References
1	Australian/New Zealand of European ancestry	55	0/55	1/55 (88y; unknown onset)	0/55	0/55	Chr2 linkage with maximum LOD=4.5 [a]	Migraine: 18/55	Crompton *et al.* 2012 [1]
2	Italian	2	0/2	0/2	0/2	0/2	No linkage to chr2	Psychiatric symptoms 1/2	
3	Italian	4	Mild to moderate 1/4; borderline 1/4	0/4	4/4	0/4	Chr2 suggestive linkage with maximum LOD = 1.12	Prominent visuospatial impairment in 4/4 Psychiatric symptoms (reoccurring thoughts and compulsive behaviour) 1/4	Suppa *et al.* 2009[2]
4	Italian	11	Mild 3/11; moderate 1/11	0/11	2/11	0/11	*ADRA2B*; c.675_686delTGGTGGGGCTTTinsGTTTGGCAG; p.H225_L229delinsQ225_F_G_R228 Chr2 linkage with maximum LOD = 3.46		Guerrini *et al.* 2001, de Fusco *et al.* 2013[3,4]
5	Italian	25	1/16	1/16 (58y)	0/16	1/16	Chr2 linkage with maximum LOD = 3.5	Migraine/headache: 4/16; Psychiatric disorders (depression and / or anxiety with panic attacks) 6/16	Licchetta *et al.* 2013 [5]
6	Italian	12 (3 studied)	0/3	0/3	1/3	2/3	Linkage to chr2 and chr8 previously excluded however removal of uncertain cases (V-9 to V-12) shows segregation of markers to chr2 and this family shares the Southern Italian FAME2 haplotype of	Migraine/headache: 2/3; Psychiatric disorders 1/3	Gardella *et al.* 2006 [6]. Family 3 in Henden *et al.* 2015 [7]

							known linked families (Figure S2f).		
7	Italian	4	0/4	Not known	0/4	Not known	This family segregates the Southern Italian FAME2 haplotype of known linked families (Figure S2g).	Psychiatric disorders 1/4	
8	Italian	10 (6 studied)	0/6	1/6 (75y; onset after 70 y)	0/6	0/6	Chr2 suggestive linkage with maximum LOD = 2.1		Madia *et al.* 2008 [8]
9	Italian	13 (11 studied)	0/11	0/11	0/11	0/11	Chr2 implicated by cumulative linkage with family 10 maximum LOD = 3.32 (this family LOD = 2.42)		de Falco *et al.* 2003[9]
10	Italian	16 (14 studied)	0/14	1/14 (82y; onset after 70 y)	0/14	0/14	Chr2 implicated by cumulative linkage with family 9 maximum LOD = 3.32 (this family LOD = 0.9)		de Falco *et al.* 2003 [9]
11	Italian	10 (5 studied)	0/5	1/5 (79y; onset after 70 y)	0/5	0/5	No evidence for or against linkage to chr2		Coppola *et al.* 2016 [10]
12	Italian	21 (17 studied)	0/5	0/17	1/17	0/17	Chr2 linkage with maximum LOD = 5.9		Striano *et al.* 2005 [11]
13	Italian	3	0/3	0/3	0/3	0/3	Chr2 markers suggest affected individuals have the known Southern Italian FAME2 haplotype		Coppola *et al.* 2016 [10]; Striano *et al.* 2004 [12]
14	Italian	3	0/3	0/3	0/3	0/3	Chr2 suggestive linkage with maximum LOD = 1.5		Coppola *et al.* 2011 [13]; Striano *et al.* 2004 [12]
15	Italian	4	0/4	0/4	2/4	0/4	*ADRA2B*; c.675_686delTGGTGGGGCTTTinsGTTTGGCAG; p.H225_L229delinsQ225_F_G_R228 Chr2 markers suggest affected individuals have the known Northern Italian FAME2 haplotype		de Fusco *et al.* 2013 [4]

16	Iraqi of Sephardic Jewish ancestry	15 (10 studied)	0/10	0/8 (2 deceased: not known)	1/10	0/10	Chr2 suggestive linkage with maximum LOD = 1.8		
17	Israeli of Sephardic Jewish ancestry	2	0/2	0/2	0/2	0/2	No evidence for or against linkage to chr2		
18	South African of European ancestry	24 (15 studied)	0/15	0/15	0/15	0/15	No evidence for or against linkage to chr2	Lower limb myoclonus 1/15 Genetics:	van Coller *et al.* 2017 [14]
19	French/ Spanish	13	0/10	0/10	0/10	0/10	Chr2 linkage with maximum LOD = 3.3		Saint-Martin *et al.* 2008; Labauge *et al.* 2002 [15,16]
20	French	7 (2 studied)	Not known	Not known	Not known	Not known	Chr2 markers in three affected individuals do not exclude the known FAME2 interval	Migraine: 1/2	Henden *et al.* 2016 [7]
21	Syrian	1	Not known	Not known	Not known	0/1	No evidence for or against linkage to chr2		
22	Italian	11 (10 studied [b])	2/10	0/10	1/10	0/10	Chr2 suggestive linkage with maximum LOD = 1.8	Psychiatric symptoms 2/10, Epileptic spasms 2/10	

[a] All maximum LOD scores are reported with recombination fraction θ=0.
[b] One family member last evaluated at 9 years of age.
Abbreviations: DRE: Drug resistant epilepsy; ID: Intellectuatl disability; LOD: Logarithm of the odds; y: years; n: number of individuals.

Supplementary Table 2. exSTRa database

exSTRa FAME2 loci (requires: exSTRa >=0.8)

locus	long_name	gene	motif	strand	chrom	hg19_start	hg19_end	perIndel	perMatch	location
FAME2-AAAAT	FAME2-AAAAT	STARD7	AAAAT	+	chr2	96862805	96862859	0	100	intron
FAME2-AAATG	FAME2-AAATG	STARD7	AAATG	+	chr2	96862825	96862826	0	100	intron

Supplementary Table 3. Primer Sequences

**Primer**	**Sequence 5'-3'**
STARD7F	GGCTACTTACGTGCCAGATAAC
STARD7R	TGCCCAGCTACACTGTCTCTT
RP-PCR-FAME2-P1	**[FAM]**-GGCTACTTACGTGCCAGATAAC
RP-PCR-FAME2-P2/ RP-PCR STARD7R	**[FAM]**-TGCCCAGCTACACTGTCTCTT
RP-PCR-FAME2-P4.5	TACGCATCCCAGTTTGAGACGAAAATAAA ATAAAATAAAATAAAATAAAAT
RP-PCR-FAME2-P4.6	TACGCATCCCAGTTTGAGACGAAATGAAA TGAAATGAAATGAAATGAAATG
RP-PCR-FAME2-P4.7/ RP-PCR-STARD7-P4.T	TACGCATCCCAGTTTGAGACGTTTTATTTT ATTTTATTTTATTTTATTTTA
RP-PCR-FAME2-P4.8/ RP-PCR-STARD7-P4.C	TACGCATCCCAGTTTGAGACGTTTCATTTC ATTTCATTTCATTTCATTTCA
RP-PCR-P3/ RP-PCR-STARD7-P3	TACGCATCCCAGTTTGAGACG
HPRT_F1	TGACACTGGCAAAACAATGCA
HPRT_R1	GGTCCTTTTCACCAGCAAGCT
STARD7Ex1F1	GCCATGCCTCTGCCTTGATG
STARD7Ex2R1	TTCTTCCAACCGCTTCATCTC
STARD7Ex3F (primer bank accession: 151301034c3) [17]	GTGACACCTCGGCAGTTCTTC
STARD7Ex4R (primer bank accession: 151301034c3) [17]	ACCTCGGAACCACTAACCACA

Supplementary References

1. Crompton, D. E. *et al.* Familial adult myoclonic epilepsy: recognition of mild phenotypes and refinement of the 2q locus. *Arch. Neurol.* **69**, 474–481 (2012).
2. Suppa, A. *et al.* Clinical, neuropsychological, neurophysiologic, and genetic features of a new Italian pedigree with familial cortical myoclonic tremor with epilepsy. *Epilepsia* **50**, 1284–1288 (2009).
3. Guerrini, R. *et al.* Autosomal dominant cortical myoclonus and epilepsy (ADCME) with complex partial and generalized seizures: A newly recognized epilepsy syndrome with linkage to chromosome 2p11.1-q12.2. *Brain J. Neurol.* **124**, 2459–2475 (2001).
4. De Fusco, M. *et al.* The α2B -adrenergic receptor is mutant in cortical myoclonus and epilepsy. *Ann. Neurol.* (2013). doi:10.1002/ana.24028
5. Licchetta, L. *et al.* A novel pedigree with familial cortical myoclonic tremor and epilepsy (FCMTE): Clinical characterization, refinement of the FCMTE2 locus, and confirmation of a founder haplotype. *Epilepsia* **54**, 1298–1306 (2013).
6. Gardella, E. *et al.* Autosomal Dominant Early-onset Cortical Myoclonus, Photic-induced Myoclonus, and Epilepsy in a Large Pedigree. *Epilepsia* **47**, 1643–1649 (2006).
7. Henden, L. *et al.* Identity by descent fine mapping of familial adult myoclonus epilepsy (FAME) to 2p11.2-2q11.2. *Hum. Genet.* (2016). doi:10.1007/s00439-016-1700-8
8. Madia, F. *et al.* Benign adult familial myoclonic epilepsy (BAFME): evidence of an extended founder haplotype on chromosome 2p11.1-q12.2 in five Italian families. *Neurogenetics* **9**, 139–142 (2008).
9. de Falco, F. A. *et al.* Benign adult familial myoclonic epilepsy: genetic heterogeneity and allelism with ADCME. *Neurology* **60**, 1381–1385 (2003).

10. Coppola, A. *et al.* Psychiatric comorbidities in patients from seven families with autosomal dominant cortical tremor, myoclonus, and epilepsy. *Epilepsy Behav. EB* **56**, 38–43 (2016).

11. Striano, P., Madia, F., Minetti, C., Striano, S. & Zara, F. Electroclinical and genetic findings in a family with cortical tremor, myoclonus, and epilepsy. *Epilepsia* **46**, 1993–1995 (2005).

12. Striano, P. *et al.* A New Benign Adult Familial Myoclonic Epilepsy (BAFME) Pedigree Suggesting Linkage to Chromosome 2p11.1-q12.2. *Epilepsia* **45**, 190–192 (2004).

13. Coppola, A. *et al.* Natural history and long-term evolution in families with autosomal dominant cortical tremor, myoclonus, and epilepsy. *Epilepsia* **52**, 1245–1250 (2011).

14. van Coller, R., van Rootselaar, A.-F., Schutte, C. & van der Meyden, C. H. Familial cortical myoclonic tremor and epilepsy: Description of a new South African pedigree with 30 year follow up. *Parkinsonism Relat. Disord.* **38**, 35–40 (2017).

15. Saint-Martin, C. *et al.* Refinement of the 2p11.1-q12.2 locus responsible for cortical tremor associated with epilepsy and exclusion of candidate genes. *Neurogenetics* **9**, 69–71 (2008).

16. Labauge, P. *et al.* Absence of linkage to 8q24 in a European family with familial adult myoclonic epilepsy (FAME). *Neurology* **58**, 941–944 (2002).

17. Spandidos, A., Wang, X., Wang, H. & Seed, B. PrimerBank: a resource of human and mouse PCR primer pairs for gene expression detection and quantification. *Nucleic Acids Res.* **38**, D792–D799 (2009).
