# Supplementary material for: Intronic ATTTC repeat expansions in STARD7 in familial adult myoclonic epilepsy linked to chromosome 2
Source: Nat Commun. 2019 Oct 29;10:4920. doi: 10.1038/s41467-019-12671-y (PMC6820779; doi:10.1038/s41467-019-12671-y)
Supplement: Supplementary file 2 — Supplementary Data 1 [file 41467_2019_12671_MOESM2_ESM.pdf]

Supplementary Data 1. Combined summary of RP-PCR results from all families

A=Affected, N=Not affected, U=Uncertain clinical diagnosis.

Family	DNA number	Disease Status	Pedigree Number	TTTCA RP-PCR trace
N/A	Water	Control	N/A	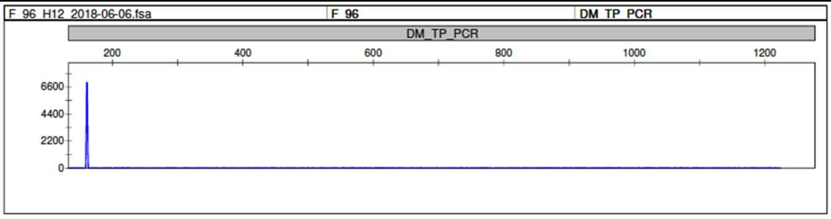
N/A	Blood bank	Control	N/A	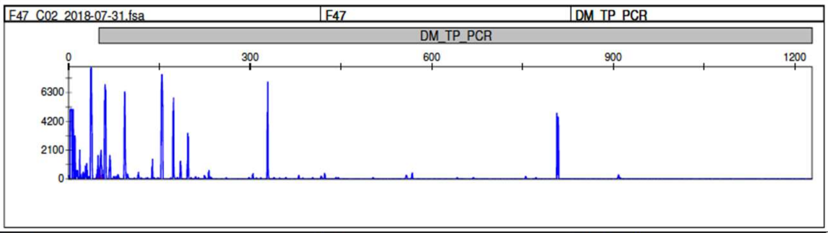
1	T19334	A	III-7	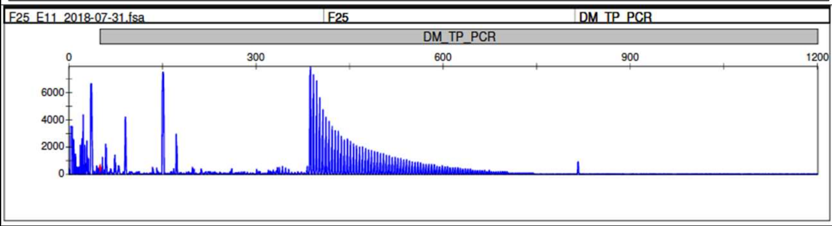
1	T19327	A	III-11	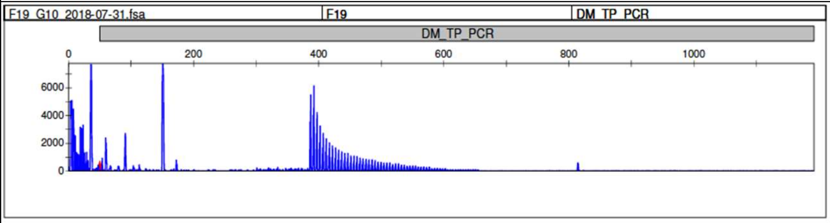
1	T19337	A	III-13	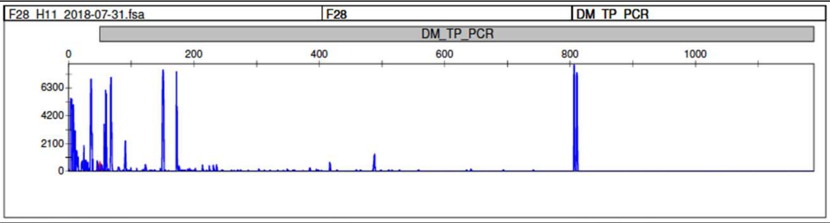
1	T20147	A	III-17	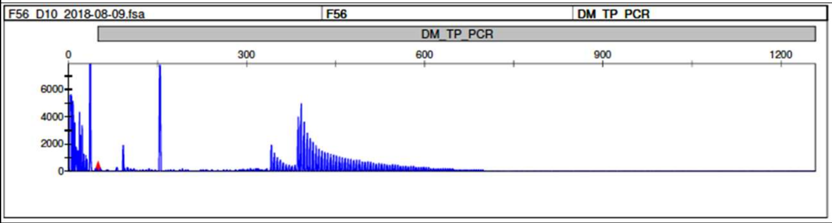
1	T19298	N	III-18	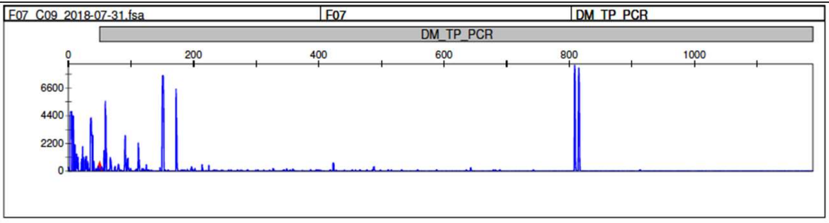

Family	DNA number	Disease Status	Pedigree Number	TTTCA RP-PCR trace
1	T19293	A	III-22	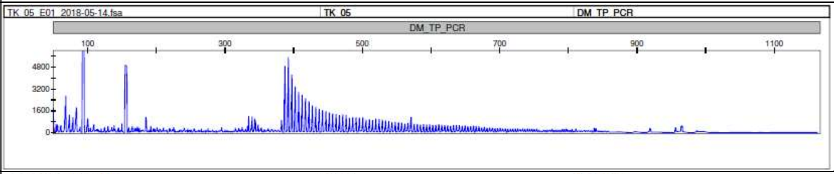
1	T19297	A	III-23	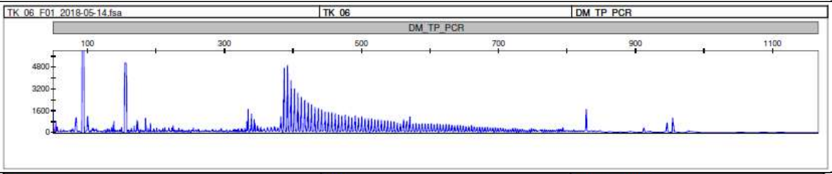
1	T19011	A	III-24	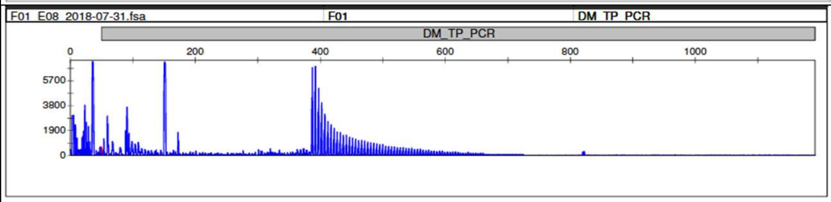
1	T20146	A	IV-1	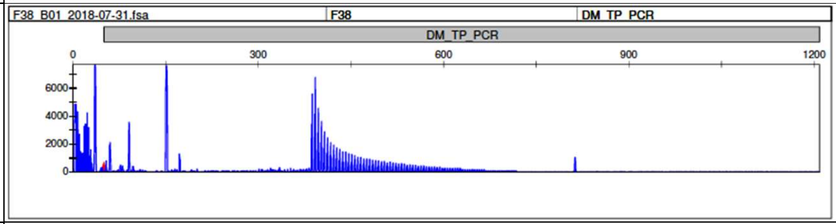
1	T19328	A	IV-2	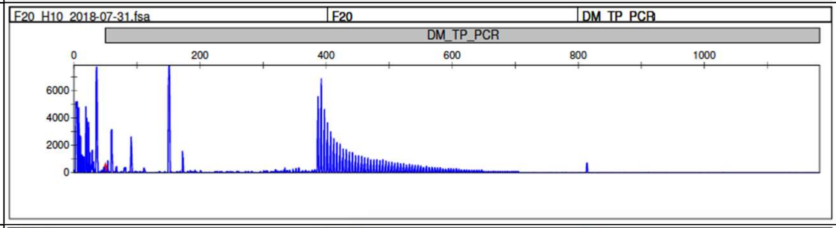
1	T20298	A	IV-3	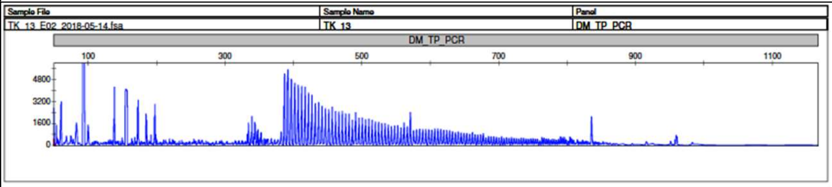
1	T19329	A	IV-4	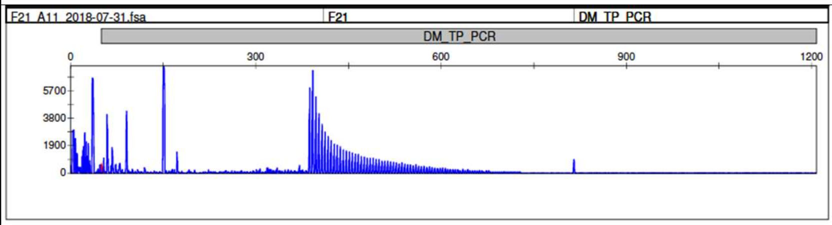
1	T19324	A	IV-8	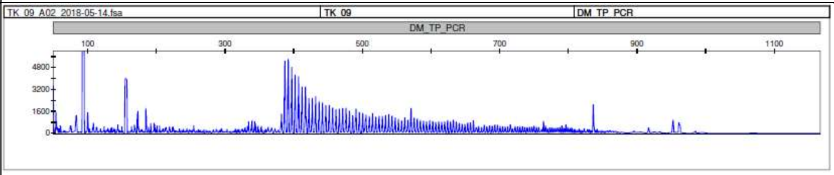
1	T19323	A	IV-9	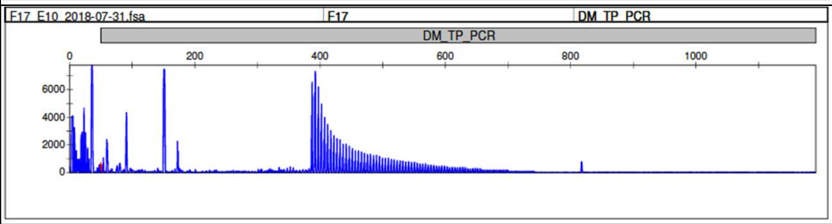

Family	DNA number	Disease Status	Pedigree Number	TTTCA RP-PCR trace
1	T19304	A	IV-10	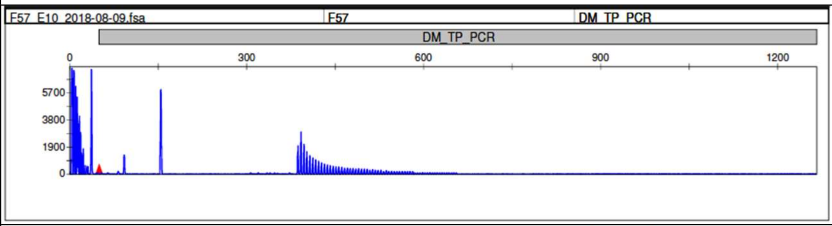
1	T19325	A	IV-11	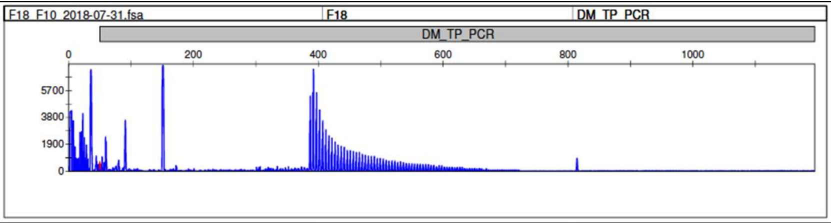
1	T19305	A	IV-12	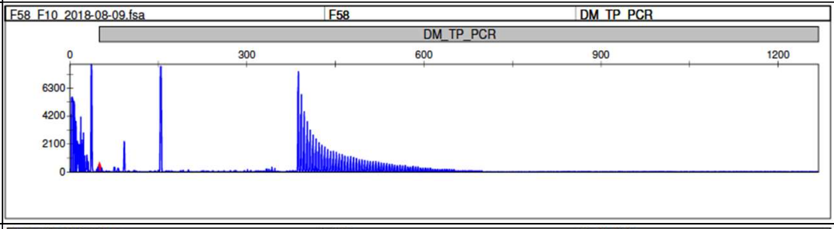
1	T19318	A	IV-18	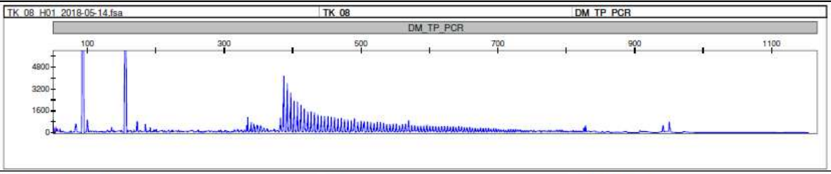
1	T19320	A	IV-30	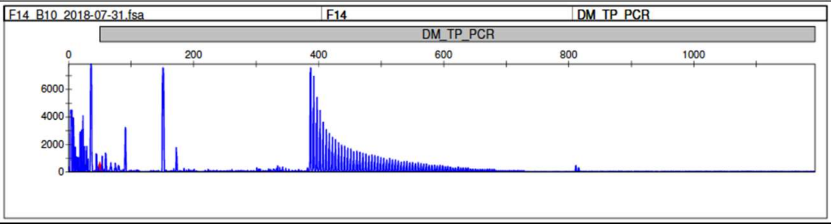
1	T20016	A	IV-44	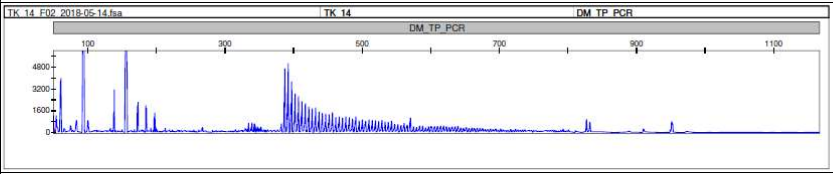
1	T20197	A	IV-46	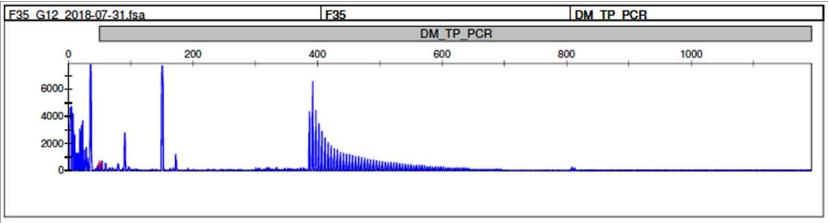
1	T19340	A	IV-47	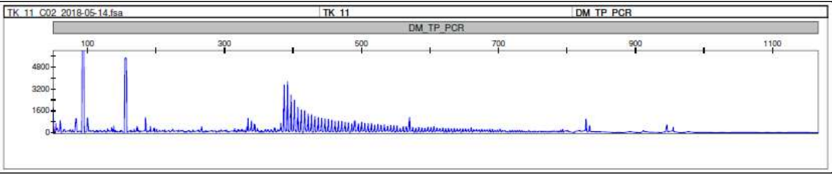

Family	DNA number	Disease Status	Pedigree Number	TTTCA RP-PCR trace
1	T19225	A	IV-48	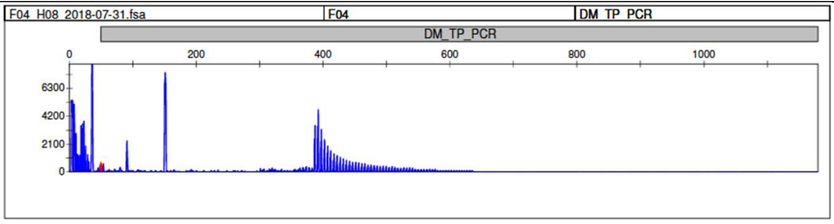
1	T19299	A	IV-49	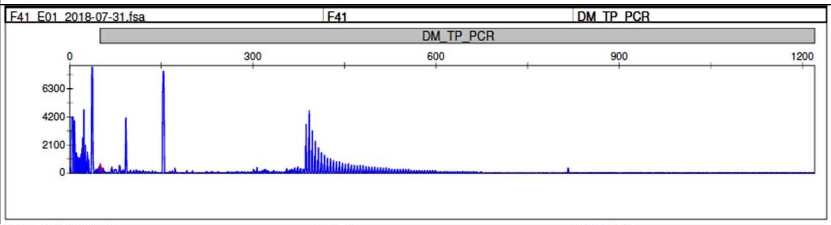
1	39290	A	IV-50	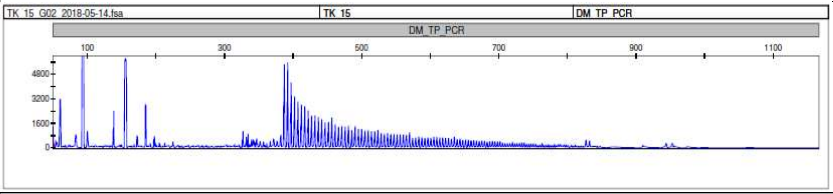
1	T19314	N	IV-51	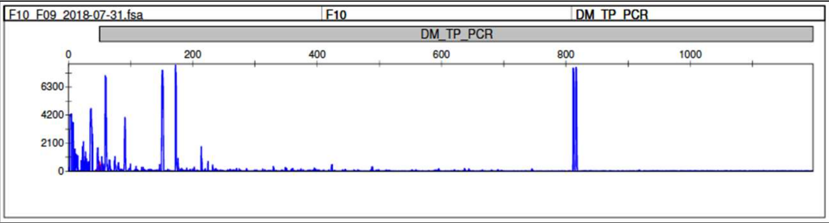
1	T19315	A	IV-52	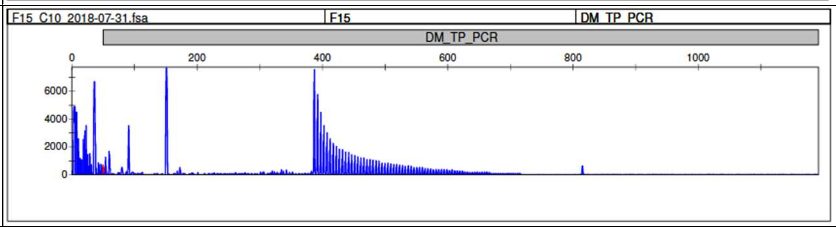
1	T19302	A	IV-53	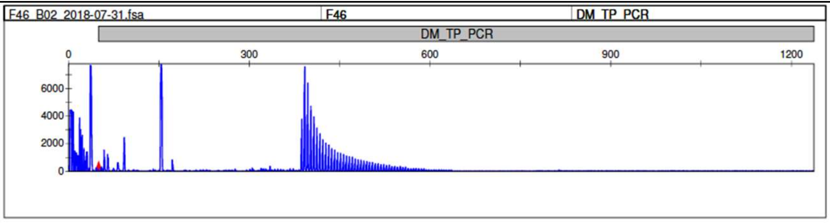
1	T19331	A	IV-57	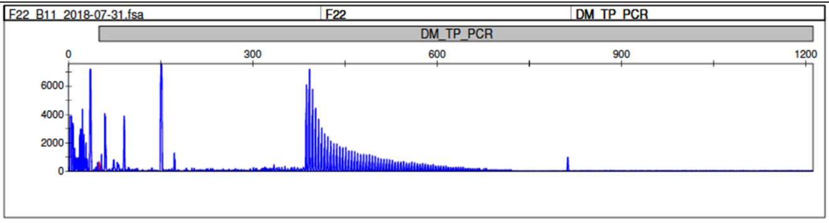
1	T19303	A	IV-58	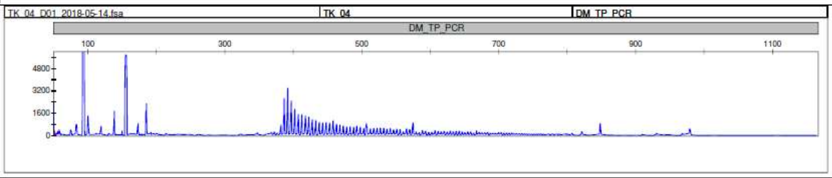

Family	DNA number	Disease Status	Pedigree Number	TTTCA RP-PCR trace
1	T19316	A	IV-59	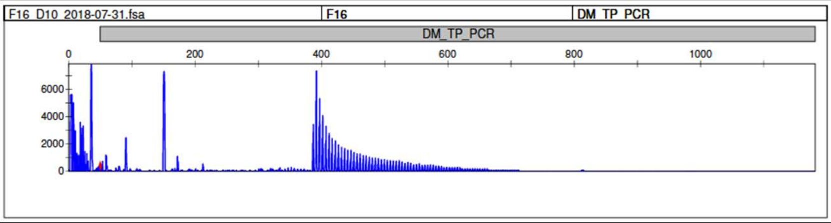
1	T19336	N	IV-61	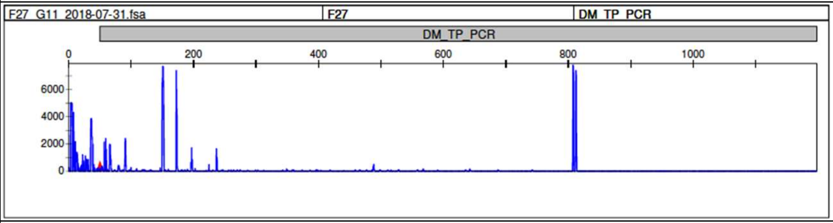
1	T19335	N	IV-62	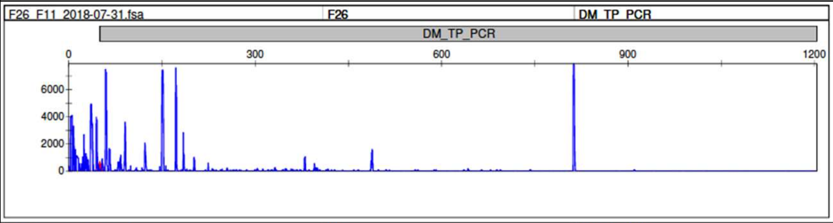
1	T19301	A	IV-65	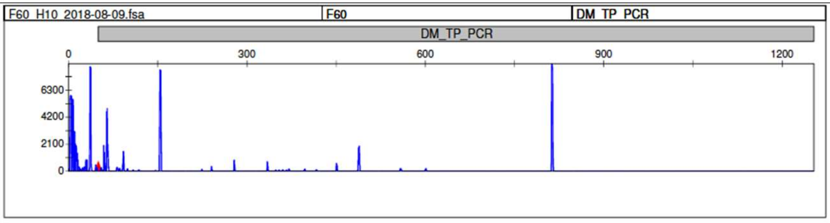
1	T19319	A	IV-72	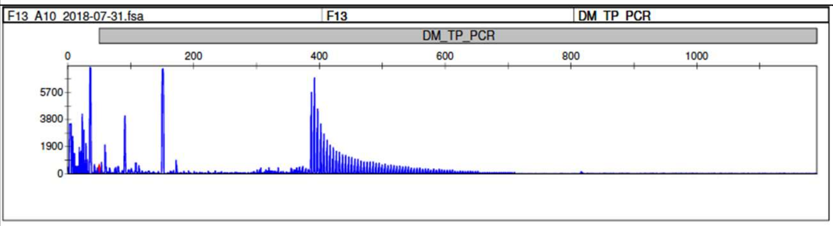
1	T19300	A	IV-73	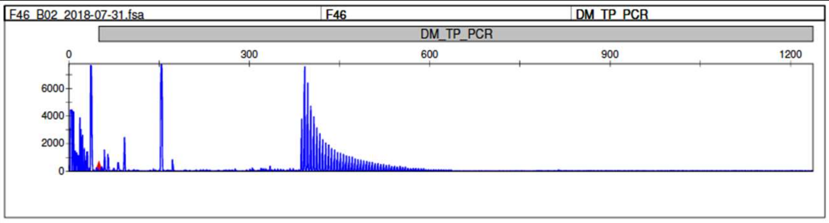
1	T19063	A	IV-97	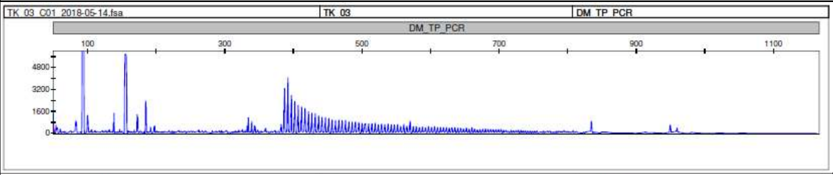
1	T18881	A	IV-98	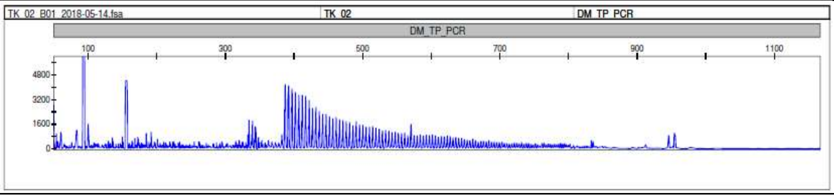

Family	DNA number	Disease Status	Pedigree Number	TTTCA RP-PCR trace
1	T19295	A	IV-103	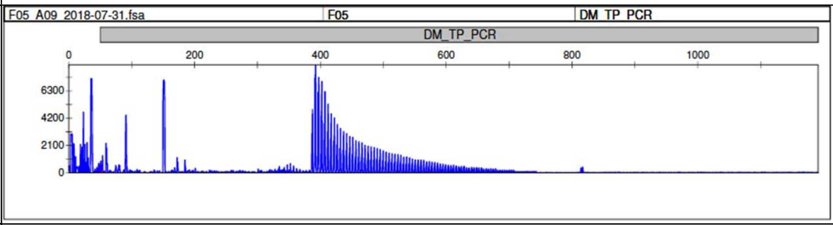
1	T19342	U	IV-105	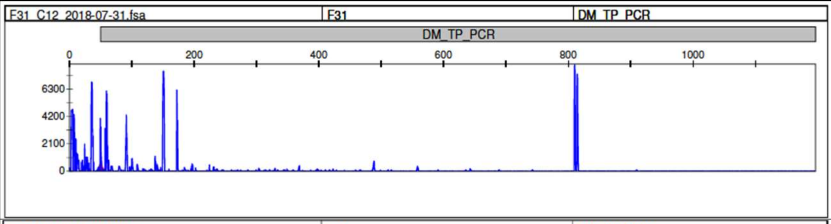
1	T20397	N	IV-106	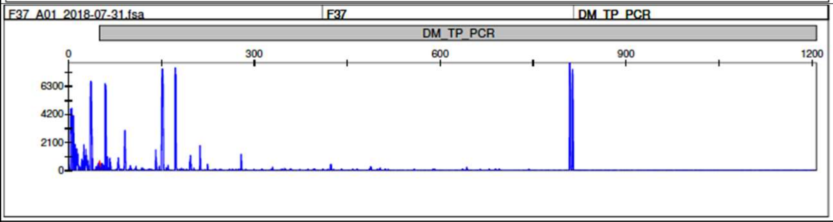
1	T20396	N	IV-107	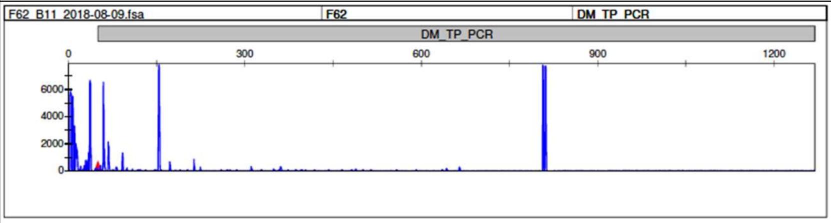
1	T19332	A	V-12	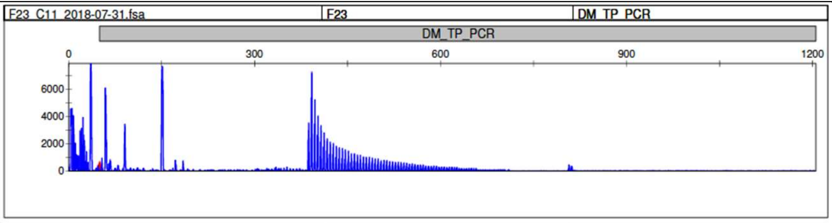
1	T19326	U	V-20	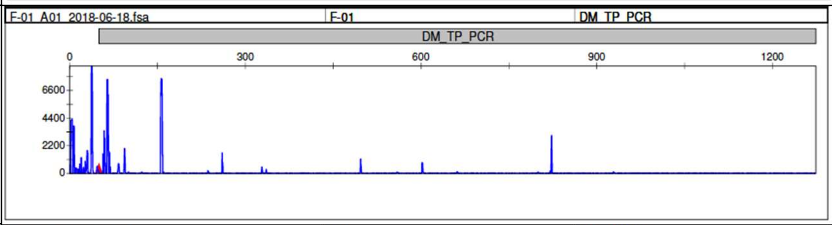
1	T19322	A	V-23	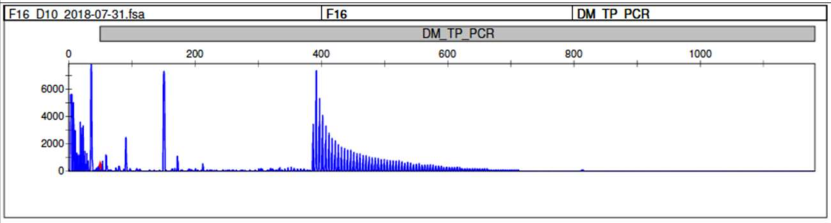
1	T19343	A	V-24	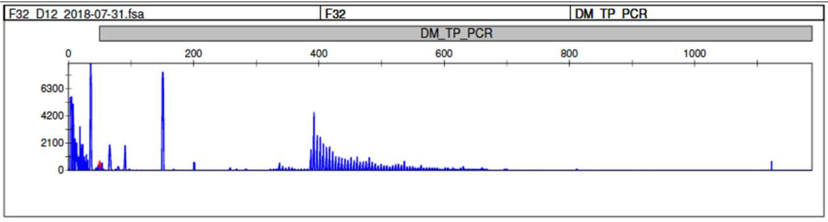

Family	DNA number	Disease Status	Pedigree Number	TTTCA RP-PCR trace
1	T19312	A	V-25	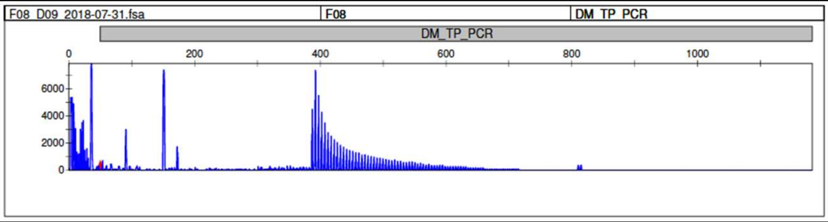
1	T19313	A	V-27	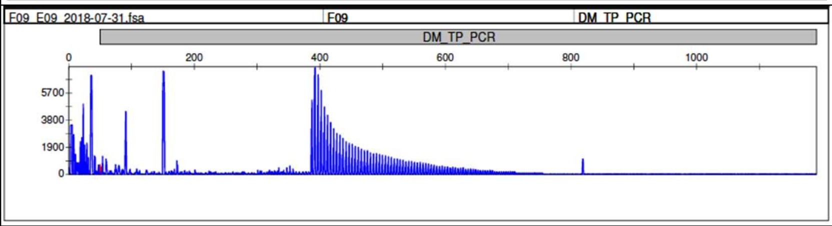
1	T19317	A	V-46	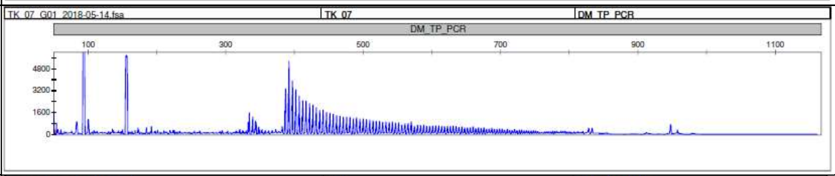
1	T20002	A	V-114	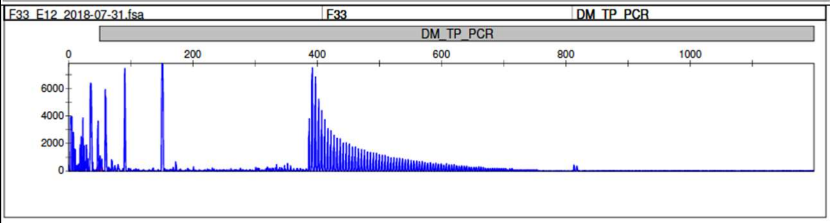
1	T20356	N	V-116	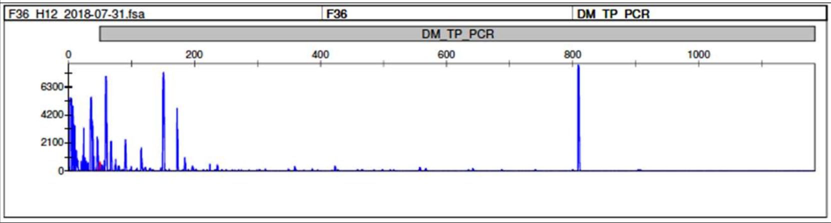
1	T19339	A	V-118	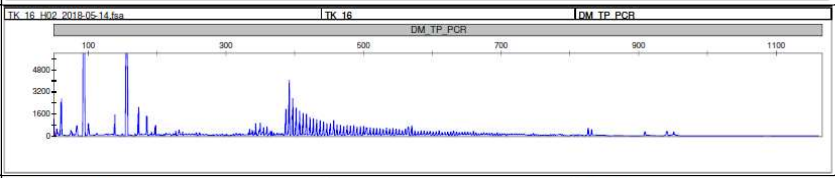
1	T19206	A	V-123	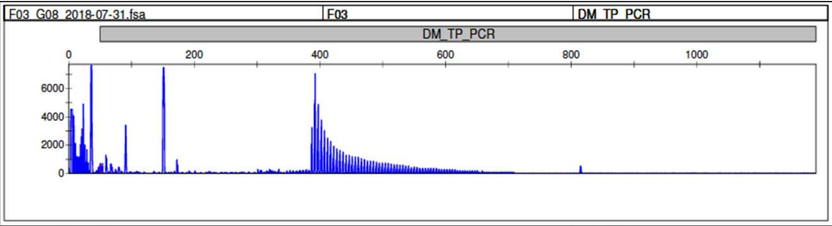
1	T26100	A	V-124	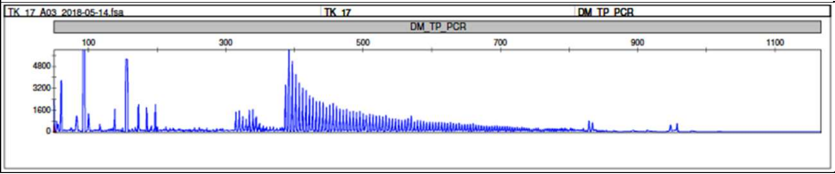

Family	DNA number	Disease Status	Pedigree Number	TTTCA RP-PCR trace
1	T19341	A	V-134	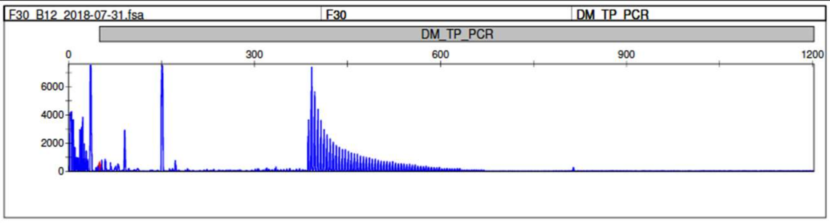
1	T20219	A	V-135	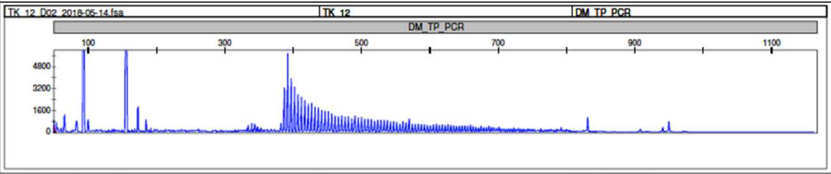
1	T19338	N	V-147	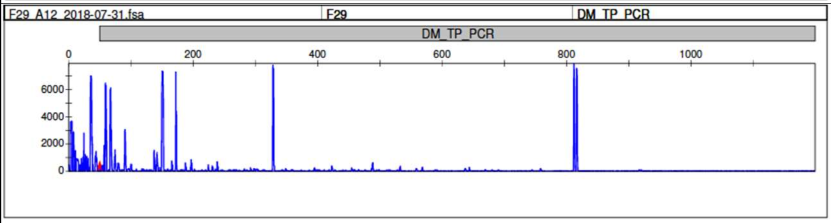
1	T19321	A	V-149	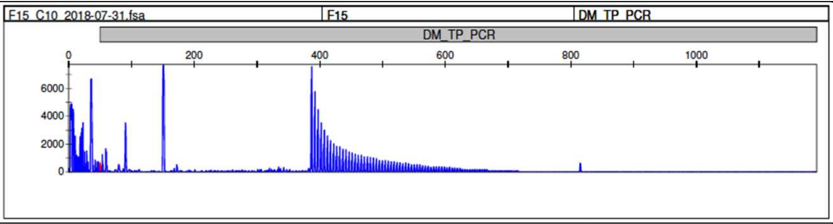
1	T19018	A	V-160	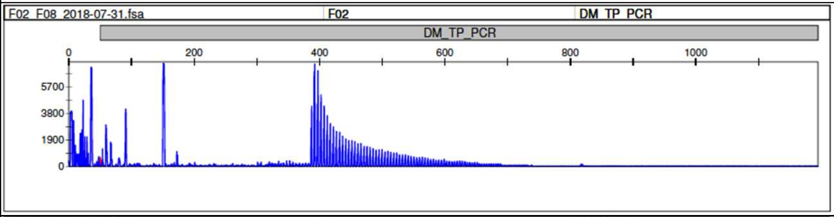
1	T18880	A	V-161	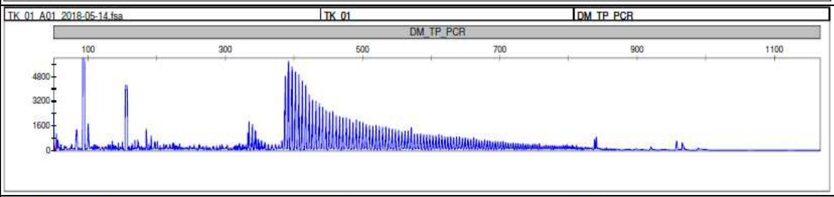
2	T23990	A	III-1	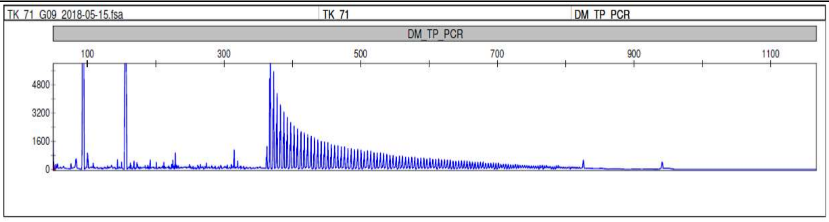
2	T23989	A	IV-2	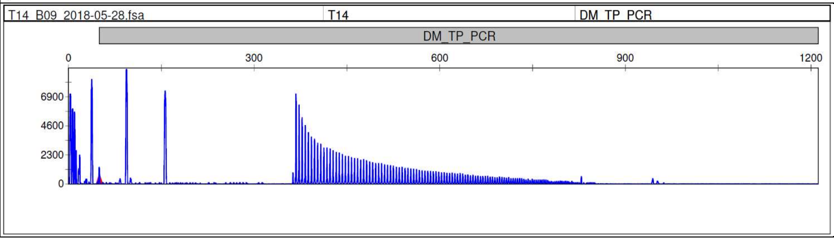

Family	DNA number	Disease Status	Pedigree Number	TTTCA RP-PCR trace
3	PM0198	N	II-1	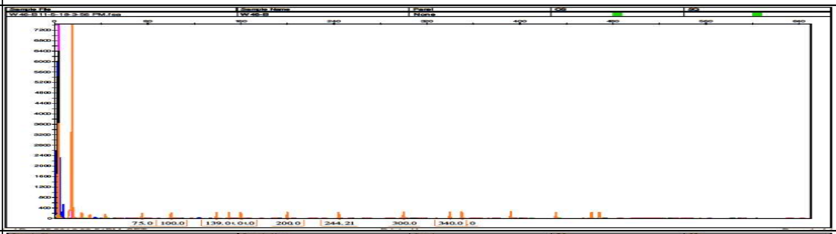
3	PM0199	A	II-2	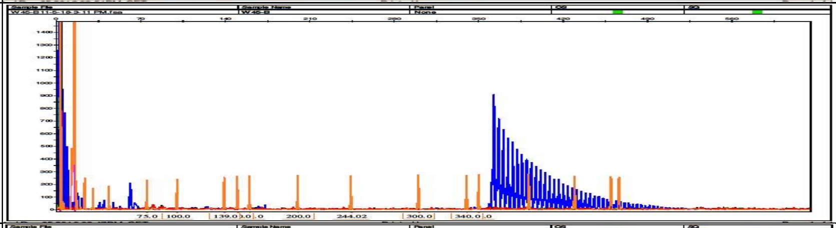
3	PM0197	A	II-3	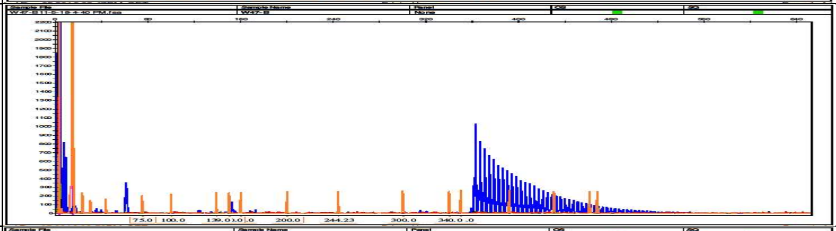
3	PM0200	N	II-4	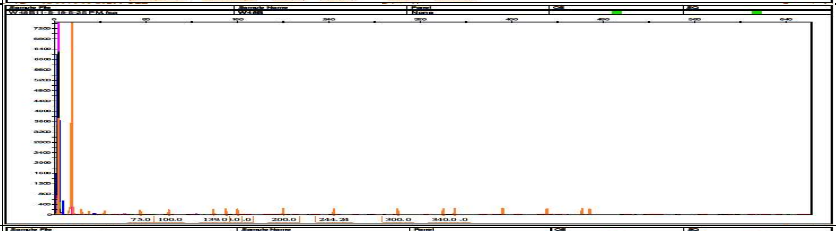
3	PM0195	A	III-1	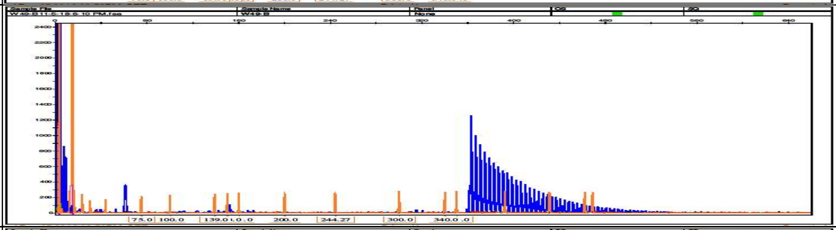
3	PM0196	A	III-2	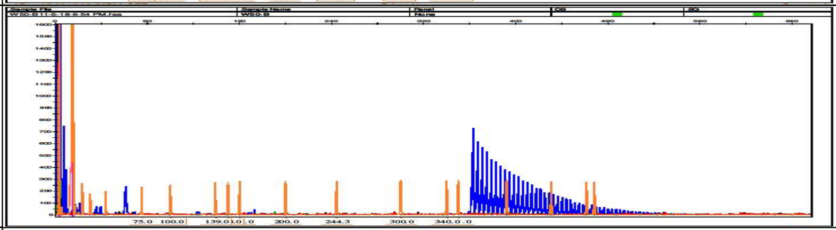
4	60122-619V	A	III-14	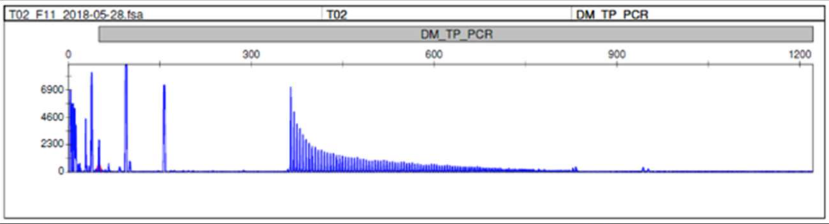
4	60121-618V	A	IV-4	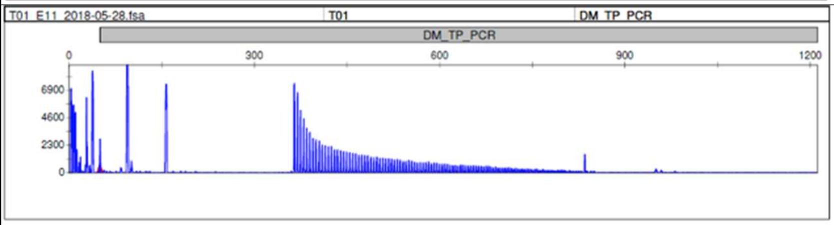

Family	DNA number	Disease Status	Pedigree Number	TTTCA RP-PCR trace
4	60124-093C	A	V-2	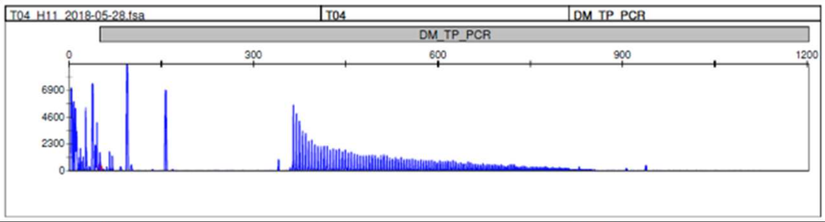
5	60530-F10N	A	III-37	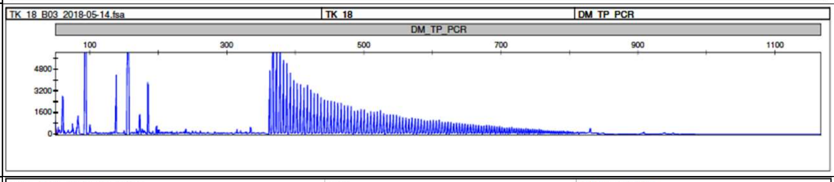
5	60056	A	III-44	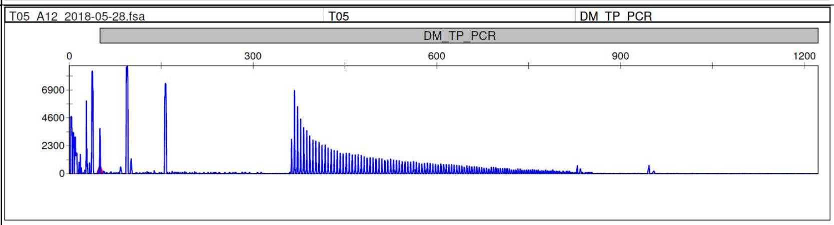
5	60057	A	IV-13	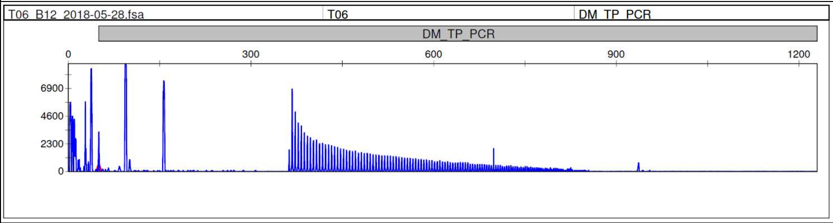
5	60058	N	IV-21	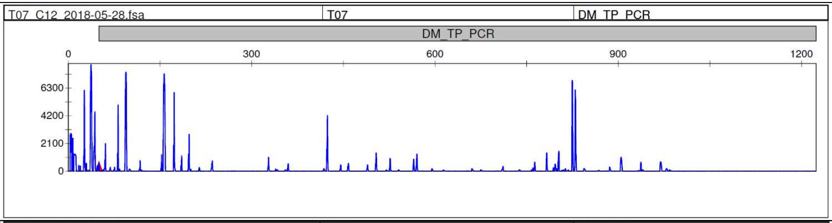
5	60531-F11N	A	IV-29	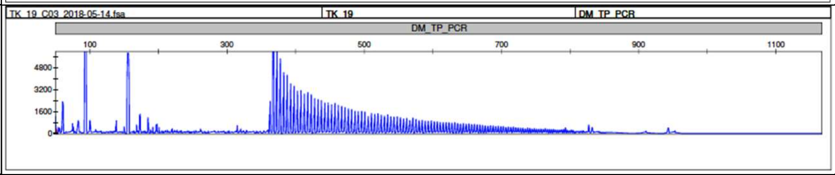
6	60059	A	V-8	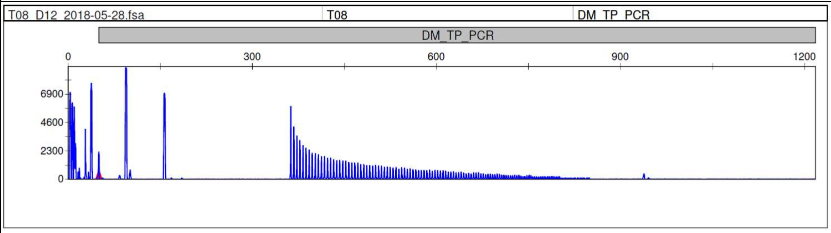
6	60060	N	V-9	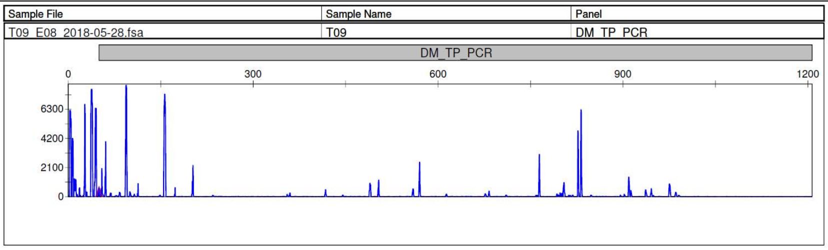

Family	DNA number	Disease Status	Pedigree Number	TTTCA RP-PCR trace
7	60062	A	IV-1	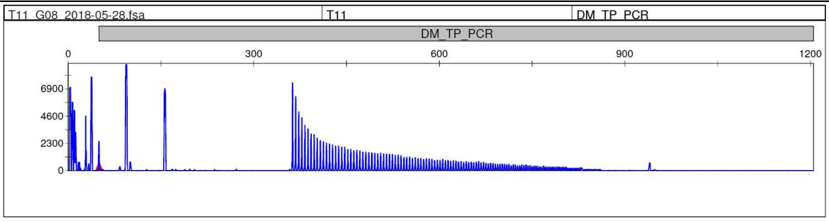
7	60061	A	V-1	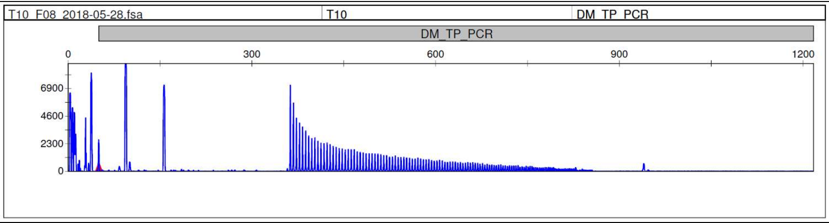
8	60176	A	III-2	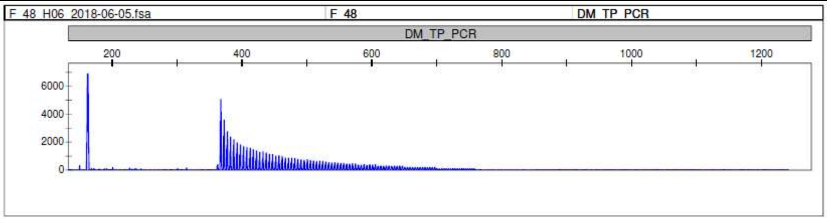
8	60177	A	III-3	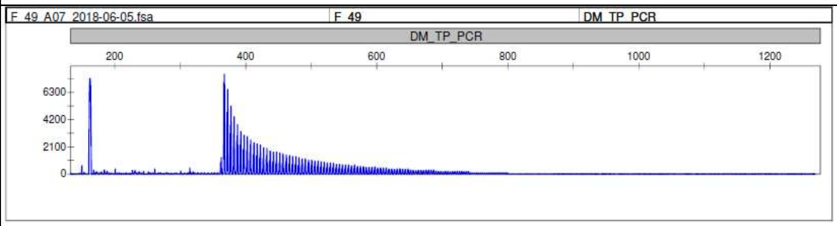
8	60178	A	III-5	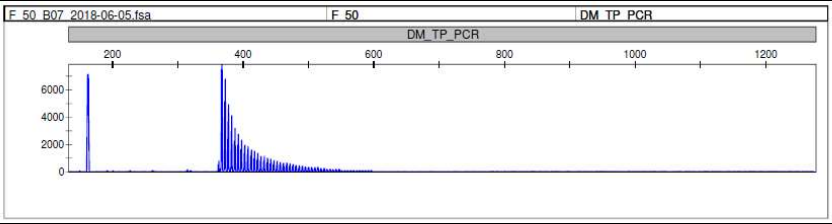
8	60179	N	III-6	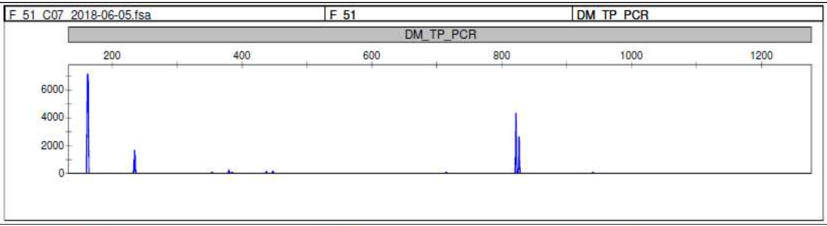
8	60180	N	III-7	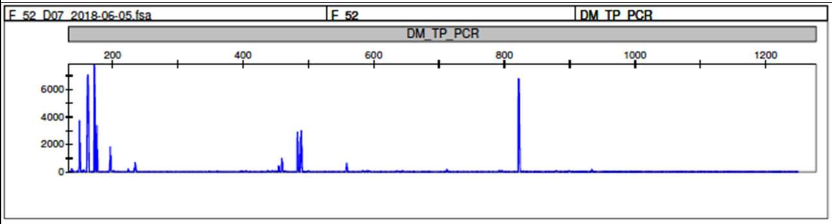
8	60183	A	IV-4	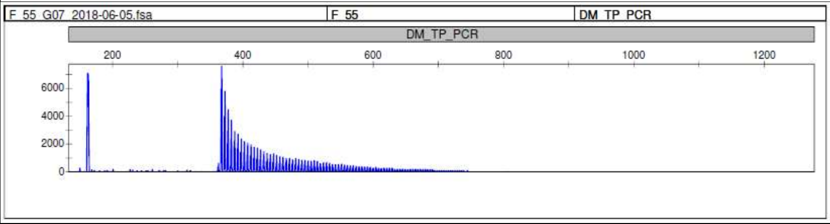

Family	DNA number	Disease Status	Pedigree Number	TTTCA RP-PCR trace
8	60184	A	IV-6	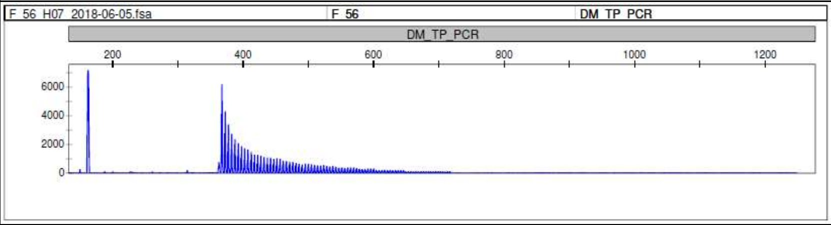
8	60185	N	IV-7	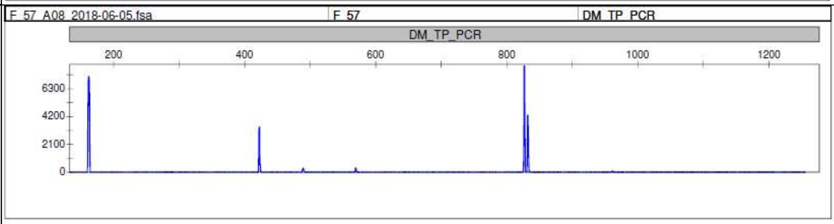
9	60211	N	III-1	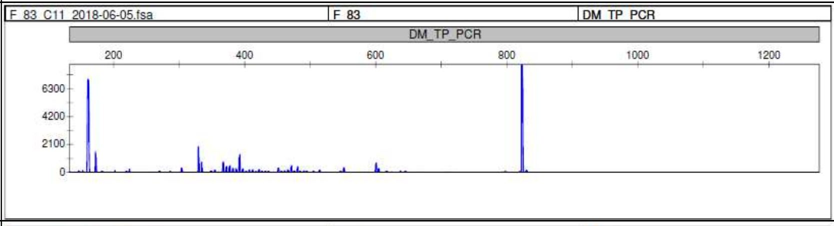
9	60212	A	III-2	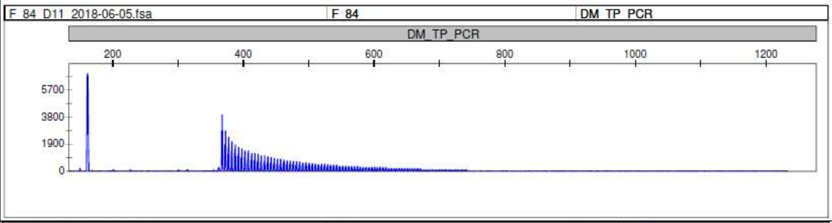
9	60213	A	III-3	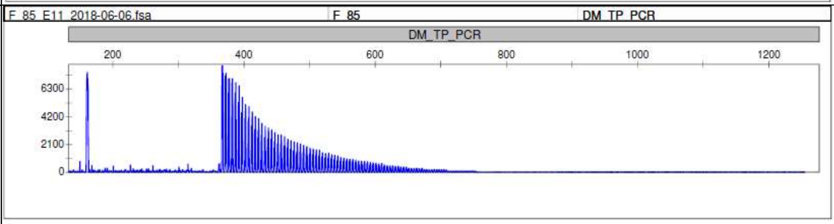
9	60214	A	III-6	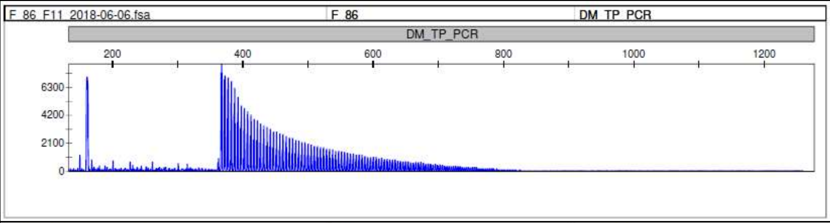
9	60215	A	III-9	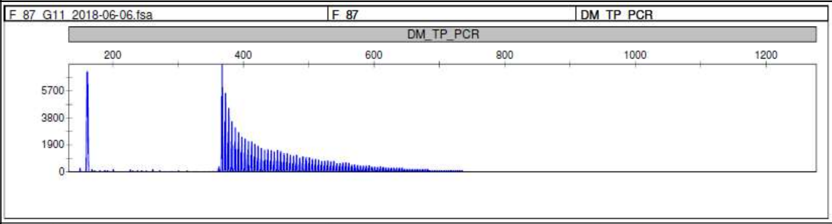
9	60216	A	III-10	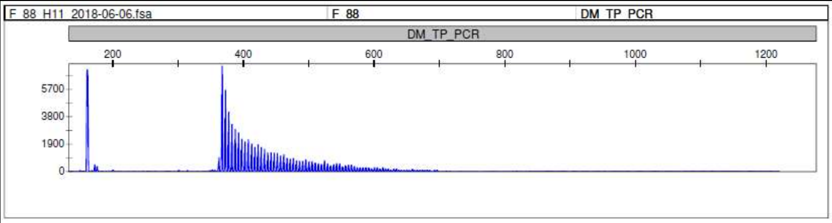

Family	DNA number	Disease Status	Pedigree Number	TTTCA RP-PCR trace
9	60217	A	IV-1	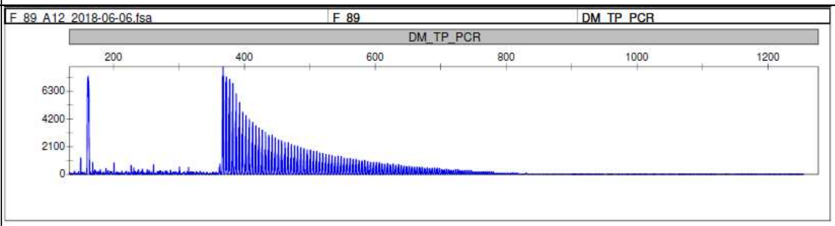
9	60218	A	IV-2	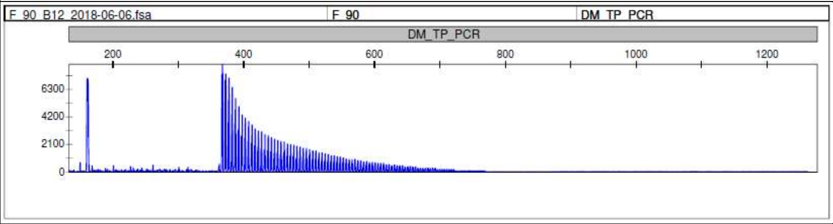
9	60219	A	IV-3	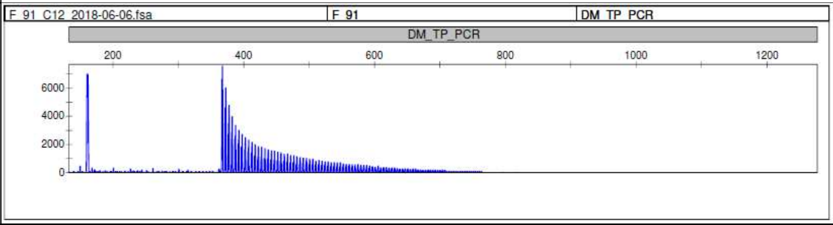
9	60220	A	IV-4	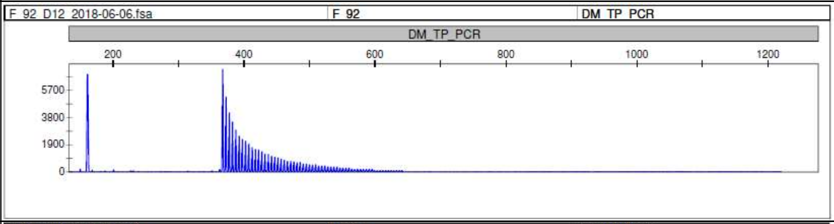
9	60221	A	IV-5	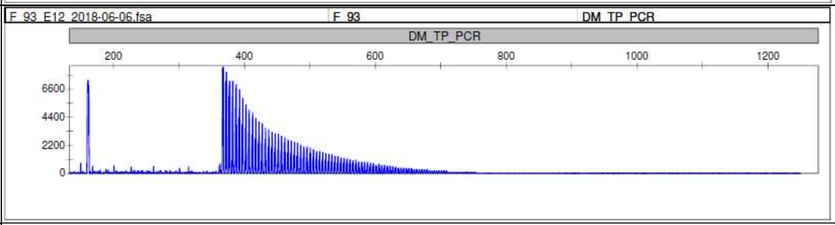
10	60190	A	III-1	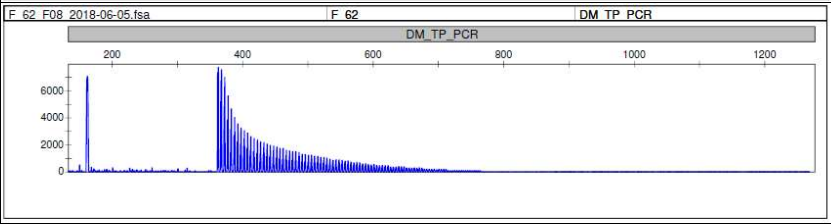
10	60191	N	III-2	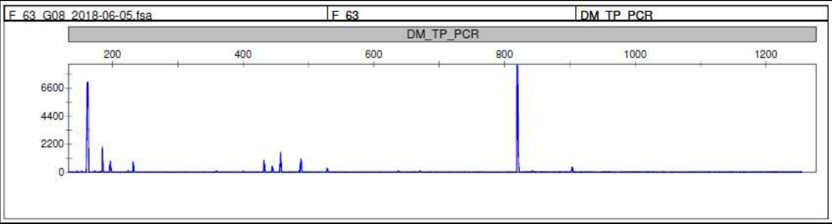
10	60192	A	III-4	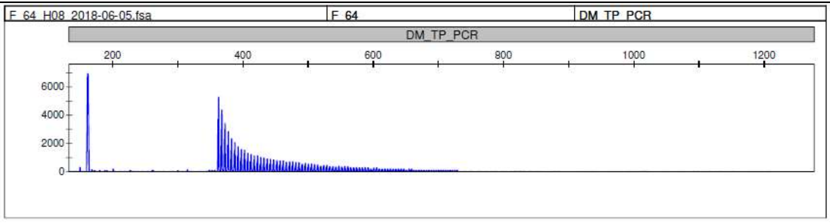

Family	DNA number	Disease Status	Pedigree Number	TTTCA RP-PCR trace
10	60193	N	IV-1	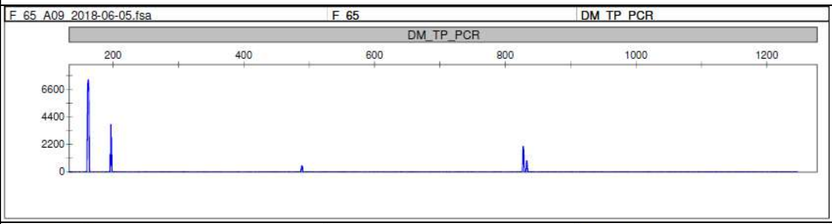
10	60195	A	IV-4	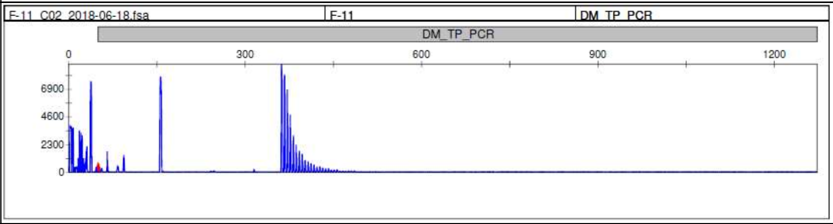
10	60196	A	IV-5	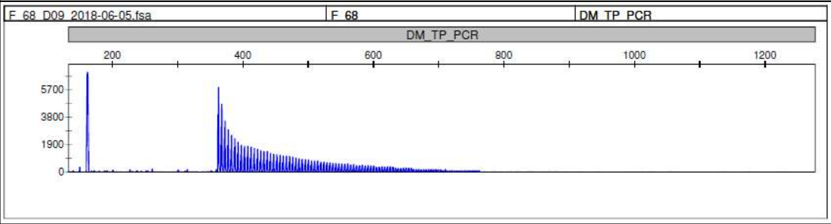
10	60197	N	IV-6	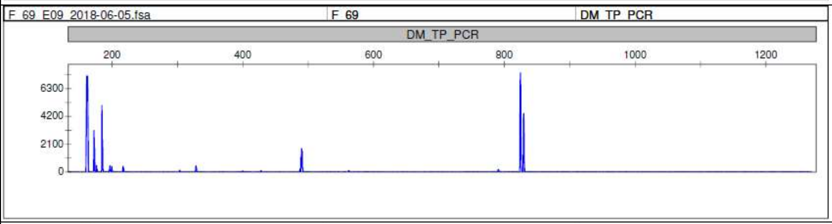
10	60198	A	IV-7	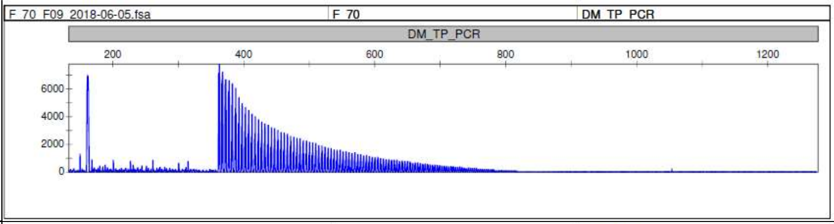
10	60199	A	IV-9	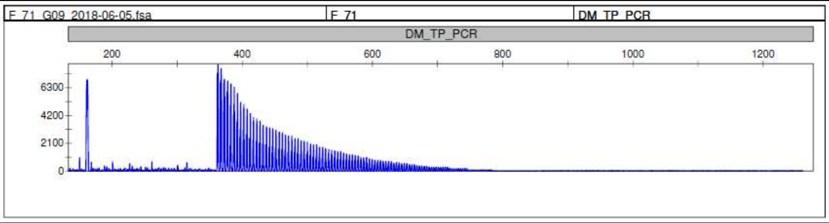
10	60200	A	IV-11	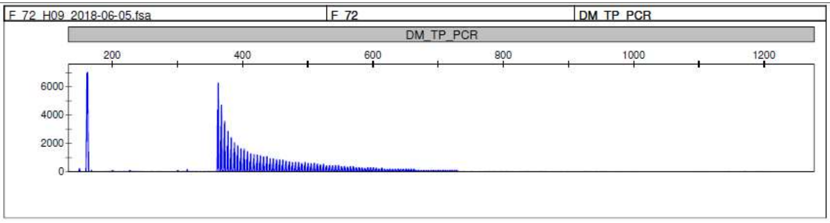
10	60201	N	IV-12	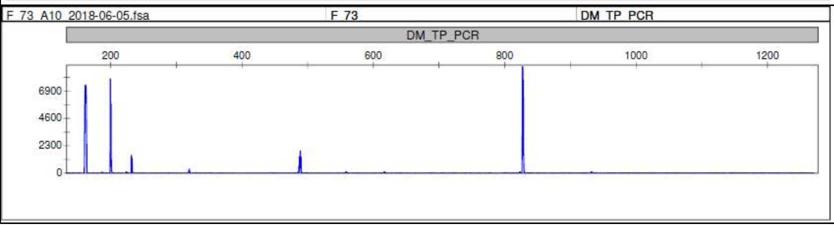

Family	DNA number	Disease Status	Pedigree Number	TTTCA RP-PCR trace
10	60202	N	V-1	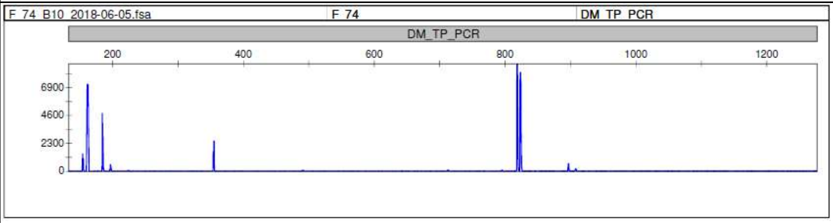
10	60203	A	V-5	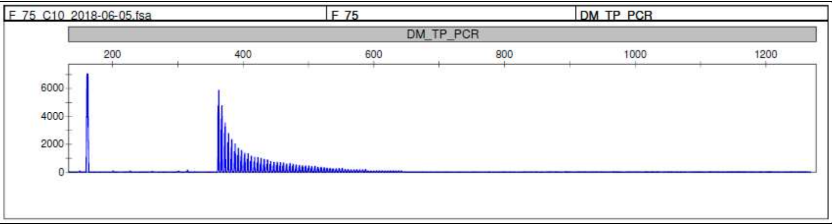
10	60204	A	V-6	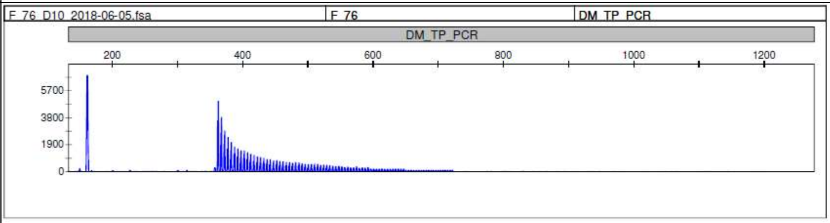
10	60205	N	V-7	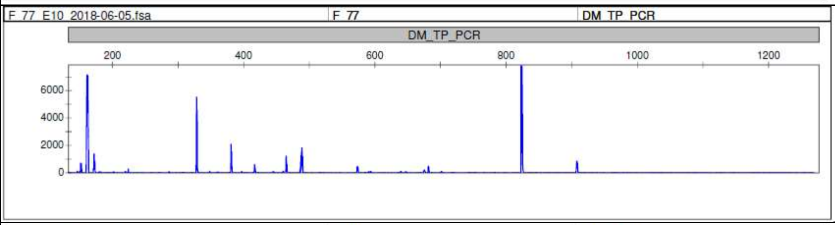
10	60206	A	V-8	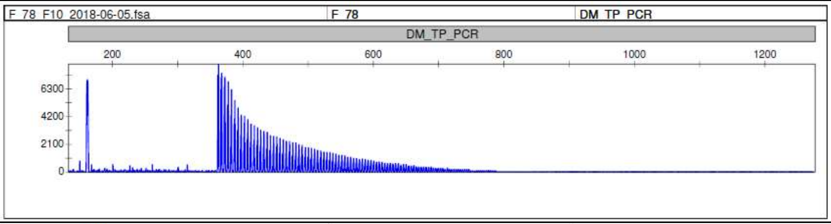
10	60207	N	V-9	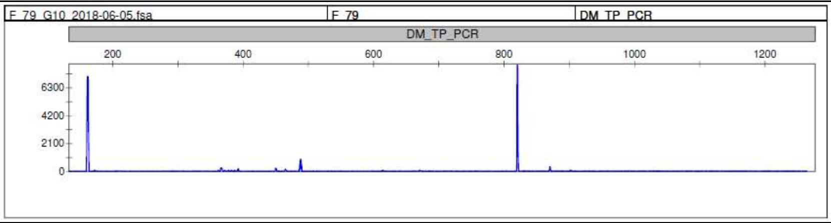
10	60208	A	V-10	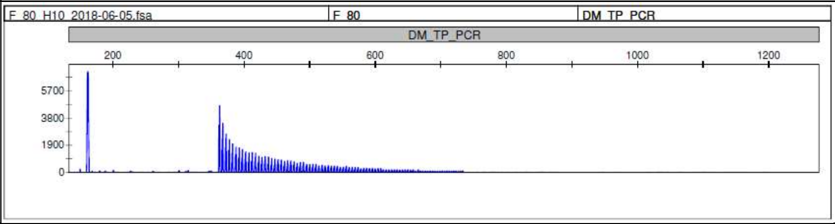
10	60209	N	V-11	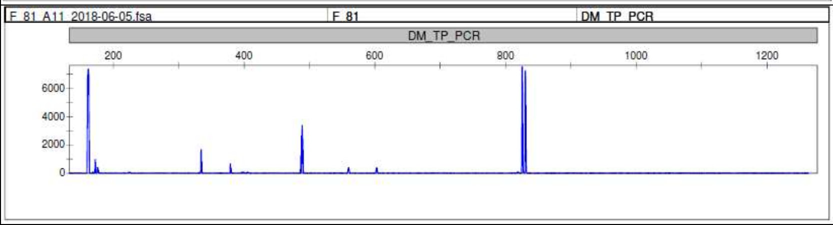

Family	DNA number	Disease Status	Pedigree Number	TTTCA RP-PCR trace
10	60210	N	V-12	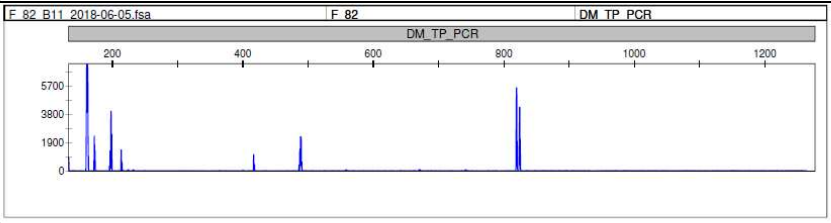
11	60063	N	II-1	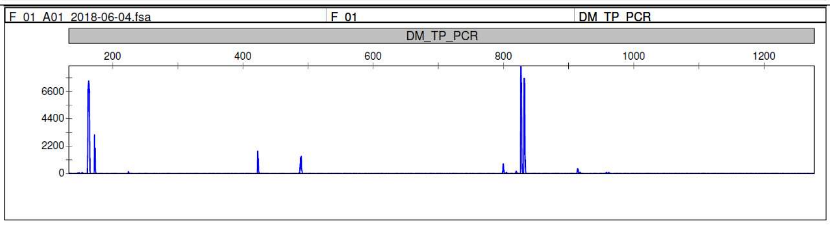
11	60130	A	II-2	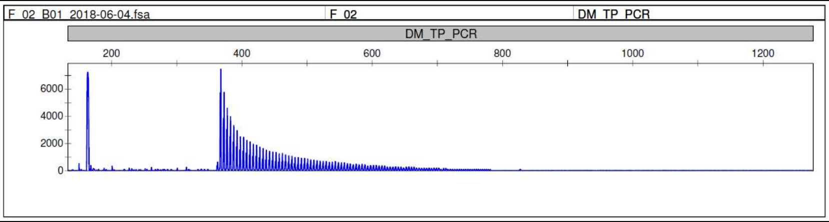
11	60131	A	II-3	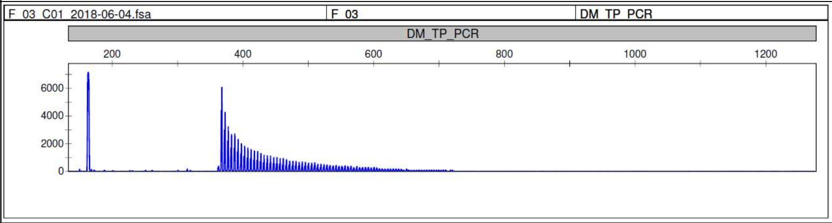
11	60132	U	II-5	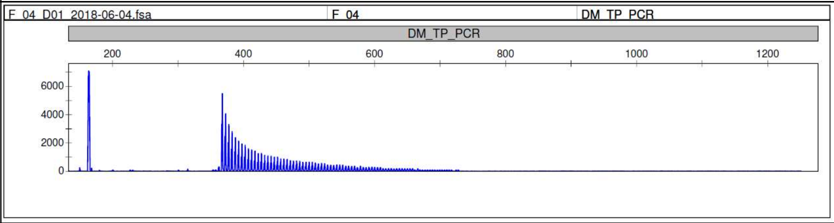
11	60133	A	II-6	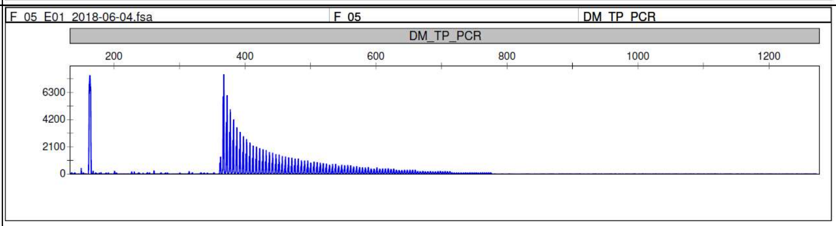
11	60134	U	II-8	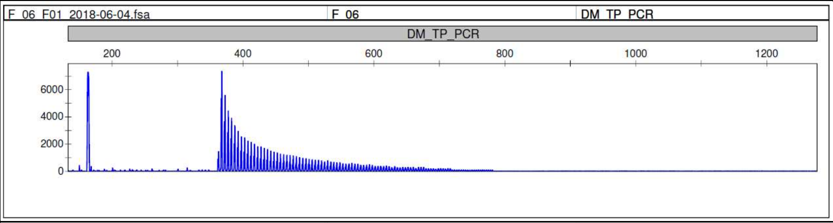
11	60135	N	II-10	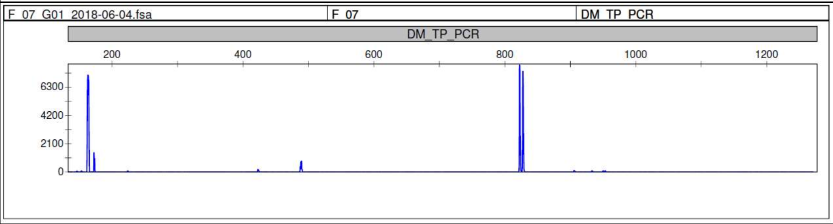

Family	DNA number	Disease Status	Pedigree Number	TTTCA RP-PCR trace
11	60136	A	II-11	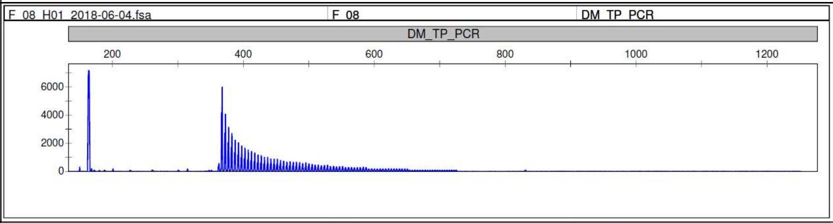
11	60137	A	III-1	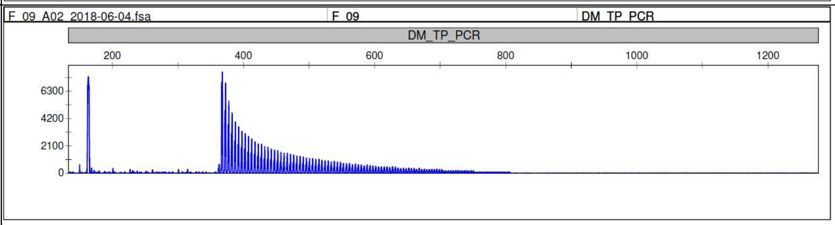
11	60138	A	III-4	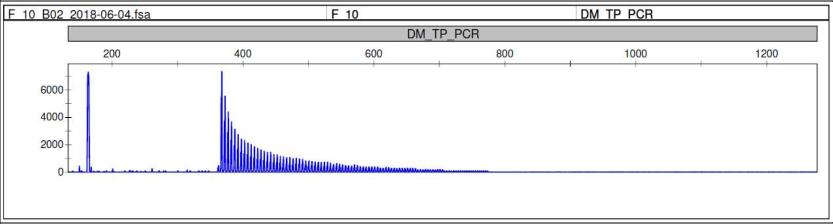
11	60139	A	III-8	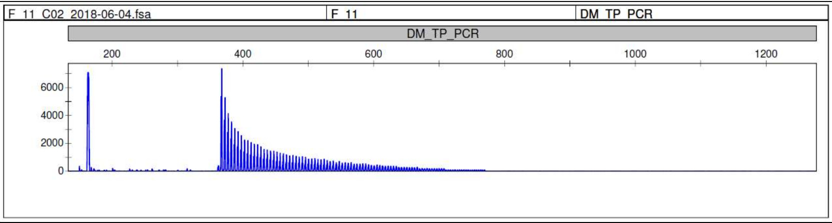
11	60140	A	III-10	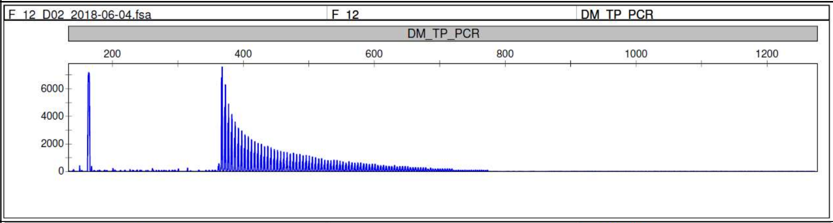
11	60141	A	III-13	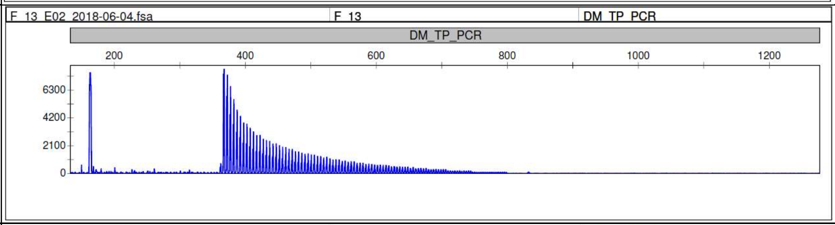
12	60151	A	III-1	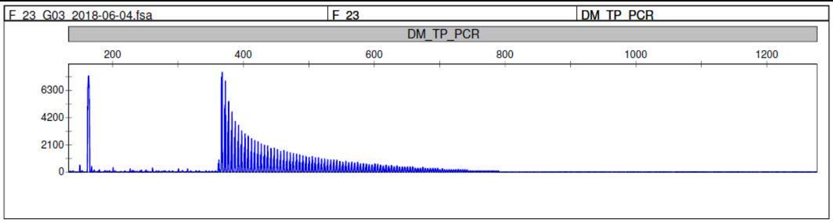
12	60153	A	III-4	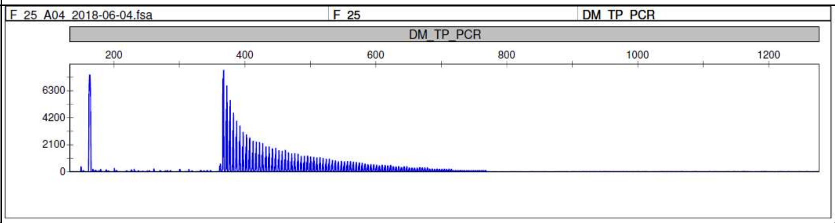

Family	DNA number	Disease Status	Pedigree Number	TTTCA RP-PCR trace
12	60155	A	III-6	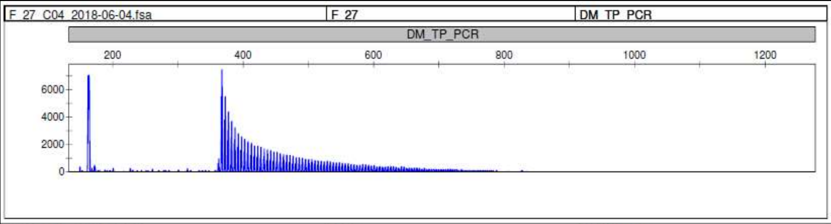
12	60156	N	III-7	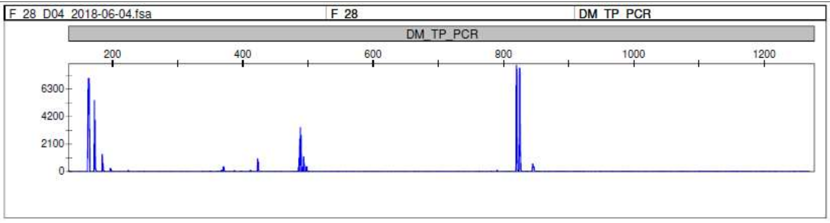
12	60157	A	III-8	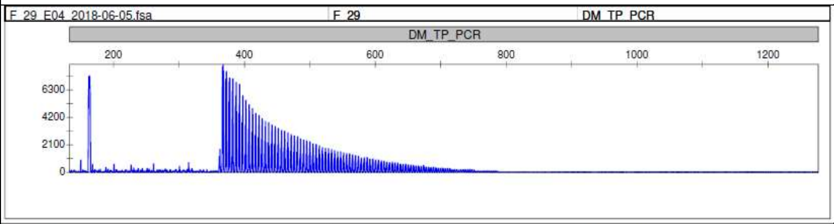
12	60158	N	III-10	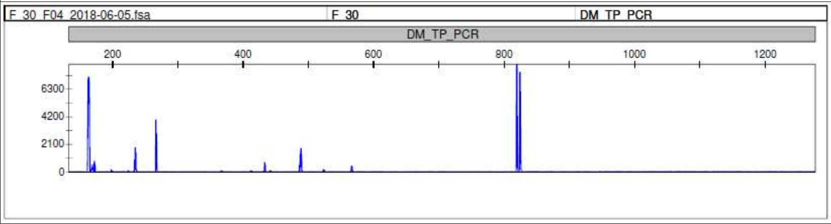
12	60159	A	III-11	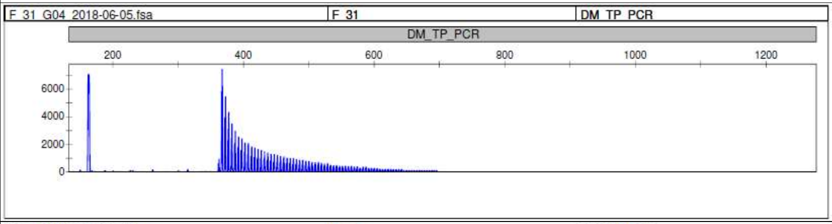
12	60160	A	III-12	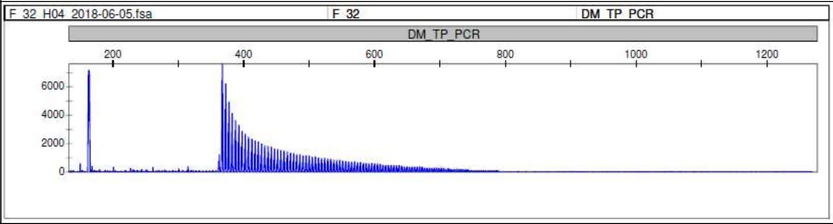
12	60161	N	III-13	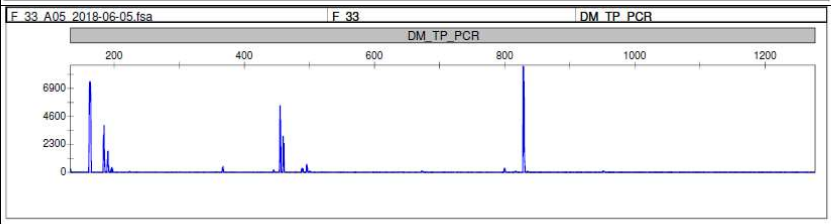
12	60162	A	IV-1	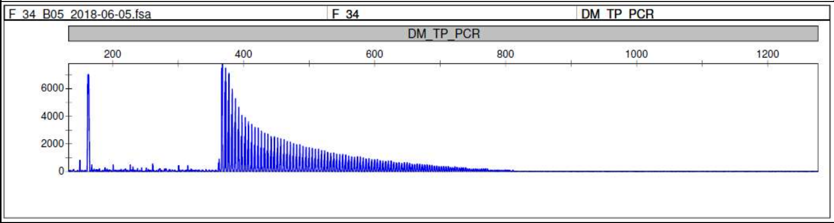

Family	DNA number	Disease Status	Pedigree Number	TTTCA RP-PCR trace
12	60163	A	IV-2	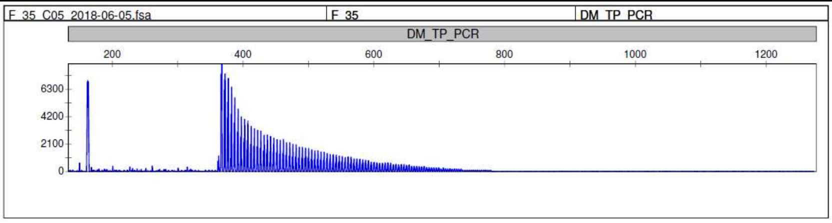
12	60164	A	IV-4	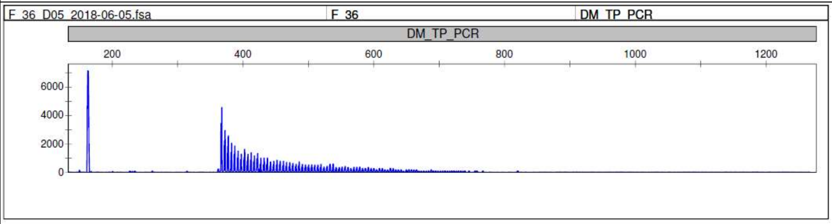
12	60165	N	IV-5	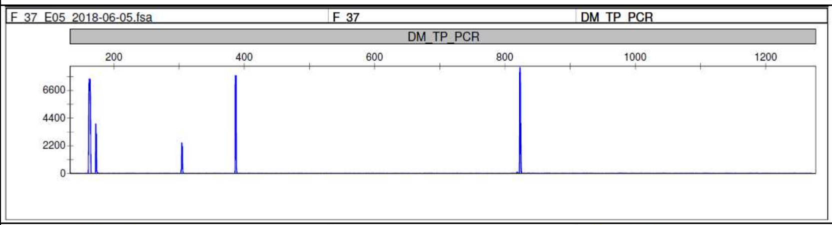
12	60167	N	IV-7	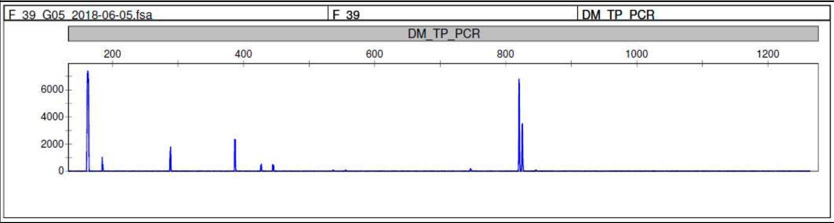
12	60168	N	IV-8	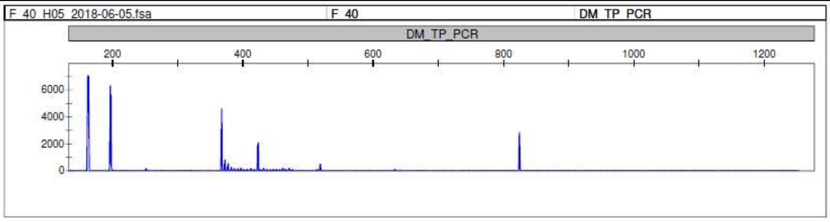
12	60169	A	IV-9	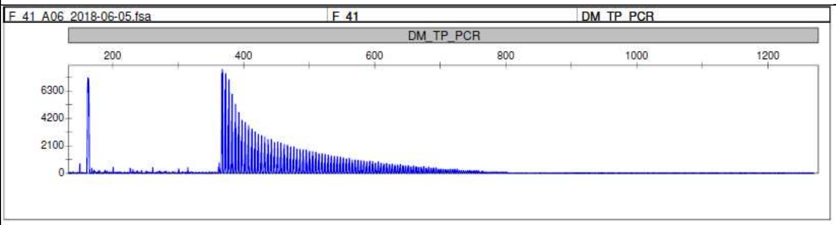
12	60170	A	IV-10	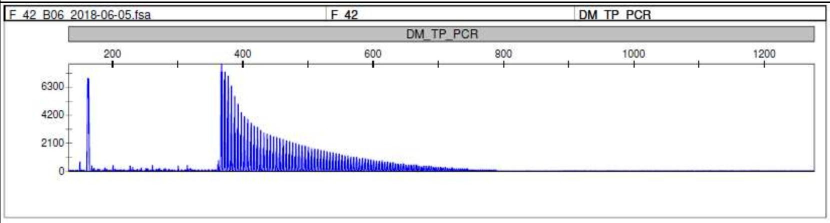
12	60171	A	IV-11	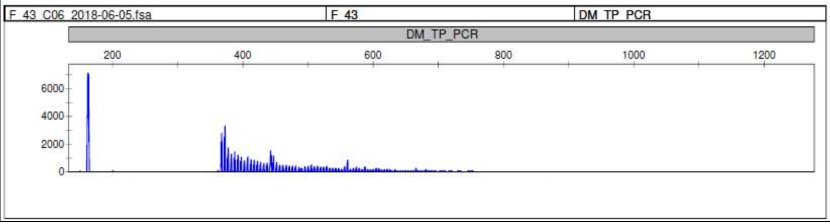

Family	DNA number	Disease Status	Pedigree Number	TTTCA RP-PCR trace
12	60172	A	IV-12	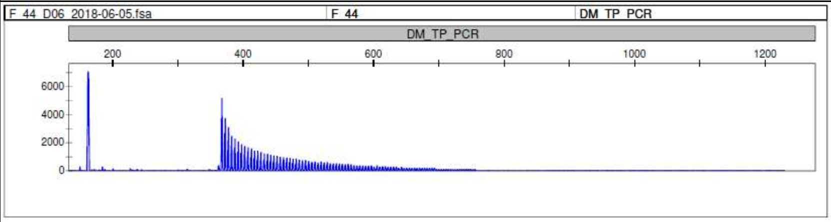
12	60173	A	IV-13	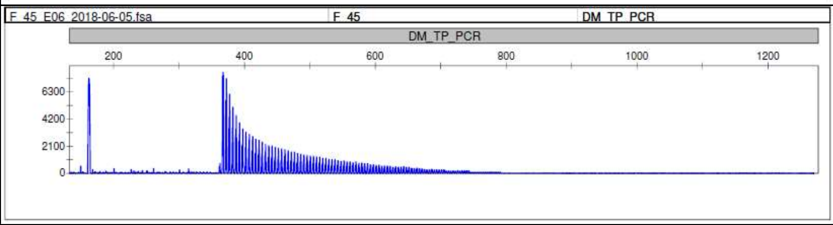
12	60174	A	V-1	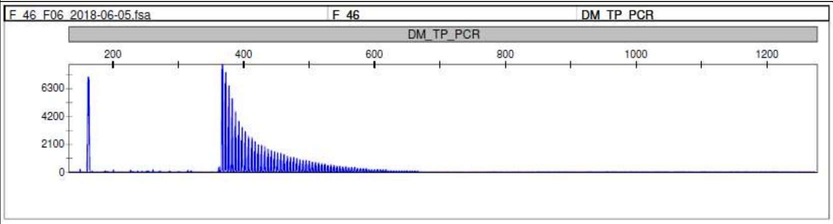
12	60175	N	V-2	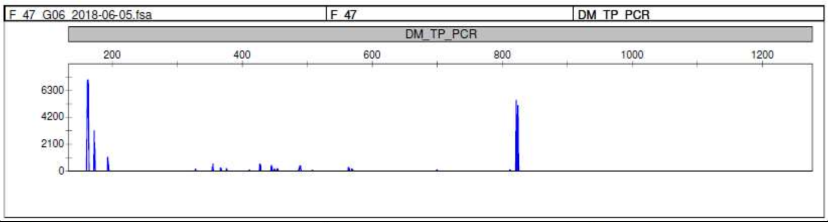
13	60142	N	II-1	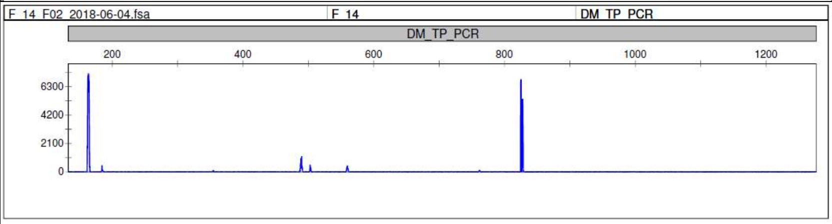
13	60143	A	II-2	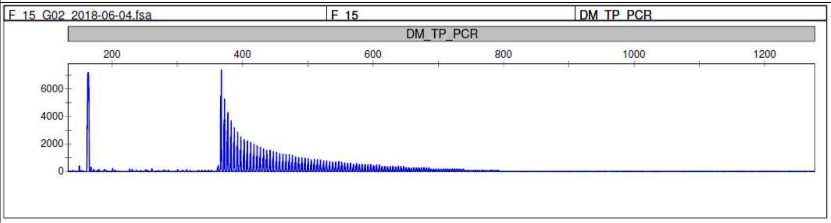
13	60144	A	III-2	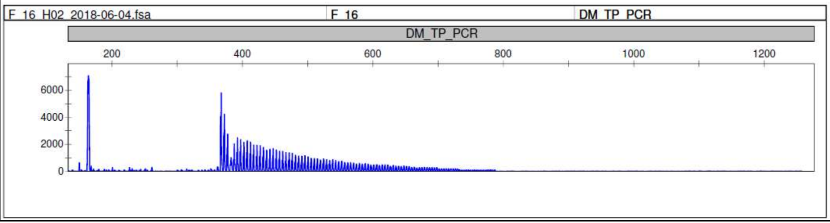

Family	DNA number	Disease Status	Pedigree Number	TTTCA RP-PCR trace
14	60186	N	I-1	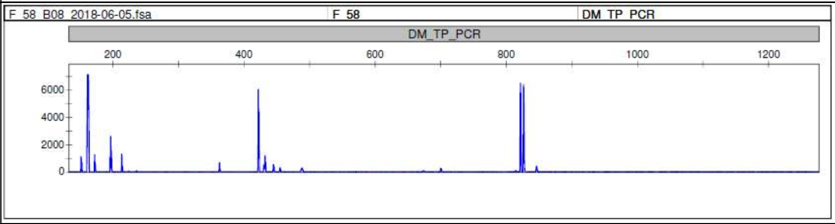
14	60187	A	I-2	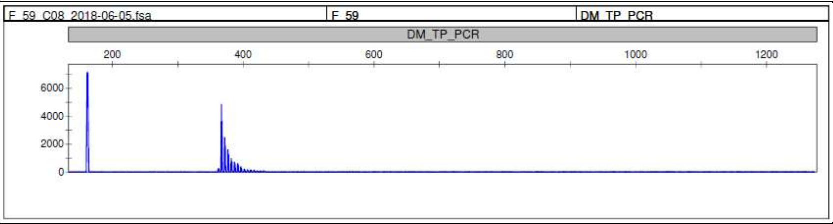
14	60188	A	II-2	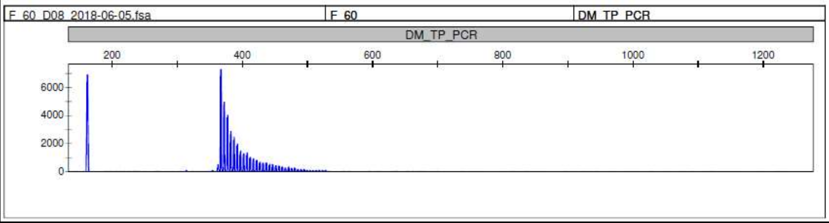
14	60189	N	II-3	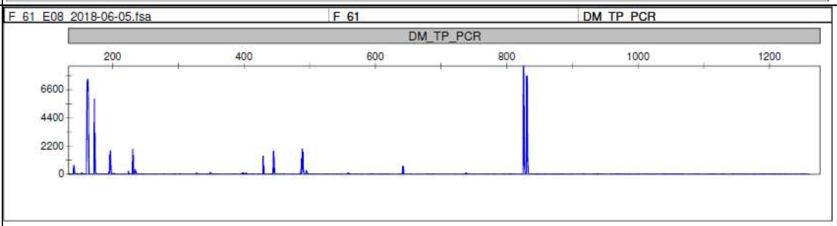
15	60145	A	I-1	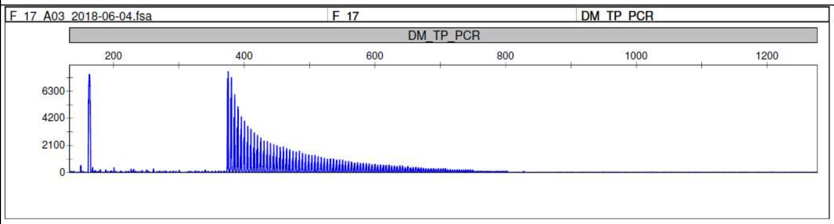
15	60146	N	I-2	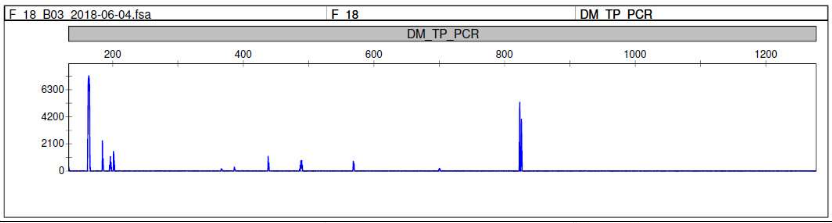
15	60147	N	II-1	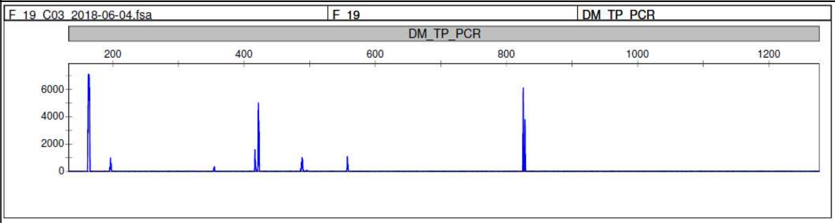
15	60148	A	II-2	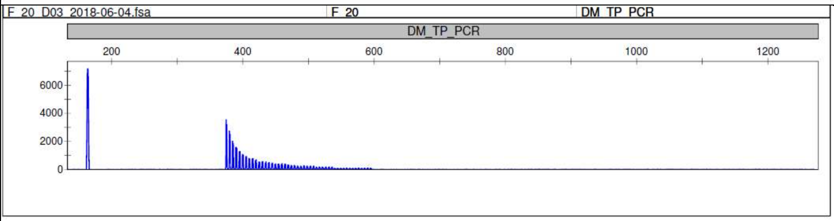

Family	DNA number	Disease Status	Pedigree Number	TTTCA RP-PCR trace
15	60149	A	II-3	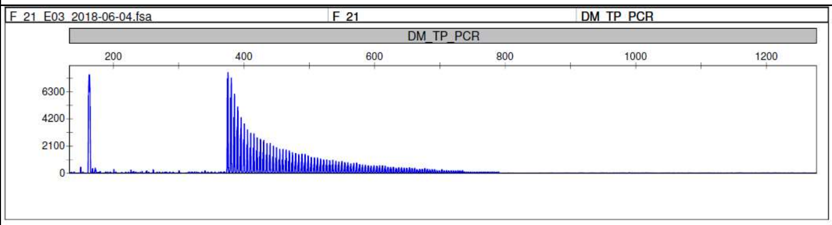
15	60150	A	III-1	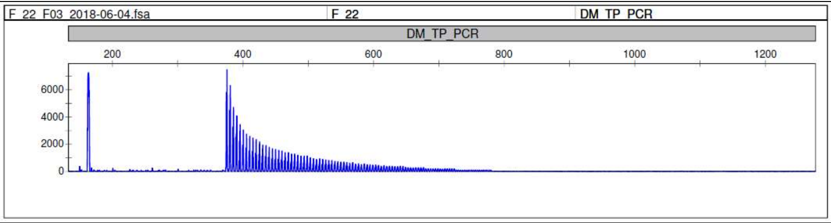
16	60037	A	III-5	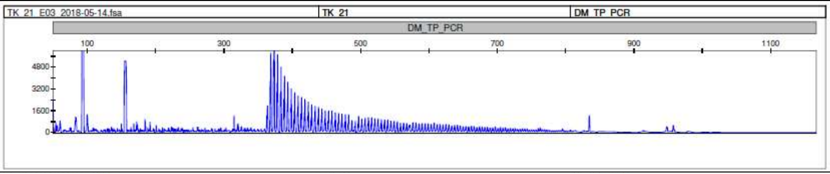
16	60036	N	IV-5	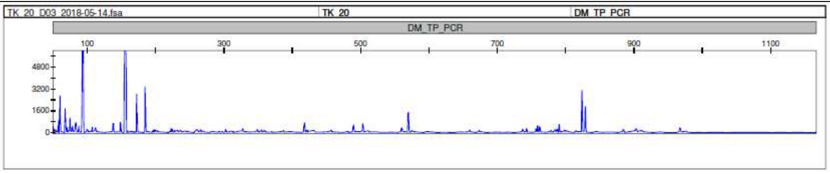
16	60038	A	IV-10	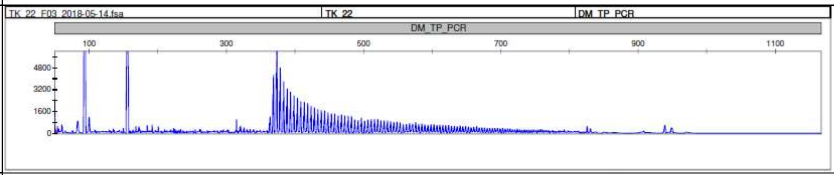
16	60039	A	IV-13	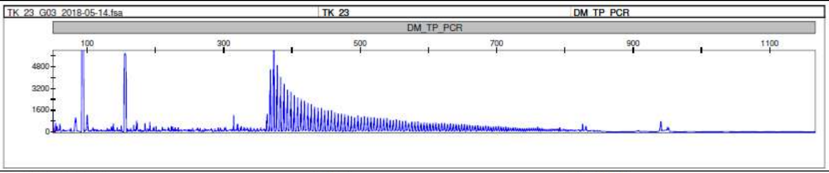
16	60040	A	IV-17	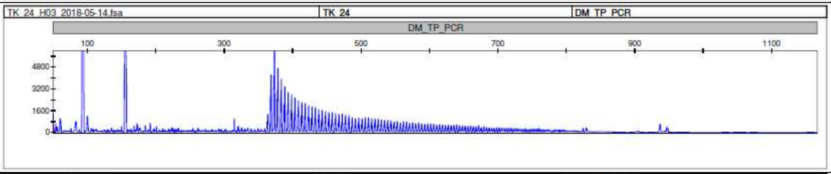
16	60041	A	IV-19	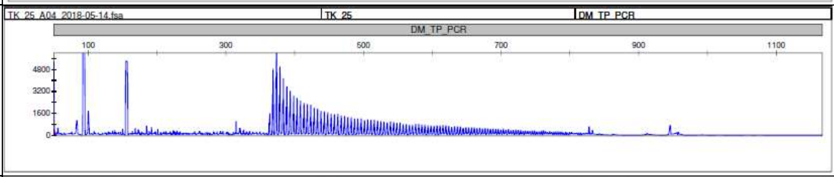
16	60042	N	IV-20	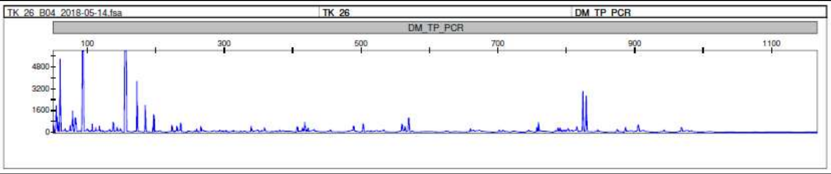

Family	DNA number	Disease Status	Pedigree Number	TTTCA RP-PCR trace
16	60043	N	IV-21	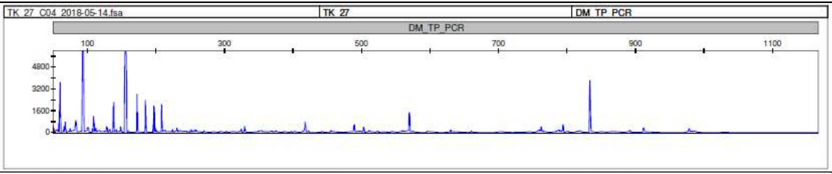
16	60045	A	IV-22	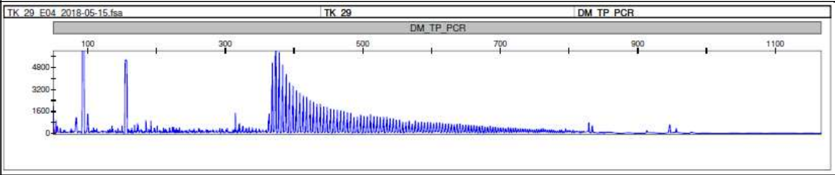
16	60046	A	V-21	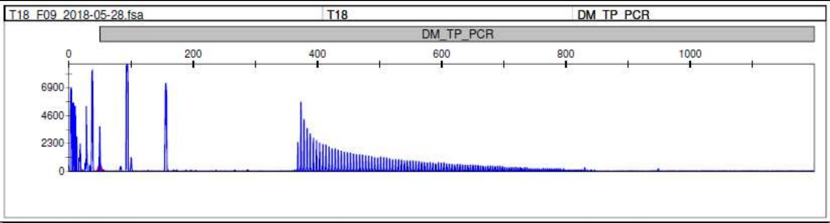
16	60044	U	V-33	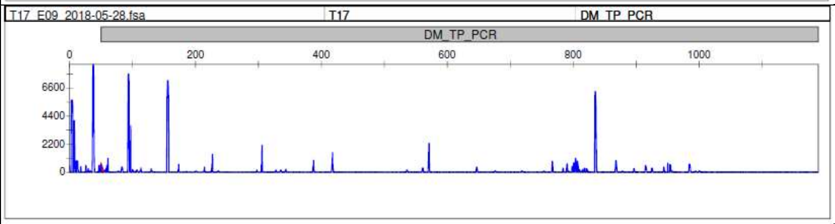
17	60222	A	III-5	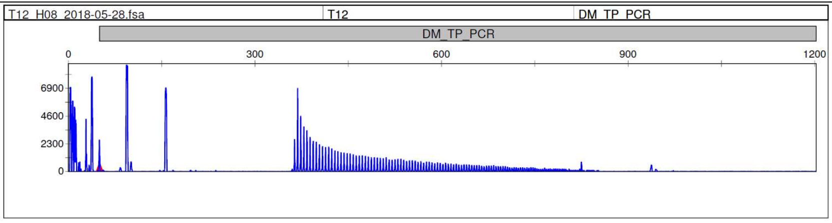
17	60094	A	IV-4	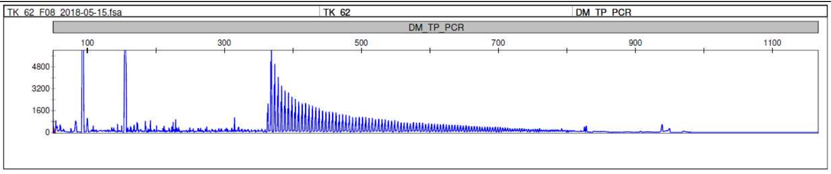
17	60223	N	IV-5	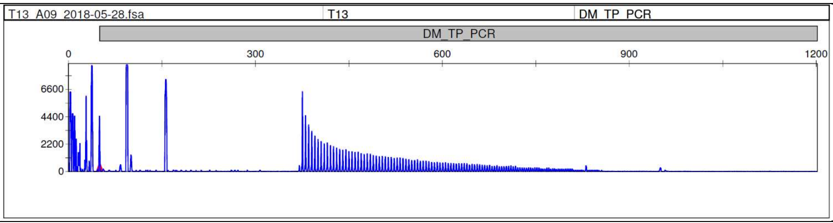
18	15	A	IV-3	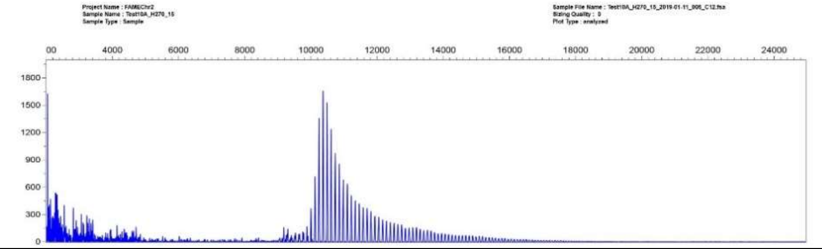

Family	DNA number	Disease Status	Pedigree Number	TTTCA RP-PCR trace
18	48	A	IV-6	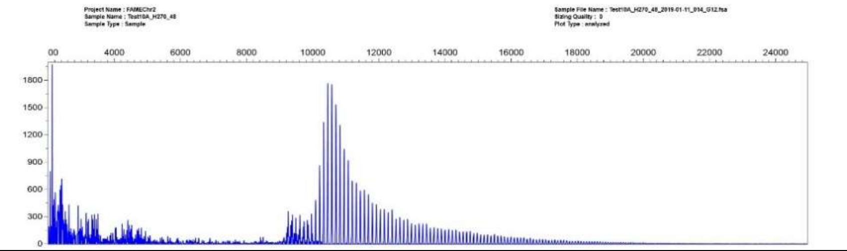
18	16	A	IV-14	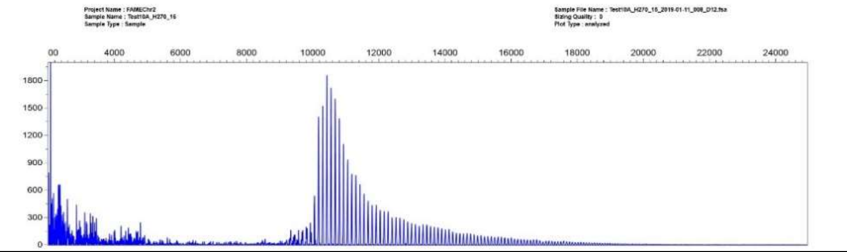
18	47	A	V-1	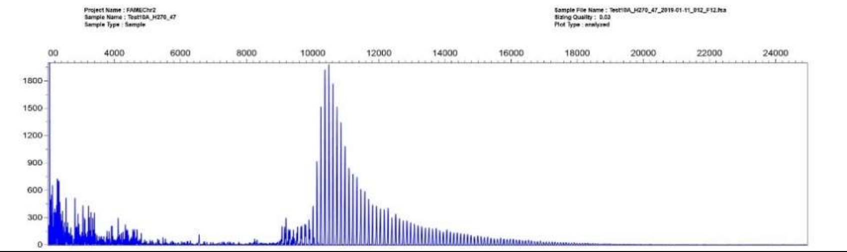
18	46	A	V-2	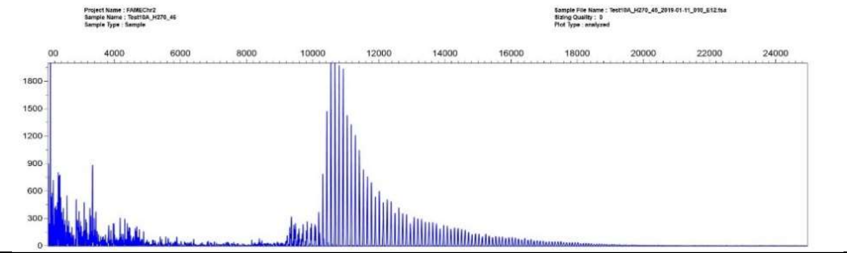
19	1	A	III-2	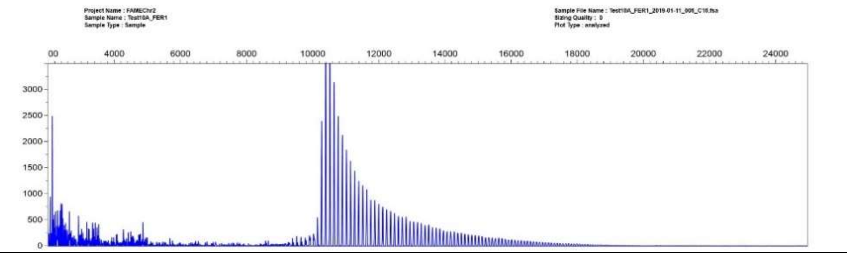
19	5	A	III-3	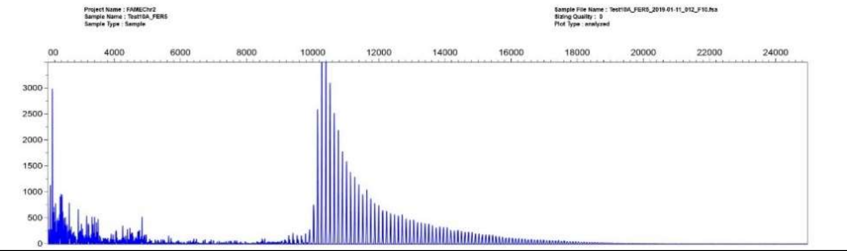
19	9	A	III-5	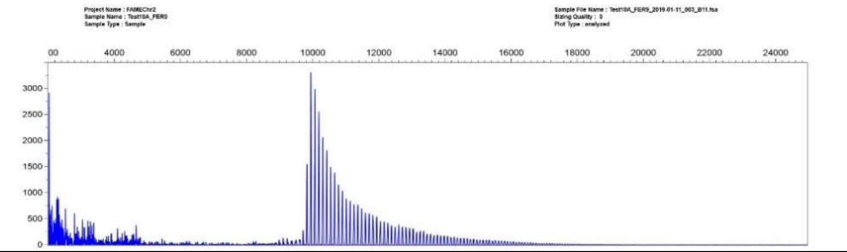

Family	DNA number	Disease Status	Pedigree Number	TTTCA RP-PCR trace
19	14	A	III-8	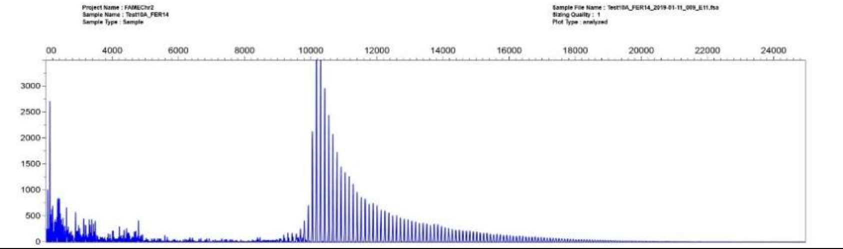
19	16	A	III-10	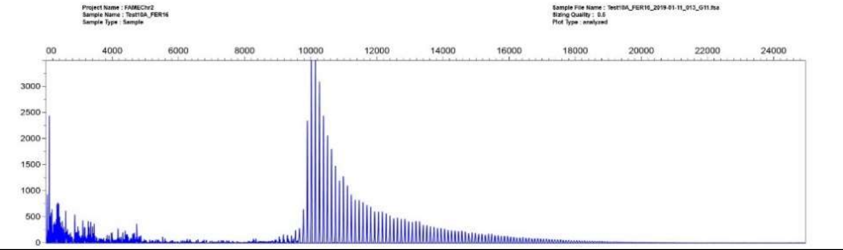
19	3	A	IV-1	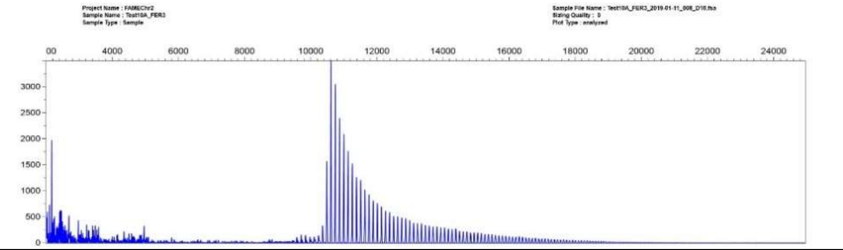
19	7	A	IV-3	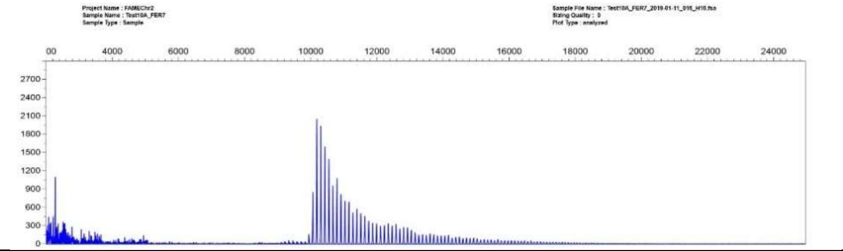
19	8	A	IV-4	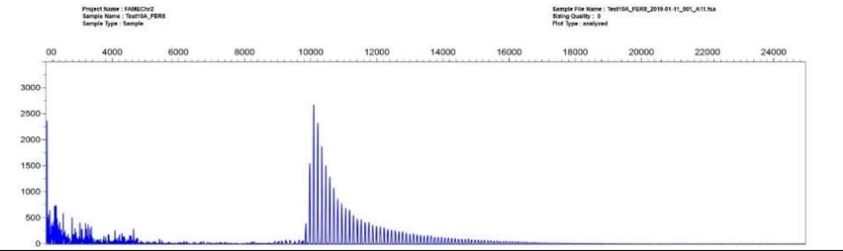
19	11	A	IV-5	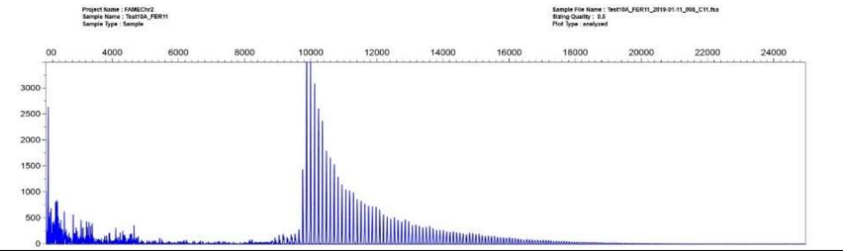
19	12	N	IV-6	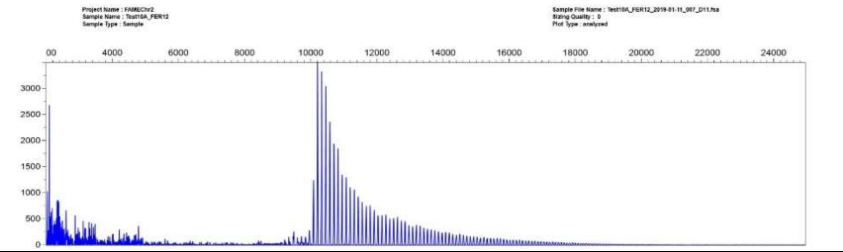

Family	DNA number	Disease Status	Pedigree Number	TTTCA RP-PCR trace
19	18	A	IV-7	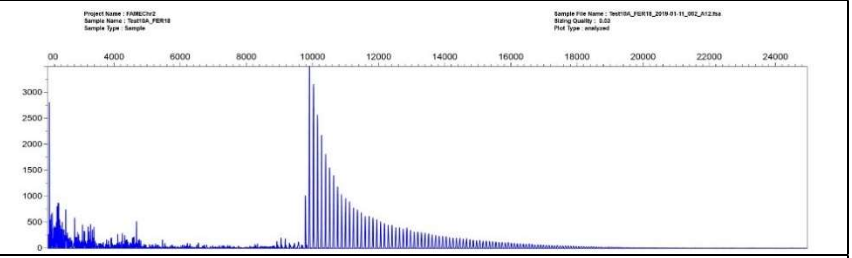
20	1	A	II-1	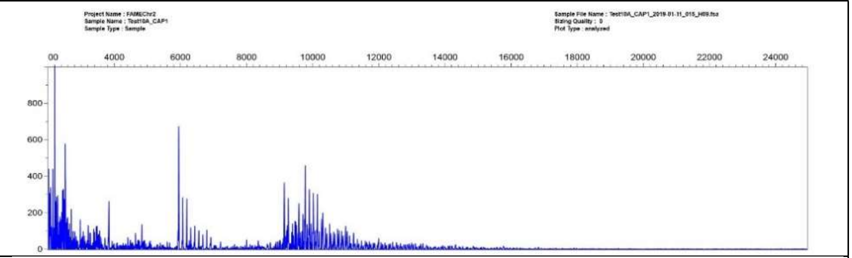
20	5	A	III-3	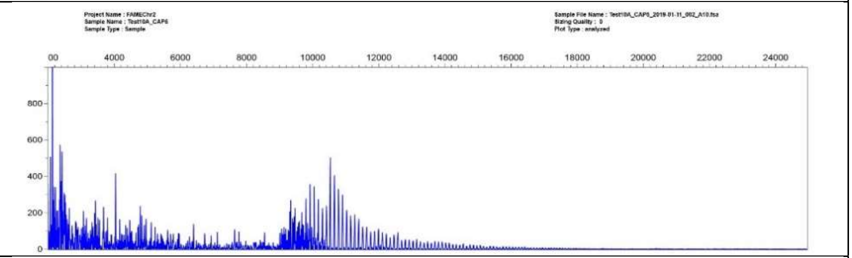
20	12	A	IV-4	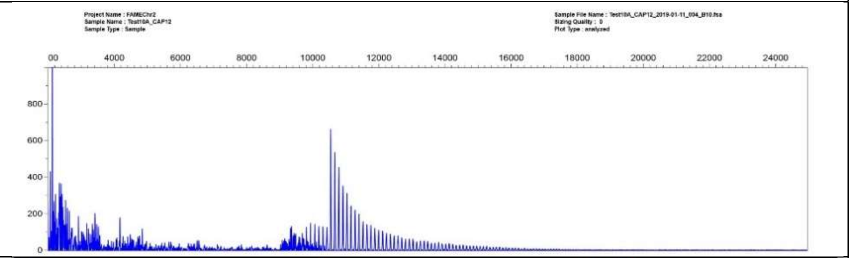
22	S1737	A	III-2	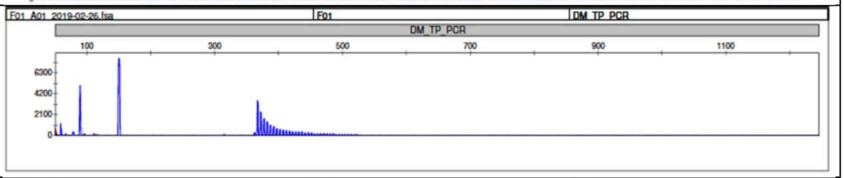
22	S1801	A	III-4	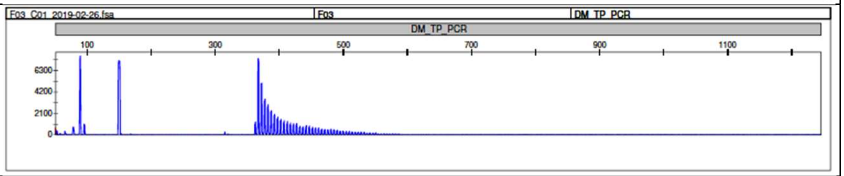
22	S1804	A	III-5	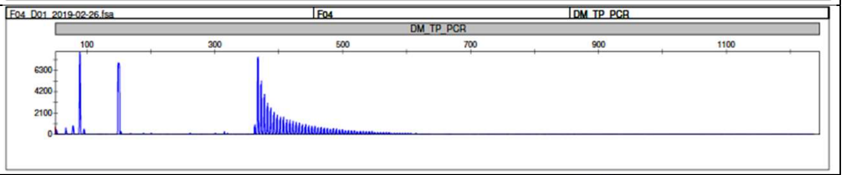
22	S1762	A	III-6	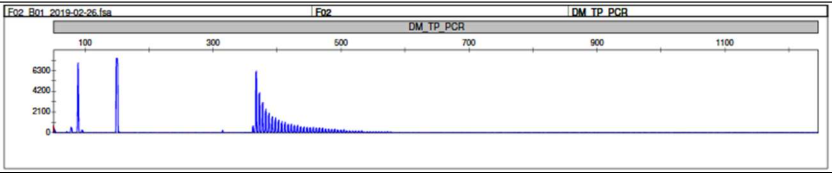

Family	DNA number	Disease Status	Pedigree Number	TTTCA RP-PCR trace
22	S1805	A	III-9	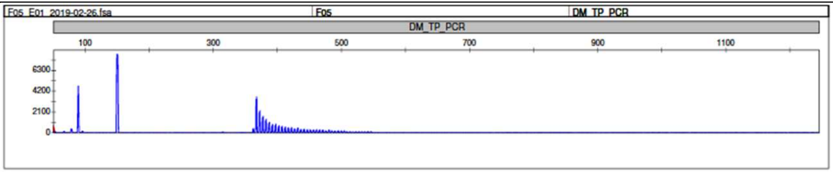
22	S1718	A	IV-4	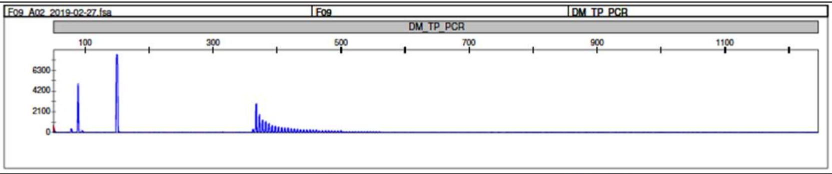
22	S1719	A	IV-5	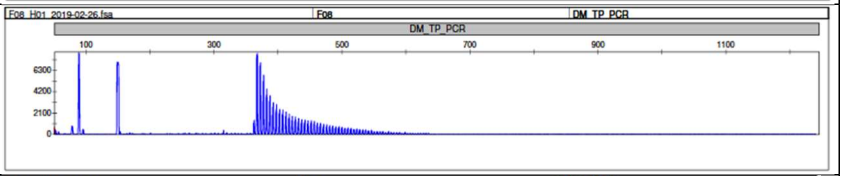
22	S1812	A	IV-7	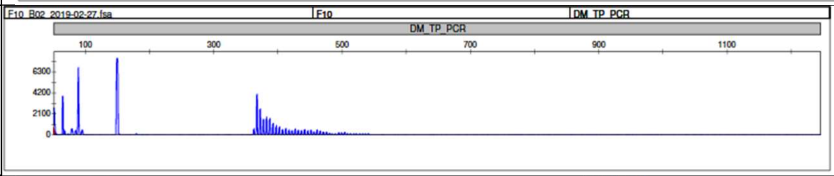
22	S1717	A	IV-8	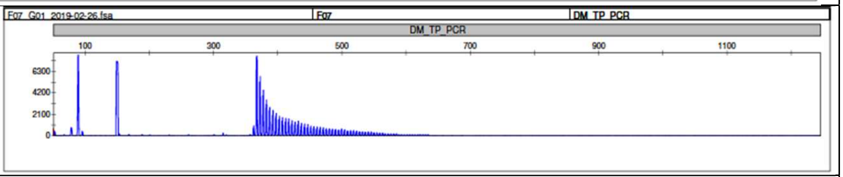
22	S1803	A	IV-10	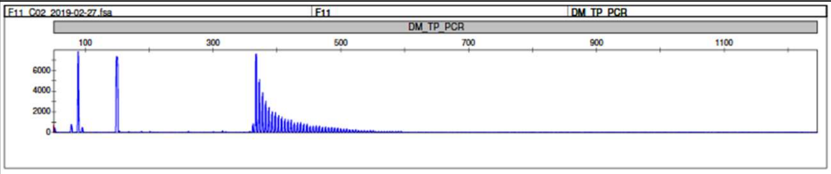
22	S1763	A	IV-13	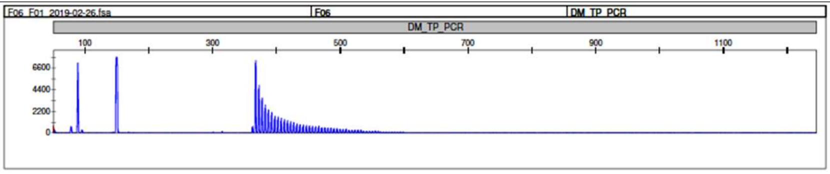
22	NMD347	A	V-1	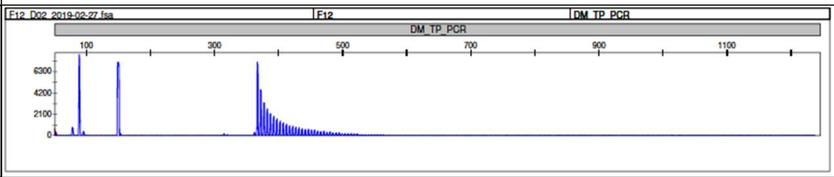
