# Supplementary material for: Intronic ATTTC repeat expansions in STARD7 in familial adult myoclonic epilepsy linked to chromosome 2
Source: Nat Commun. 2019 Oct 29;10:4920. doi: 10.1038/s41467-019-12671-y (PMC6820779; doi:10.1038/s41467-019-12671-y)
Supplement: Supplementary file 3 — Supplementary Data 2 [file 41467_2019_12671_MOESM3_ESM.pdf]

**#PacBio SMRT read from Family 1 IV-98**
>m54086_190222_190928/13828584/ccs
CCCTAAATCGAACACAAGCAATAGGGAGAAACAAAGCCATTATAAGGTGAAGTGGAACTAAA
ACCAGCAGCCACTAGCATCAATATCTATACTCTCAGTGGAGATTTAGCTTAAAAAGATAAAA
AATAAACACAAAACACCTCATTAGGCTGAAATAAATATTCTTTTCAAAGAAAGGTCACAGAA
TTTTAAAGAAGGCAGACAGCATGAGTCCAATCTTCCCATTACTCTGGCAAGGAAGCAGAAAT
CCAGAAAAGTTTCCATCCAGTGCTCTGCCCACTGTAGTAAAATGTTGACAGGAGGTACTCGC
TCTAGTTCCTGAAGAATGCTACTGATACAGGCTAATGCGTCAGTGGCTATGATTCTTTTAAC
TATCTCTCAGATAAGAGGGAACCAATGGATTGCCACTACTTTGTCCTTGGGGAACTTCCAGG
TACATCACCTACGTCAGGAGTTCCCAAAAAATGGTTCCCAGACCAGTAGCATCAGACTCCCC
CGAGCGAGAACTGTTCCTAGCGCCACACCCCCTCCTCCCTAGTTCAGGTAAGACATCCCTTG
AGAAAATTCCACAGCCATGACATGTTATCTCTTGGATGAGGTAAAACTCACCTCATCAAATG
ACTTGTGGGGACGGATAACCATTTGGGATTCATATGATCTGACCCTGACGAATTCTGGAGAC
TCTGGCACACTCGGATGCTCCACAGCACTGGTAAGGAGGAAAGGAGAAGCAAACTCTTAGTA
ATGTCTATATATAAAACAACAGGAGAGCTAAGTTAAGGGGAAGGCCTAAAAAGCTGGCTCTG
ATATTTTTAAATACACAAACAAAGGAGTCACTAAGATGAATAAATACAAACTAAGTTCTAAA
AATGAAGCGAAGGCCAGACGTGGTGGCTCATGCCTGTAATCCCAACACTGGGAAGCCAAGGA
AGGAAGATCACTTGAGGCCAGGAGTCCAAGACCAGCCTGGACCACACTGTGAAACCCTGCGT
CTACAACAAACAAATGAATTAATTAAAAATGAAGTGAGTTCCTTTGGTAAATTTACGGCTCA
CTAGGCTGTGGGTCAAAAACAACAGCACTTCTTTTGATTACCAACATTAAAGAACCCAAAAA
AACAGTTTAACTAAAAGCTTGTATCTCTCCATTCTTTCTAGACAGTCAGAGTCCCTCATCTT
CTAGCCTGGATGGAAGGGGCACTGGCTGCAGGGAAGCCTGTGGGAGACTCATAGGACACTAA
GAATATACAAACTCAGGTACGTAGGAATGAAGGCCACAGCTAAAGGAACTCGGCAACAGCCA
CAGGCAGCCATACATACCGCGACACCAACACCATCATGTTGTTTTCCTGATCCACACTATAC
CGCCGAACATAAACATAATCCCGTGAGTACATTGGATACTAAAGAAATGGAGGGGCAGGATT
AGTGTTCTGCATCACACAAACCTGGACAGAAATAAAATCAGAAAGTAAAAATACTCACAGGA
AAATGGGTTACCCAGTGAAGAACCTCGGAACCACTAACCACATCCCTCTCGATCACCTCCAG
CTTGATTACCAGGGCATCCCATTTTTTTCTATACTCTGTGTCCAGCTGCAGAAAGAGAAAAG
ACCATGAATACCCAAGAAGACCCAAAACAAAAATGCAGAAAGCTTTCCAGATGCTTTGGTCT
CTGATCCAAGAATCTTATTACAAGGAGAGAGTAATCTGACCTAACAGACAGTGCCAGAGTGT
GAGAGACATGGCTGTGTTTTACTACAGATGTTTCTTTCAAAATCACCTTATACAGGCTAATG
TCATTACACTGAATGTGAATCTTCATTAGACAAACCAAGGAAAAGTGTAACAAAATATTTAA
TAGACACATCTGGGGAAAATAATATATAGTGATATGAATGTGTAAGGGTAAGACAATTCTAA
AAAGTTTTAAACAACAAAAAGTCCAAAAGTATTAAGTAGACACTTCACACACAAAAATGCAA
GCAGCATATTTTTTTAAATTTCTGGCATCACTAGTAACTGAAGATTTTCAACTTAAGACAGA
TATACCATATACATAAAACCAACAAAAATTCACTACAAATTAACAAGATCCAGTACCTCAGG
GAAAGTGCACTTGCACGTTAGAGTGAGTGTTATAACCAACGAAGTAGTTATCGGAAGTCAGC
AAAACAAATGAAAAATCCAGAAAGAAAAAAATTTTGTTCTAGAACTCTGGGCGATCCCACCT
CCCTGTTTCATTTTTTAAACCATCTTCCCCTACAAGGAGGAAAGCAGTCTTTGAACAGATAC
TCTTCATCTTTATGAAATAAACATTGGGCCAACACAGTGACTCACACCTGTAATCCCGGCAC
TTTGGGAGGCAAAGGCAGGAGGATCACTAGAGCCCAGGGAGTTCAAGACCAGCCTAGGAGAC
ATAGGGAGACCCTGTCTCTAAGAAAAAAAAAATTTTAATTAGCCAAGTGTGGTGGCGCATGCC
TGTAGGCCTGGAAGCTGAGGTGGGAGGATCACTTGAGTCCAGGAAACAAAGGCTGCAGCAAG
ACATGATCACACCACTGTACTCCAGCCTGGGTAACACAGCAGGACCCTGTCTCAGGGAGAGG
AAAGAAAAAAAAAAAACCTCCACTATGGAAATGTTCTTGATTTGAGGTTAAACCAGATGTGAC
AACTAAATATAATCCTATCTGTACTGAAGTGGGAAAATGCTATAAAGGACATTGAGTCAACT
GATAAAACTGATAAATACAAACAGAGGACTACATGAAAGTGTTCCATCAGTGTTAAATTTAC
TAATTTCAAATAATGGTATCATGATTAGTTAAGAAATACGGCATATGGCTAGTTTTAGGAAA
TACACACTGGAAGAATTTAGGAGTAAAGGACTATGATGTATGTAACTTACTCTTAAGTTGT
TTGGGGAAAAAAACAATAAAGCCTCATAAAATACATTAAAGTATTCATATCCATTCCTCTAT

CTGATATTCCCAACAGTCCTGTGGGAGGGTAAAACTGATCTCATCCCCATGGGACAGATAAA
GAAATTCTAAGTCAAATGTTTACCTAAATGCTATGCAATTGTGGATGACAGGTAGATAGATG
GATGTACTTAAGCCAATGTCACCTGAGTCAGATAAATGATTCATGTAGACAGTAGCAATTGA
TATGCTAGGAGGCTCCAAATCTGGAGAGAAAAATTAACTCACCTGAACATTGAAGAACTGCC
GAGGTGTCACATCTGTGTAGGTTCCAAAAACTAGAATGAAAAGAAAGAATAAGGGATGCTGG
CCATACTCCAGTCACTCGCCTTGTTTCTTTCACCTAGCGGCGTTTCAGATCTAAAGTACTAA
GAGATCTTAAAGGCAGGTTCAGAAACTAGAAGTCTTAGTAGAGCAACAAAGAAAGACAAAGA
GGATGTAAGCAAAGACAACACTGAGACATTTGTCATCTGGGAGAAGAAAAACAATAAACAAA
AAACTACGGCAAATTTTCTGAGAAACAACCAGAACAGTTTAGAGAAGTATGAAGAACAGAAA
TCCCATGCACCTGTGCAAACTATGGAACCCAAAAGATAGCCACACTGGGCTCACCTCGGTAC
TGGTAAAGGTGGGTGCCTGTAATTGGGCGCCGCCACAGCTTAAAGTGTTTCTTATCCATCAC
CATTTCCCAAGGTTGCTCTTTGCCCTCTGAATCTTCATTCCCTTCTGTTTGGGCTTTTGGTT
CTGGAGGGTGGTGCTGGACTCCAGAGCTCTGAAACATATTTGACATTTCTTCCAACCGCTTC
ATCTCATTAATAGATCTGTAAAGGGGAAAAGAGCCATGGTGAGGTGGTTAGTCAGCCCTGTG
AAATGAAGTGTCCACACAAAGGTCTGTGCAAGAAGGTTATACAGTAAACCACAGTATGTAGT
ATATAAACAGGACACAGTATAGTCAATAAAAACTGAGTTCCTGAGTTTGAATCTTAGTCCAA
CACTCACTAACTAGATTATTATTTAACCAGTTCCTATTTCTAAATCTATAAAATGGAAATAA
TAATAGTACCTATCTCGAGGTTGGCTATAAAGATTACAAAGTGTCAAGTGCTGCAGGGCGTG
GTGGCTCACGACTGTAATCTCAACACTGGGAGGCAGAAGAGGGTGGATCACGAGGTCAGGAG
CTCAAAACCAGCCTGATCAACATGGTGAAACCCTGTCTCTACTACAAACACAAAAATTAACC
GGGCATGGTGGCGCGCAACTGTAATCCCAGCTACTCAGGAGGCTGAGGCAGGAGAATGACTT
GAACCTGGGAGGCAGAGGTTGCAGTAAGCCGAGATCACGCCACTGCACTCTAGCCTGGGCGA
CAGAGTGAGACTCTTGTCTCAAAAAAAAAAAAAAAACAAAAAACAAAAACAACGAAAAAAAAG
TAAGTGTCAAGTGCTTAATATCTATGGCCAGGCACAGAGGCTCACTCCTGTAGTCCCAGCAC
TTTGGGAGGCCAAGGCAGGAGGACTCCTTGAGGCCAGGTTCAAGACTAACCTGGGAAACACA
GTGAGACCCTACCTCTAAGAAAAAAATTTAAAAAATTAGCCAGGCATAGTGGCCACATGCCT
GTACTCCAAGCTATAGGCTGAGATGGGAACATCACTTGAGCCCAGGAGTTGGAGGCTGTAGT
GAGCTACAATTCTGCCACTGTACTCCAGTCTGGGCCATACAGTAAGACTCTGTCTCTTTTTT
TTGTTTGTTTTTTTGAAACAGAGTCTCACTCTGTCACCCAGGCTAGAATAGAGTGCCATGAT
CTGGGCTCACTGCAACTTCCGCCTCCTGGTTCAAGTATTTCTCCTGCCTCAGCCTCCCCAGT
AGCTGGGACTACAGGCACGCGCTATCATGCCCGGCTGATTTTTTGTATTTTTAGCAGAGACA
GGGTTTCACCGTGTTACCCAGGACGGTCTCAATCTCCTGACCTCATGATCTGCCCGCCTCGG
CCTCCCAAAGTTCTGGGATTACAGGCGTAAACCACCGTGCCCAGCTACACTGTCTCTTAAAA
AATAAAATAGGCTAGGCGCAGTGGCTCACGCCTGTAATCCCAATACTTTGGGAGGCTGAGGC
AGGCGATCACCTGAGGTCAGGAGTTTGAGACCAGCCTAACCAACGTGGAGAAACCCTGTCTC
TACTAAAAATACAAAATTAGCTGGGCACGGTGGCACATACCTGTAATCCCAGCTACTCGGGA
GACTGAGGCAAGAGAATCGCTTGAACACGGGAGGCGGAGGTTGCAGGGAGCCAAGATCGTGC
CATTGCACTCTAGCCTGGGCAACAAGAGCGAAACTCCGTCTTATTTATGAAATGAAATGAAA
TGAAATGAAATGAAATGAAATGAAATGAAATGAAATGAATGAAATGAAATGAAATGAAATGA
AATGAAATGAATATGAAATGAAATGAAATGAAATGAATGAAAATGAAATGAAATGAAATGAA
ATGAAATGAAATGAATGAAATGAAATGAAATGAAATGAAATGAAATGAAATGAAATGAAATG
AAAATGAAATGAAATGAATGAAATGAAATGAAATGAAATGAAATGAAATGAAATGAAATGAA
ATGAAATGAAATGAAATGAAATGAAATGAAATGAAATGAAATGAAATGAAATGAAATGAATG
AAATGAAATGAAATGAAATGAAATGAAATGAAATGAAATGAAATGAAATGAAATGAAATGAA
ATGAAATGAAATGAAATGAAATGAAATGAAATGAAATGAAATGAAATGAAATGAAATGAAAT
GAAATGAAATGAAATGAAATGAAATGAAATGAAATGAAATGAAATGAAATGAAATGAAATGA
AATGAAATGAAATGAAATGAAATGAAATGAATGAAATGAAATGAATGAAATGAAATGAAATG
AAATGAAATGAAATGAAATGAAATGAAATGAAATGAAATGAAATGAAATGAAATGAAATGAA
ATGAAATGAAATGAAATGAAATGAAATGAAATGAAATGAAATGAAATGAAATGAAATGAAAT
GAAATGAAATGAAATGAAATGAAATGAAATGAAATGAAATGAAATGAAATGAAATGAAATGA
AATGAAATGAAATGAAATGAAATGAAATGAAATGAAATGAATGAAATGAAATGAAATGAAAT

GAAATGAAATGAAATGAAATGAAATGAAATGAATGAAATGAAATGAAATGAAATGAAATGAA
ATGAAATGAAATGAAATGAAATGAAATGAAATGAAATGAAATGAAATGAAATGAAATGAAAT
GAAATGAAATGAATGAAATGAAATGAAATGAAATGAAATGAAATGAAATGAAATGAAATGAA
ATGAAATGAAATGAAATGAAATGAAATGAAATGAAATGAAATGAAATGAAATGAAATGAAAT
GAAATGAAATGAAATGAAATGAAATGAAATGAAATGAAATGAAATGAAATGAAATGAAATGA
AATGAAATGAAATGAAATGAAATGAAATGAAATGAAATGAAATGAAATGAAATGAAATGAAA
TGAAATGAAATGAAATGAAATGAAATGAAATGAAATGAATGAAATGAAATGAAATGAAATGA
AATGAATGAAATGAAATGAAATGAAATGAAATGAAATGAAATGAAATGAAATGAAATGAAAT
GAAATGAATGAAATGAAATGAAATGAAATTGAAATGAATGAAATGAAATGAAATAAAATAAA
ATAAAATAAAATAAAATAAAATAAAAATAAAATAAAATAAAATAAAATAAAATAAAATAAAA
TAAAATAAAATAAAATAAAATAAAATAAAATAAATAAAATAAAATAAAATAAAATAAAATAA
ATAAAATAAAATAAAATAAATAAAATAAAATAAAATAAAATAAAATAAAATAAAATAAAATA
AAATAAAATAAAATAAAATAAAATAAAATAAAATAAAATAAATAAAATAAAATAAAATAAAA
TAAAATAAAATAAAATAAAATAAAATAAAATAAAAATAAAATAAAATAAAATAAAATAAAAT
AAAATAAAATAAAATAAAATAAAATAAAATAAAATAAAATAAAATAAAATAAAATAAATAAA
TAAAATAAAATAAAATAAAATAAAATAAAATAAAATAAAATAAAATAAAATAAAATAAAATA
AAATAAAATAAAATAAAATAAAATAAAATAAAATAAAATAAAATAAAATAAAATAAAATAAA
ATAAAATAAAATAAAATAAAATAAAATAAAATAAAATAAAATAAAATAAAATAAATAAAAAT
AAAATAAAATAAAATAAAATAAAAATAAAATAAATAAAATAAAATAAAATAAAATAAAATAA
AATAAAATAAAATAAAATAAAATAAAATAAAATAAATAAAATAAAATAAAATAAAATAAAAT
AAAATAAAAATAAATAAATAAAATAAAATAAAATAAAATAAAATAAAATAAAATAAAATAAA
ATAAAATAAAATAAAATAAAATTAAAATAAAATAAAATAAAATAAATAAAATAAAATAAAAT
AAATAAAATAAAATAAAATAAAATAAAATAAAATAAAATAAAATAAAATAAAATAAATAAAA
TAAAATAAAATAAAATAAAATAAAATAAAATAAATAAAATAAAATAAAATAAAATAAAATAA
AATAAAATAAAATAAAATAAAATAAAATAAAAATAAAATAAAATAAAATAAAATAAAATAAA
ATAAAATAAAATAAAATAAAATAAAATAAAATAAAATAAAATAAAATAAAATAAAATAAAAT
AAAATAAAATAAATAAAATAAAATAAAATAAAATAAAATAAAATAAAATAAATAAAATAAAA
TAAAATAAAATAAAATAAAATAAAAATAAAATAAAATAAAATAAAATAAAAATAAAATAAAA
TAAAATAAAATAAAATAAAAAATAAAATAAAATAAAATAAATAAAATAAAATAAAATAAAAT
AAAATAAAATAAAATAAAATAAAATAAAATAAAATAAAATAAAATAAATAAAATAAAATAAA
ATAAAATAAAATAAAATAAATAAAATAAATAAAATAAAATAAAATAAAATAAAATAAAATAA
ATAAAATAAAATAAAATAAATAAATAAAATAAAATAAAATAAAATAAAATAAATAAAATAAA
ATAAAATAAAATAAAATAAATAAAATAAAATAAAATAAATAAAATAAATAAATAAAATAAAA
TAAAATAAAATAAAATAAAATAAAATAAAATAAAATAAAATAAAATAAAATAAAATAAAATA
AATAAAATAAAATAAAATAAAATAAATAAATAAAATAAAATAAAATAAAATAAAATAAAATA
AATAAAATAAAATAAAATAAAATAAAATAAAATAAAATAAAATAAAATAAAATAAATAAAAT
AAAATAAAAATAAAATAAAATAAAATAAAATAAAATAAATAAAATAAAATAAAATAAAATAA
AATAAAATAAATAAAATAAAATAAAATAAAATAAAATAAAAATAAAATAAATAAAATAAAAT
AAAATAAAATAAAATAAAATAAAATAAAATAAAATAAAATAAAATAAAATAAAATAAAATAA
AATAAATAAAATAAAATAAAATAAATAAAATAAATAAAATAAAATAAAATAAAATAAAATAA
AATAAAATAAAGCCAAGCACAACGGCCTCACGCTGTAATCCCAGACTTTGGGAGGCCGAGGT
GGGCGGATCACCTGATGTCAGGAGTTCGAGACCGGCCTGGGAAACATGGTGAAACTCCATCT
CTACTAAAAATACAAAAATTAGCTGGGCATGGTGGTGGACGCCTGTAATCCCAGCTACTCGG
GAGGCTGAGGCAGAAGAATCACTTGAACCCAGGAGGCTGAGGGTGCAGTGAGCCAAGATCGC
ACCATTGCACTCCAGCCTGGGCAACAGAGTGAGACTCCATCTCTAAAATAAATAAAAAATAA
AAATAAAATAAAGTGTTTGTTATCTGGCACGTAAGTAGCCCTCAAATGTTAACTATGAAGGT
TTATGGTTTAAAATGTCAATTGATATCTTTTGTCCGTTTTTATTGGGTTGTCTTATTATAAG
TATGTATGTATTTTTCCCCTTTGTTTTTAAATTGATAAATTCACATACCATAAGATTCATTC
TTTTACAGTGTACAATTCTGTACTTGTTAGTATATTCACAAGGTTGTGCAACCATCACCCCT
AATTCCAGAACATTTTTATCACCCCAAAAGAAACCTCTATCTATCATTAGTCACTTCCCATT
CCTGCCTCCTCCCAGGTCCTAGGCAGCCACTAATCTACTTTCTGTCTCTATGGAGTTGCCAA

TTCTGGACATTTCAAGTAAATGGAATCATATGATATGTAGCTTTTGTGTTGAGCTTCTTTCA
CTTAGCATACTTTCAAGTTCAACCATGCTGTACTTTATTCCTTTTTATAGCTGAATAATATT
CCATTGTATGGATAGATCCCATTTTGTTTATCCATTCATCAAATAAAATTTGTTTCCACTAT
TTTAATTATAGTGCTATTATAATAGCACTATATTATATTACTGCTATTATAATACTGCTGGC
CGGACGTAGTGACTCATGCCTATAATCCTAGCACTTCGGGAGGCCAAGGTGGGTGGATCACT
CTGAGGTTGAGTTCGAGACCCAGCCTGGCCAACATGGCAAAACCCCATTTCTACTAAAAATA
CAAAAATTAGCCAGGTGTGATGGCAGGAGCCTGGAATCCCAACTACTTGGGAGGCTGAGACA
GGAGAATTGCTTGAACCCAGGAGGTGGAGGTTGCAGTGAGCCAAGATTGCACCACTTCACTC
CAGCCTGGGCGAAAGACAAAACTGTCTCAAAAAAAAAAAAAAGAATACTGCTATGGGACATT
TGTGTACAAATCTTCATATAGACATGTTTTCAATTCTCTTGGTAGATTCCTAGGAGGCAGAT
TTGATGGATCATGGTAACTATGCTTAACTTTTTAAGCAACTGCCAAAATGTTTTCCAAACAA
TTACACTATTTTATATTCACATCAGCAATGTTTGAGGATTCTAACTGCTCCACATCCTTGGT
TCCAATTGCCCCACTTGTTATTGTCCATCTTTGTTATTCCAGCCAACTTAGTGGGTGTGAAA
TGGTATCTCATTGCGTCTTGACTGGTATTTCCCTAACCTTTTCATGTGCTTATACTAGTCTT
GATAGTATCCTTCATGCCATAAAAAGTTTTTAATTTTGATGTTTATTTTTTCTTTGATTGCT
TGTTTTTGGTGTTTATAGCCAAGAACCACTGCCTAATCCAAGGTCATAAGAACTTATGCCTA
TATTTTCTTCTAAGAGTTTTTAGAATTTTAGCTTACATTAGGTCTTTACCCCATTTCAAGTT
AATGTTTGTGTATGATAATGAGGTAGGAGTCTAAACTCATTCTTTTGCATCTGCTTCTCCAG
GTGACCCAGTGTCATTTGTCGAAAAGCCTATTCCTTCCCCAGTA

**#Oxford Nanopore read from Family 1 IV-98**
>57c288c8-ae09-4030-b991-3e8e01bd1b03
runid=250c0471202951dfd47a182eee143db3c1b23211
sampleid=LLAAB034003 read=2270 ch=85 start_time=2018-03-
21T22:01:23Z
GTTGTACTTCGTTCGGTTACGTATTATTGCTTGGCATAGAAGAAGAAAAACATAGGAGAAAA
TATTTATATCTTAGACAGACAAAGTTTTCTTAGAAACACAAAAAAGCACAGGCAGGGTGCAG
TGGCTTACACCTGTAATCCCAGCACTTTGGGAGGCCGAGGTGGGCAGATCATGAGATCAGGA
GATCGAGACCATCTGGCCAACATGGTCGAAACCCTGTCTCTACTAAAAATATAAAAAATTAG
CTGGGTGTGAGTGGCATGTGCCTGTAATCCCAGCTACTCAGGAAAACTGAGGCACGAGAATC
ATTTGAACCCAGGAGGTGGAGGTTGGTAGAGCTGAAGATTTGCACTGCACTCCAGCCTGGCA
ACAGAGCGGAGGCTCATCTCAAAAAAAGAAAGAAAAAAAATCACACACACAAAATACAAACAA
TTGATAAAGCTGAACTTTGTCCAAAAATTAAAACCTTTTTACTCTTTGAAAGACACCTTAAA
AAACAGGGCCTGCTGGATGCAGTGACTCACACCTATAATCCAACACTTTGGGAGGCCAGGCG
ACGAATCAGGGTCCAGAAGACCAGACTGGCCAACACAGGTGAAGCCCCATCTCTACTAAAAA
TACAAGTGTAATCATGGTGTAGATTTTACGCCTGTAGTCCCAGCTACTCAGGGAGGCTGAGG
CAGGACAATCCTCTTGAACCTGGGAGGCAGAGGTTTGGCGGGTATGGCCGAGATCTATTGCC
ACTGCACTCCAGCCTGGGCACGCGGGATCGAGACTCCATCTCATGCAAACAAAACAAAACAA
CAACAACAAAAAGGCAAGCCACAGACAGGGAAGAAAAATATTATAACACATATATCAGACAA
ATGAGTACACATCTACACATCTAAAATATATAAATACACAACTCAACAATAAAATGACAAAT
CGATTTTTAACTGAGTGACGGTTTGTCAACCATTCCCTCCAAAAGAAGATATGCAAATGGCA
ATAAGCACATGAAAAAGATGCTCAACGTCATTGAATATTTAGAGAAATGCAATTCAAAACCA
CAATGGGAGATAACTTATTTCACACACATATGGGGATGGCTGAATAAGAACAACAAATGTTG
TTGGAGGATGTGAAGAAGATTGAAACCCTCACAAACTGCTGGTGTGAAGCGTGAAATGGTGT
AGCTGCTTTGGAAAATAGTTTGAGTGATTCCTCAAAAAGTTAAACATCCATTAACCATGTTA
CAGGTAGTTAGACAGGCGTGAGCAGGAAGAAGGGACTCTCTCAGCCCACTAAGTGTTGGTTA
TTGGTTGACAGTTATTGCATTGCCTCTCTAACGGTGATAAATTGGCAGCTGGTGCCAGGAGA
GCTATTTCTGATGGACCCACACCTGTTGCTAACGTATTAATTAATGGGTGCCAGGGAGAAGT
AACTTCCAGGCATAGCCATTAAGAACAAAATGATGGACGCCTGGGGGCACCCACCAGAAAAG
GGAAGAAAGCCTCAGATGGGCATACACACAGCACCCTCAACCACTACCCTAGCTCACTTCCC
AAGGGTAGGGGCACTGCGCATGCGAAGCCACCTGAGAAGAATCATGGAAAGGGGCACAAGAC

AGCCATAAGGTGGGCCAGCTATAAAGTCCTAGGATCGGGTTAAACATCACACTTGTTCTTTA
GGGTTCAGCTTAAGGCCTCTTCCAAGCTTTAACTTTCCCTTCTCTTTCCCTGTTTTAAAGGC
TTCCTAGAATAAACTTCCATTTCCTGCTCCTGAGAATTGCCTTGATCTCTTTTTCTGCCCTC
AGTCGAATTATTTCTTCTGAGGAGGCAAGAGTGTGAGGTTGCTGCAGACCCTTACAGATTTG
CCAGTGCTCTGATACCTCACCAGTAATAACCATATGACCTTAGCAATCCCACTCCTACTTAT
ACCCAAAGAAGAGGTAAACATATGTCTACAGAAGTGTCACACGAGGTGTTCATCAATGGTAT
TATTCATAATACTTAAGAAGTGAAAAACAACCTTTAAATGCCTATATGTGAATGAATGGACA
AAAATGTGAACAATGTATACAACAGAATACGCCCGTGTATTCAGCCCAGTGGCTCACGCCTA
TAATACCAATATTTGGGAAGCTGAAGCAGACAGATAACTTGAGGCCAGGAGTTCAAGACCAG
CCTAAAGGAACATGGGTGAGCTTCTCAGCTACTAAAAATACAAAAATTAGCCAGGCGTGGTG
GGTGCATGCCTGTAATCCCAGCTACTGGAGGCTGAGGCATAAGAATCACTTGAACCCAAGGC
AGAGGTTACTTGAGTGGAAAGCCAAGACTGTTGCCGCTGTACTCAGCCTGGATGACACAGCG
GAACTCTATCAAAAAAAAGTATTCATATAAGGGAATGAAGCATGTATCAGTAGATACATGCT
ATAATATGGATGAACCCTGAAAATCTAAAAATGAAAGAATCAGTCACAAGAGATATACATCA
TTATGACTCTTTCCTGTATAGAGAAATGTCCCAAATGAGCAAATTCATAGAGACAGACGGTA
GATTAGTGGCTGCCAGGGGCTGGGGAGAAGGTAAAGAAATTGTGGTTTATAATGAATCTGAA
TTTTTCACAAGGGGTAATGAAAATGTTTTGGAAATTAGGTCATGGTGATGGTTTATAACCGT
ATAAATGTGCTTAAAACCACTGGTGTATATATCTTAATTTTTGGAAAAAATAGAAATACTCG
TGTCAAGACAAACAACCCAATACAAAATGAGCAAAGATTTCCAAATGACACTTCACAGGAGG
AAGAAGTTTTATGACCACCAATAACCACATAAAAAGATGCTAAACATCATTAATCACTGAGG
AGATGCAAATTAAAGCCATAATGAGAGAAGACAGATCACAAAACATTTTGGCAAGGAGATGG
AACGAAAACTAGAAACTCTTATACACTGCTCACTTAGGAATATAGACAGGCAACCACTTTGG
AAAACAGTTTGGCAGTTACTTTAAAATATTTGAGGCCGGGCACAGTGGCTTGTGCACCTTTA
ATCCCAGCACTTTGGGAGGCCAGGCGGGTAGATCACCTGAGGTCAGGGGTTCAAGACCAGCC
TGGCCCAGATGGTGAAAACCCCATCTCTACTAAAAAAATCACGAATTAGCGGGGTGTGTTGG
TTGCATGCCATGTAATCCCAGCTACTTGGAGGTTGAAGGCAGGAGAATCACTTGAACCCAGA
GGCGGAGGTTGCAGTGTCGACCGAGATCAGCCACTGCTACAGCCTAGGCAACCTGGCGAGAC
CTGATCTCTAAGCTAAATAATAAATAAATATTTGAACCTTACTTTTACCATGACTCAGGCAT
TTCATTCCTCAGTATTTGCATAAAAACCTAAATATGTTACACAAGACTTGGCCTGAGAAATG
TTTTACAGCAGCTCTGCTCTACAATCCTCCCAAACTGGTAACAACTCCGTCATTCCTCCAAC
AGGTACATGAGTGAACAAATTATTAATTTTTGTCAAATTTCAATTGGAATAATACTCCCAAG
CCATGGGGGGTGAACTACTGATAAACACAACATGAGCGATCATAGAATCATTATGCTGAATT
CAGAAATGCATAAGCCACACTACAACACACTCTAAAATTCTATTTATATAAAACTCTATAAA
ATGCAGACTATTCTATGGTGGGTATGCTCAGCCCAGCAAGGGCAGGGGAAGACTTGCAAGGG
GTATAAAAGGGAAACTTTTGTGGGTGATGAAATGTTCATTATCTTCTAGTATTGATGATGAC
ATCACAGGTATATCACATGTGAAAATTTCTCGGGTTGTTGCATTTATTTAAATGGAATTTAA
GTAAAAAATTTATTAGATTTGTGCATTTTAAGTACATACGGCTTACTGTACTTTGATTATAC
TCAAACAAAGTCAATGAATAAAAACTACTTAATAAAAGAGTGGGGACCTGAGTACAAAGATG
ACGGTGGACACCCCCTGAACCCAATATACACGTGACAACAAAGAAGGGGTGAAGCAAAGGCA
AACCCCACAATCAAGGAGAAGAAACAGAAACATTTTTGGAAGATTGGAGAGGAAGGTATATG
GATCCAAATGTGATAAAGTAGTCAACTGATTCTAACCAGTGGTGAGGAATGATTGAAACAAT
CCAGTTTGCAGGTGGATACCCAAAAGGCTCAGGCTCTGGTGAGGTACCATGTTGGGTGAAGA
GAAGGGATAGACTAAGAACCAGCTGAAGGTTTGGTAACAATAGCCAGCCTACCCTATAATGA
GGTCATGGCGACCACATCTCACATGCTGAGAACCCCGTCTGAGAAATTCTGTAGAACCAACA
ATAACAGCCAAATATCTCAATCACAAGAGGCCACTAAGGAAAAAAAGTAACTTGGAAACATC
GTGGAAAGCATAAAAATCACTCCCTCTAACATCATCCTCAGGGAAGATACTGTGCCACCATA
TAAGCGCAGAACAGTTGCCACAAAGAAGAGCAAACACTCAGAAAATAGGTCTCCAGTTTATT
AAAAATAAGAAAGCAGAATTTAAAACTCAATATCAGGCTGGAGGTGGTGAAAACCTGTAATC
CCAGCACTTTGGGAGGCCGGGTGGGTGGATCACTTGAGGTGGGTTCAAGACCAGCCTAGCAA
GCCCTATATAGTGAGAAACCCGTCTAAAACACAAAAAAATATGGCCAGGTGCAATTGGCTCA
CGCCTGTAATCCTAGCACTTTTTGGAGGCCGGGGCGGGCGGATCACGAGGTCAGGAGATCGA

GACCATCACAGCTAACACGGTGAAACCTCTCTACTAAAAAAATAAAAAATTAGCCAGGTGTG
GTGGCAGGCTCCTGTAGTCCGCTACTCGGGAGGCTGAAGGCAGAGATAAGCATGAAGCCTGG
GAGGCGGAGCTTTGCTGCTTGAGCGAGATCACGGAAACTGCACTCAAGCCTGGGCAACAGCG
ACAAAGACTCCGTCTCAAAACTTACAAGTGTGAGCGCGCCTGTAATCCCAACCAACTCGGGA
GGCTGAGACAGGAGAATCGCTTGAACCTGGAGCCCAGACCCGCGAGGAATTGAAATCTTACC
ACACCTGCTGCAGCAGCAGAGATCCCAATTGCCAGAGAGGGTGCCAGAGTGGTTTTGTTTAT
TTTGTTTTGTTTTTGTTTTGTTTTTGTTTGTTTTGTTTTGAGACAGACCTTGCTCCGTCACC
AGGCTGGAGTACGAGTGGCTGTCTGGCTCACTGCAACCTCCCGGACTGGCGATTCTCTGCCT
CAGCCTCGAACTGGGACTACAGGCGTGCGCACCACCACCACACCCAGCTGGACACTGTATTG
TTGAGTGGAGATAGGGTCTCGCGATGTTACTAGGCTGATATCAACTCCTGGTCTCAGTGCTC
CACCTATCTAGGCCTCCCAACTGCTGGGATTACAGGCGTGAGCCACCCGCGCCAGCCGAGGG
TGGTTGAGTGGGTAAGGACACAACAGGGCCAGGCAGAGTAGGTGCCTGTAGTCCCAGATACT
CAGGGGTGAGGCAGGAGAATCGCTTGAATGCGGGAAGCAGAGGTTGCAGTGAGCCGAGATCG
AGCCATGCACTCCAACCTGGGAGACAGAGGTGAGACTCCATCTCAAACACACACACACACAC
ACACACACACTCCACCCAAGCCCATAAGACAGGGGTGAGACCCCATCTCAAAAGCAAACATA
CACACACACAACCACAACCCTGGGAGACAGGTGAGACTCCATCTCAAAGCACACACACAGAG
AGAGAGAGAGAGAGAGAAAGAACCCTGGGAGACAGGTGAGACTCCATCTCAAAACAAACACA
CACACACAAAACCCTGGGAGGTGAGACTTGAATATAAAAACAAAAAAACACACACACACAAC
CACAATCACCAGAGACAGAACCATCTCCAAAAACAAAAAACACACACATACACAACCACAAC
AAACTAAATAAATGAAAAACCAAAAGTACTGGCTCTAGGAAAACAAAGACATGATTTGCTGA
AAACAGTGTAGTGTCTTGCCTGTGATATTTCCATACTCGTATGCTGTAAATGCTAAATAATA
CTGGCAACTAATTCAGGTATAATTAGAGTACTCTGGGACAAAGGGGATAAGGGGAGACAGGA
GGGGAAGGAAGAGCTAAGTCATTGCGAGGGAGGTCAAGGATGGGAAGAAAAAAGCAAATCTT
CATATAATACTTTAAAATGACTTTTCGAAAATGAGAATAAACACATTATTTAGAGACCTAGA
GATAAATATCAAATATAATAACAGGGAGAGGGATTTAGAAACTGGCTGTCCCTGAGAGAGGG
GAGATGGTGGAGGTTTTCACCAATAAACTTTAAGAACGATTTGACTTTTTTTTTTTTTTGGA
GCTGAGGTCTCTTGTCTGGGTTCAATGGTGCCATCTTGGCTCTGCAACCTCCGCCTCCCTGA
TTCAAAACCTCCGGAGTAGCTGGGGTTACAGGTGGCATAAGCCATAGTACTTCGACCGCAAA
CTTTTCCAACTATCTTTAAATATCTTTGGATCATCCATGGTTTTTAGTTTTTGGGTTTTAGA
TGGAGTCTTGCTGTATTTGCCCAGGCTGTAGGCCGGCAGTGCGACCTCAGCTCACCTGCAAT
CTCTTCCTCCCGGGTTCAGCCGATTCTCTCGTGCCTCAGCCTCAGTAGCTGGGATTACAGGC
GTGTGTGCCACCACGCCCAGTTTAATTTTGTATTTTTAGTAGAGAATACTAAGAGTGCTGAG
ATTACAGGCGTGAACAACGATGCTCAGCCGAAAAATGTGCAGTACAAATACCAAATTGGAGA
GAAAAATACACTGATGCTCTGGGTTTGGTACAGATTGCAATAATTTTTTTTCTTTATATTTT
TATGTAAAAATACTTAAGTTAGGCCAGGCGCAATGGCTCATCACCTGTAATCCCGGAAACTT
TTTGGGAGGCCAAAGAGGTGGGTGGATCACCTGAGGTCAGGGTTCAAGACCAGCCTGGCGGC
ATGGGCCAAAATGTCTCTACTAAAATAAGAAGTGCGGCGTGGTGGTGTGCTACAGTCAGCTA
CTGGGAGGCTGAGGCAATGAACCTGAAATAGTGAATTGCAGTGAGCCACTCTGCCTCCAGCC
TGGGTGATAGAGTGAGACTCCGTCTCAAAAAAAAAAAAGAAATGCATAGAGTGCATTATCTTT
TAGCCTCTTAATTAATACTGAAAACAAAAAGAATATGAAGGCAAGTCAAAAGGGTATCCAGA
ACATTCTGGGTGATGAACCAACAGGCCGCTGGTGTAACTAATTCGAGACAGTTGGTCTCAGG
AACAGGTCACAAGCTCACCCCAGTCCTACTCTGTCCTTACTACACAGGACTGAGGCTTTGCG
TAGTTCTCGATGCTGGGCTGTGCAATTATCATCACAAACCCAGGGTTGTCCCCAGCTGCTTT
TATAATTCCTTTGAAAGGTCCTAAGCACACAGTGGGCCTCAATAAGTCCTTGCTGATTATCA
CCCCATCACACCTTCTTTCCTAGGCACAGAAATACAGAGAGAAGCAATAATGGGTTCATAGG
GGGTGGAGGGAATCATAACAGGAGCATTGGAACCATCCAGACAGCTGGATCCTCAGTGGCCT
GGCAGTCCTGGCTCCTTCCGGGTGTAGCTTCCTAGGCTGAACACGGACTGGCCTCCAGGAGC
AGGGAGGGTGATGAGAGAGAAAAGGCAGCTCATCCAAGCTGCTGTCTAGTTATCCCCATCTG
TCCATCTGTCTCCTCAGCAAACACATTCTTGGAGTCACCACCTGGAGCCTGTGACTCTCCCA
GTTATCTCAGGGACAAACCCTTGGCTCCTCTTCCACTGGTGCACCTACAGTACCTAACCTGC
TGAAACTGTGGTCATCTGACCCCTTCCTCTCTCTTTCATTCATAGGACGAGATGTAATCAAG

GCTCTGAGGACGATGGTGCCCATATGATATCCACATTGAACTGTCGGTTGTCAGGGCGCTAA
ATCGCTGTAGCCACGGGCAGGCTATGAACTCTAGTGGGTGGCACAGCCTGCCCCCTGCTGCT
CAACATGGAAGCTGCAGGGATAGGTGACCCCTAGCCAGGCTGGAGTCTTTATGGACTCTACT
GGGACAGTGGCCTCCAGACACCGGTTTTGAAAATTCATCTCTTAAATTGAGTTCTCCTGCCA
GATCTCCCCTTGTTCCTGGAATTGAATTGTATCCTCTAAAATTCATATGTTGAAGGGTCTAA
TCCCCAGTATCTCAGAATGTGTGACCCTATTTGGAGATAGAGGGTGTTACAGATACAGTTAG
ATCAAGTCATAATGAAGTAGTGTGGTCCCAATGCAATATGACTGGTGTCCTTATTAAAAGGA
AATGGGCAACACGTGCATGCAGGGAACACAAAGGCAGGGACTGGGATTTGATGCAGCTCACA
CGCCAGCAACCACAAAAAGCTGGGACAGAGTTCTAGAACATTCTCCCTCACTGCCCTCAGAA
GAAATTGAAAGCCCTGCTCACACCTTGCTTTGGACTTCCAGCCTCCAAAATGTGAGACAATA
AATTTTTATTGTTTAAGCCACTCAGTTTGTGGCACTTTGTTGCAACAGCTGATTATGAGCCA
AACTAATACACTCCTCGTCAGGGTGTTCTTAGTCTGGGTCGGAATTGTTTTAAAGGCTTCAC
GGCATGCATATTATGGTCTAAAGATTTCTCCGAAAACTATAAAAATTACACAGTGGAATGGA
AAATGCATTGGATCCCAGAAGACCAGGTTCTAGACCAGGGAAGAGCTGTGTGACTTGGAACA
AATGCTAAGCTCTGAGCATCGTTTATAAATGAAAGATCTGTGCCTGATGCCCTCTAAGGGTG
CCTTTCAGCATTAAACTACATTTGTTTTAAATATTGGGCTCCAATCCTGTCTAGGAAATGCA
AACTGAAATATACACACATACCTGAACAGTCCCAAGCCCCAAAGCAGAGAACTTGTTTTAAG
AAAAAAAAAGATTTAAAAATAACAAAGTAAAGACCGGGTGCTGTGGCTCCACACCTGTAATC
CCAGCACTTTGGGAGGCTGAGGCAGAGGCGGATCACCTGAGGTGCAGGAGTTCAAGACCGGC
CTGGACAACATGGTGAAACCATATCTCTCTACTAAAAATACTCTTAAATTAGCTGGGCATGG
TGGCATGCACCTATAGCCCCCAGCTACTCAAAACTGAGGCAGGAGCATTGCTTGAACCTGGG
GTCAGAAGTTGCAGTGAGCCAAGATCGTGCCATTGAACTCCAGCCTAGGCAACAGAACCCAA
GACTCAATAAAACAAAAACAAAGCAAAACAAAACATAATTCATGAGAGCTTGCTTTAAGAAG
CAGAAGGTGGTAAATTGGAATAAATAATAAGCAGAATGCCCTGCATGATCTTACGAAACCCT
GTGTGATTGGGCTTCCATGTGTTTTAGCTTACGGTGGAGATGACCTGGTGGGAGCTAGCAAT
TTGACTCATCCCTGCCAGCATGTATTTCTTGGACTGCTAAATGTGTCTTTCTCTTGGGAGCC
TGGTTTAGACACTGGTTCAACCAACTCTGAACACTTGACATGAATTTGGGTTCCCAGCCCAC
AAGAGACTGAGGTGCAGCTCTGGGACACAGAAATGCACACAGCTGACCACTCCCGGAGTCAC
CTGGGGACACCCAGCCTGCCCACAAAGTTTTATTCTGTCTCTACTTTTTTTTTTTTGAGCGG
AGTCTCACTCTGTCGCCAGGCTGGAGTGCGAGTGGCGATCTCGGCTCACCTACAACCTCCGC
CTCCAGGTTCGCGATTCTCCTGCCTCAGCCTCCCAATGCTTTAGAACCACAGGTGCCTTTTC
CTGAAACTTAGCTAATTTTTGTATTTTGTTAGAGACAGGTTTCTCCATGTTGGCCAGGCTTG
TTTGAACTCCTGACCTCAAGAGTGATTCGCCTCGGCCTCCCAAAATGCTGGGATTACAGGTG
AACACCATGCAGCCCACTTCTGTATCTTCTGTATCTTCTGATGGAAAGGGAAGATTGAACAC
ATCGGAAAAAAGGAAACTGGATACCAAAACGGTTCCTTCTTCCTCTCCCATCACCTAAACTT
TCCAAATGAATCCTGACCTCCAGATGTGAGGTCTAGCCCAGGCAACTACAATCACTTAGGTA
TGGGGAAATGGAAGTGTGCCCAGATACTTAATAATTTAATCTAGACAATGCTTGTGTTTCCA
AGCAAGAGAGAGCAAGAAATTAGCTAACAAAAATGTCAGACACAAGACAGGTTTAAGAGGTA
AAACAGAAAGGTGTAAAGAAAGAAAAAACACACACTTACAAATAAGGGCAGGGGACAGAAAG
TTAGGATCTAAAGAAATGGAAACCCAAAAAAATACAAATTCTGTAGGGCGTAGTGGCTCCCA
CCTCTATCATGCTTACTTTGGGGCTGGGCGAAACGGAATTCACAGGGGACCAGGAGATCAAG
ACCATCCTGGCCAACATGGTGAAACCCCGTCTCTACTAAAATACAAAAATTAGCCAGGTGTG
GTGGCACACACCTGTATTCCCAACTACTTGGGAGGCTGAGACAGGAGAATCGCTTGAACCCG
GAGGCGGAGGTTGCAGTGAAGAGGTCGAGATCGCACCACTGCACTTCCAACCTGGCAACAGA
GGCGAGACTCTGTCTCAAAAAAACAAAGCATGCCTGAGGTCCTATTCAGGACACTGAGGCCA
AGCAGATTCTTCAGAGGGTCCTCAACAAGGACTAAATGTCCTAAATTCCTGAAGAAGAGGCA
CCGCTGATTGGAGATGGGCAAAGGCTATTGGGGTTTTCAAAAGGGATGAATTACAGGCCAAT
TGGCTTGATGTCGATGCCAAATAATTCTACATCTGAGTTCTAGACTACTGAAAGTTCCTAAA
ACATCTACACAAGCGATTTTTTCCCTTTTTAACTGAACCATTTGGTCCTTTGAAGTGATTCA
TTTAAAGTTATTTTCAGCTATAATTTGGCAAGACTCCCAGCTGTCCTCCATTTCCATTCTCC
CTTTCTTGCCCAACAAATCACCTCTGAATTTTAACTGGGCACATAGCTGTAAAATATAAATT

ATTTCCCAGCAGCTGGTGTTTCACTTCTGAAAGTGTGAGCCATTGGCGCGGTAAAAATGGAA
AGTGTCTTCAAAGGCAGGGCCATCCACTTCTCCCCTTGTTCCTTCCCACTGGTGGAACAGAC
GTGATGACTGGAGGTGGAGCCTGGTCTAACAGAACTGGGAACACAGACACTGTCCCTTCCTG
GGGATGTACCTTCCGGGAACAGAAAGGAGTCTAGCCTGGGCTCTGCATTTGGAAGGGCATTG
ACAAACTCTATTACCAAAGCATTAATCGGAGAAAAGACCAGTGAGAATGTATGGAGTGCTAT
GTCCTGGACACCAGTCTAAGTACTTTACATATTATTATGTAATCTTCACAGTAACGCCACAA
GGTGGAGGTACTCATTTCCCTTTTACAGATGAGAAAAGGTTCATTAACTTGTTCCAAGTCAC
ACAACCTTTGACTCTAGCTACAGGGTCCTATTATTTTGAACCCCTTACCTAGGTGCTTCTCG
ACTTTACTGCACATTGAGTCCTAGAAAACTTTAAAAAACAGGTCTTGGGTCCCCTCAGGTCT
GATGCAACTGTGTTCACCTTGGTGGTGGCTGTATGGAGAAGACAGCAGGAGCCACCAGGACC
AGGAGGACTGCCCAGCAGAAGCTATCCTTACCAGTCCTGGACTATTTCCTCCTAAGAATCAA
GGGTAAATTTGTTTCAGCCACATGGTAGCCTCTCTATTCCTCAAGGCTAAACATAATCCTAA
ACTCCCATACACAGCAGAATTTTAGATATGACAAAATGCATTTACTGAAAAGCTTGTTTACA
TATTACTTTTTTTTTTTTTCGAGACGGGTTTTGCTCTTTGTTGCTCAGGCTGGATGTAGTGC
AATGGGTGCAATCTCAGCTCCTGCAACCTCCACCTCCCCGGTTCCCAGGTGATTCTCCTGCC
TCAGTCCTGAGTGACTGGGACTACTGGCATGCCACACCCACCCGCCCGACTAAGCCTGTATT
TTTAGTGAGAGCCCGGGGTTTAACTCCTCTTGGCCAGGCCGGGTCTCCAAACTCCACAGCCT
CAGGTGATCTGCCTGCCTTGGCTCCCAAACTGCTGGGATTACAAGCCTGAGCCCTGCCTTCA
GCTCACATATTCCAAACACTCAGCCTACCTTTTTAGGTATGGTAGGCTCTTTAAAATGGATA
ATAAGGCTGGGTGTGCGGTGGCTCACGCCTGTAATCTCCAGCACTTTGGGAGGCTGAGGCGG
GTGGATCATGAGTTCAGGAGTTCAAGACCAACATGGCCAAGATGATAAAACCCTGTCTCTAC
TAAAAATACAAAAATTAGCTGGGCGTGGTGGCAGGTGCCTGTAATCCCAGCTACTTGGGAGG
CTGGTGAAGAATTGCTTGAACCCGGGAGGCAGAGATGCAGTGAGCCAAGATTGAGCCATTGC
ACTCCAGCCTGGGCGACAGAGCAAGATTCTGTGTCAAAAAAAAAAAAAAGGGTGGGGGGCAAT
AATAAGGTCATTTATAAAATTTTATAAATTATCTGTTTAAAAATGTTTTAAAGTATGTTGGA
GTCTACAACCAACCCTGAAGGTTATTAAAAGCAATGACGGTGGAGAGCCTGAGGCTCTTGAA
TGATCTCTCCATCAAGTACCGCTGAGGCTTATAGGATGAGTACACTATCTGCGTATTAAGAT
CCACTTTCATCTACGTTACTGCTTGATTTTTAAAGGCTTTCAAGACTACTAAATTAGAAATC
ATGGTTCTCAAATTTTATTACTTATTTTCACTTTGAGAGAAAAATATTTAGATTTCTGTCCT
ATCATGATTGGTTCAGAGACTGGTGACTTTGAAAATTTAATACCTTTGAAAACGTTATTTCA
AGATGCTCCCCCTTTGTCCCTTCATTACACAGTATGGTACCAGGTGGTGTACATTCACAAGA
AGTGTTCTAGAAAAATAATTCCAGATACTTACAGAAACAAGAACTCAAACAATTGAATTTGA
AAGAGATAAAAAGTTCAAACATGCTTTATTATTATACTGGATGGAACTAGGGTAAGAGGTGT
AACTCTATAAAATTTATGCCTGAAAGCAAGCCTAAACCAGGCACTGTGGTGGCTCTGCACCT
ATATTCCAGTGCTTAGGAGACAATGGGAGAAGGTACTTGGGGCAGTTTTGAGACCAGCCTGG
CAACATGGTGAAAGCCCACCTATAAAATAATACAAAAATTATCAGGTGTGGTGGCTGGGTGC
TGTGGTCCCCAGCAGAGGTGGGAGGATTGCCTGAGCCGGGAGTTGAAGACTGCAGTAAGCCT
TGACTCTGCGCCACTGCAATCCAGCCTGGGTGACAGGGTGGGACTCTGAAAAAATAAAAAGT
CAAGCAAGCAGGCAAGCCTAGGCCCGGGCACAGTGGCTTACACCTGTAATTCAATACTTTGG
GAGATCTGGTGGGAGGATCGCAAGTCAGGAGTTTGAGACCAGCCTGGGCAACACAGTGAGAC
CCCATCTCTACAGAAAAATTAGATGGGAATGGTGAGGTGCATGCCTGTGGTTTGGTCTGGGC
ACACTACTCAGGAGGCTGATGTGGGAGTTCACTTAAGCCCAGGAGTTCAAAACTCACAGTGA
GATCTGATGGCACCACAGCACTCCAGCCTGGGCACCGGCAGAGACTATCTCAAAACTAAAAA
TTTTTAAAAAGCAAGCTCATGGGGCATATGTTCTCAAGATCTCCTGAGGGCTGTGTCACTAG
CCAAAAAATAAAAATAAATAAATAAATAATAAAAAAATAAAGCAAGCTATGATCCAAGCTTT
ATAGTCCAACTACCCCAGGAAAGAATCTGGCAGTATTATATATTAAGTTTTGAGGAAAAATA
AAATTTAAAAACATAGGTTTTTAAGCCATGCTGCCAGCTGTCTTTGGAGATGTTAGAGCTAG
GTCTGCATCTGGCTGTCACTTCCTGTGTGACCCTGAGCAAAAAGAAATTACTTAACCCCTCT
AAATGCCATCTCTTCATTTTATAGATGAGGCACTAACTACCTTTACAGGGCTATAGGAATTA
GATGAAGGCATGGCTTGTGGTGCCTGGCACAGTAGATGCTCAATAATGGTGGCTATTATTGG
CTAATCCCAAACTCAGCATAAGCCAACAGTATGACAGGAGACTTACATCAGTAGTATGGATC

TAGGAGACTGTTTGCCTCTCTAACCTATTTGTCTCCCTTACAGTCACATTCAACATTTAACA
CTTGTTCATGTACTTGCTGTCTTCTTGCTCACCCCTAGGGTTTCAATGGTCTGGGAATTTGG
GGCCCAGAGCTTTAGAGGTTGCAAAAGCTTCCCCAAGTCCACCAGCCAGCTCCAAGACCACT
TGCTCTTACATTCTATTATATGAAACACAAGGTGAAGTTACAAAAGGTAGACTACTGTTCTA
GATGAAGAAGTGGCCAAGAGGCTGATGGCTCACGCCTGTAGTCTCCCAACAGTTTTTGAGGC
AGGCAGATTGGATCACTGAGGTCAGGCGTTTGAGACCAGCCTGGCTAACATGGTGAAACCCA
TCTCTACAAAATATAAAAGTGGTTAGGGTGTGGTGGTGCAACGCTTAATCCCAGCCTTCTTT
CGGGAGGCTGAGGCAGAAGAATTGCTTGAACCCGGAGGTGGAGGTTGCAGTGAGCTGAGATG
GTGTGTGCACTCCAGCCTGGGTGACAGAGCAACACTCTCATCTCACAAAAAAAAAGTGTAAG
AGTCATCTAACACCTAGATGACTAATCCTTGCTTTGGGTTGGGGAGAGATATAAAGTCATTG
GAAATTTTAATAAACTGCATTTATTAGATATTGTGGCATTGTTATTTATCTTGTGTGCTGGC
TCATTAGGTATTGTTGGCTACACAGAACACCCTGTATTATAAAAGCATGCTCCAACTGTTTG
AAATTTGAAATACCATGATGTTTGCATCCTACCAAAATAGTCATGACACTGTGTATATAGAA
AGCACACACAACAGATGTTATTTTTATAAGGGAAGATTATACCTGGCATCAGTTTCTTCGTC
TTTTTCTATATATATACATTTTTAAAAATGAAAACCTCATACTAACACTCAGCACGGTCACC
ACGCTGTGATAGCTAAACCCACGCTAGCTATTAAAAGCAGCACTACTTTCCTATTATTTGGT
TCTCTGTTTCCATGAGCACTCAGTGCTGCTGTCTCTGACATGTCTACAAACCACACATAAAA
ACTACAAGGCCTATAGAAAATGGGTTTGGAGATTAGTGCCAATACTCAAATCTTTGTGACAC
ATAAAGATTACCAGTTTCAAATATGTCAATGGTTAAGAAATAAACAGTCTACCTTGACCTGC
AATTTGCGAGCAGAAACACCACTGGACAGCCCACCCAGCTCACGCATTGCCACCCCTGCATG
AAGAGAGAGAACAAAAGATAAAGACAAAGTGAGAAAACATAGAAAAAGAAAGAAAGATTGGG
CTGGGCGCGGTGAATCACGCCATAATCCCAGCACTTTGGGAGGCCAAGATGGGCAGATCACG
AGGTCAGGAGATCAGACCATCCTGGCTAAACACGGTCGAAACCCAACTCTACTAAAAATACA
AAAATTAACATCAGCGTGGTGGCGACACCTCATATAATCTAGCTACTTGGGAGAGGCTAGAG
GCGGAGAAAATGGCGTGAATACCGAGAGGTGGAGCTTGCAGTCAGCTGAGATTGCGACACTG
CACTCCAGCCTGATATGCAGAGCAAGACACTCCATCTAAAAAAAAAAAAAAAGAGCTATAAAA
ATTTGCAGACGCGGTGGCTCACACCTGTAATCCCAGCACTTTGGGAGGCCAAGGCAGGTGTA
TCACCTGAGGTTGGGTTCAAGACCCAGCCCAACCAACATGGAGAAACCCCGTCTCTACTAAA
AACACAAAATTAGCCAGGGTGAGGTGGCGCATGCCTGTAATCCCAGCTACTCAGGGGTCTGA
GGCAGGAGAATCGCTTGAACCAGTGGAGGTTGTGGTGAGCCGGGTGTGCCATTCTCACACTT
CAGCTTGGGCAACATATAGCCAAACTCTGTCTCAAAAAAAAAAAGCTATAAAATTTTAGCCG
GGCACAGTGGCTCATGCCTGTAATCCCAGCTGCTTTGGGAGGCTCAGGCGGGGATCACGAGG
TCAGGAGTTGAGACTGGCCAACATAGTAAAAACCCTGCTAAAAATACAAAAAGAATTGGCAG
GCGTGGTGGCGAACCTGTAATCCAAACTACTTGAGGCTGAGGCAGAGATAGCTTGAATTGGA
GGCAGAGAGGAAGTTTACAGTGGCTAAATCGAGCCACTGTACTCCAACCCGGTCAACAGTCC
GAGACTCTATAGAGAAAAAAAAAAAAAAAAAAAAAAAAAGCTTTAAGAATTTTATCACTAGTGTG
GTTTGTGTATCTAACACCACAGTTGACTGTGGTGGTAGACCAACATACTGGGGATCAGGTGT
GTTGCTCTGGTTTGTTGTTTGTGTTCGAAGTTTCGCTCGTTGCCCAGGCTGGAGTGTATGGT
GTGATCTTGGCTCACTGAAACCTCTGCCTCCCGGGTTCAAGCAAATCTCTCGCTCAAGCCTC
TGGTATTAGCTGCGGGATTACAGAATTTGCCACTGTGTCCAGCTAATTTTTTGTATTTTTAG
TAGAGGCGGTTCACCATGTGCCTGGATTGGTCTCCAACCTCTCTGACCTCGGGCGATCACGC
TCCTGCCTACCAGCTTCCCAAAGTGTCCCAGAGTTGGGTGTGAACCACCCTGCCTGGTCTAT
GTGGTTTTTCTTTAAGACAGAGTCTTGCTCTGTCACCTGGAGAAACGGAGTGCAGTGTCGTG
ATCTTGGCTGCATAGCACTCTGCCTCCTGGGTTCAAGAAATCTGATTCTCCCTGCCTCAGCC
TCCTAAATGCTTCCTGGGATTACAGGCATACTGTTCCACACCCAGCTAATTTTTGTATTTTT
AGAAGAGACGGGGTTTCTCTATGTTGGTCAGGCTGGTCTTGAACTCCCGATCTCAGGTGATC
CACCAGCCTCGGCCCCGAAAGTTACAGGCATGAGTCACTCGCCCGGCCATTTCTAAATCAAT
CTTCCATTTCTAAAAATTTTGTCCTTAAAAATGTTATAGGCCGGGCACGATTGGCTACGCCC
TGTAATCCCAACACTTTGGGAGGCCAAGGTGGGTGGATCACTTAAGGCCAGGAGTTCGAGAC
CAGCCACAAGCTAACATGGTGTAGCCATATCTCTACTAAGCTAAGAAATTAGCCAGGTTTGG
TGTGGCCCCTGTAGTCCCAGATACTAGGGAGGCTGAGACAGAAATTACTCGGAACCCGGAGG

TGGGGTTTGCAGTGAGCCGAGGATCTCGTCACTGGACTCCAACCTAGGCGAGAGGTGAGACT
CCGTCTCAAACGTCGAGGTAAAACACAAATGAGGTTGAAAAGAGAATAAAAAGGAAACAGAG
TGTGGCAAACCCAGGAATGGCGATGGAAGGGCTGCGGCTTGCAAGCATGAGATGGGGCGTGC
GGTTCTCTGGAATCAGATGTTTTCCCACACAGGGGACGTGTGCACTTGTGTGTGTTCCTATG
CAACTCAGTTCAACTGGGTGCTTTTGTGTTCCTAGTGCTACTTCCACAGGCAAAAATTACAC
ATAAACTCATGTTATGCTCAGAGCATGCCAGCAGGACTGCCCTCCGCCAGAGCGCTGACTTG
ACCCTCTCCTCAACCTCAACCCTAGACCTCGGGCCTGCTTCTGTCCCTCTGATTTCTACCGT
CTCTAGCTCTAGCACTGCTTTCTCAAAGTGATCTTTTAGAAAGGATGTGGGTCAATTATAAG
CTAGGGGAGGTAGGACATAGGTGGTTCCTATTTTCCAAAGTTCTTAAGCAGGTCCCACTTGG
AAAGGGGCTCGTGGTCTAGGCAAGCTGTGTCAGCCACTATCTACCAGAATGAACAAGCCCTG
CCACTTCCACCTCCCAACTCTCAGAGTAAAATGCTTTTCAACTTTGTGATCGAAAGAAATGA
GTAAGTCAATATGCCAAGATTTTCCTACCATTTGTCCAAAGACAAACACTTTTTTAATGTAA
TCTTTATGTATTTATTCACACATTTTGACAAAATGTTACAGTTAGAGTGAAATCTATTCTAA
TAATACAAGAGTACGCTGTACAAGACAGACCCCAAGTCTCTGGTCAAGAAATTGCCCTAAGT
CAGAACAGCAGAACAACAACTCCTTCGCACTTGTTGCGTGATAATCTGTGTTGTCATGGCCT
TGCCACACACCTATATTCTCTCTCTCCTCTTCTTGCACCACATCCCATCCACATCCCACTTA
ATTGCAGTTCTTTGGTTTGGGCCTTAACCAGGGACTCTACTGACTACCATGGGTCCTAGAAA
GAAGAAGTAATAAATGAAAAATAACTGGGACCCTTCGATGCATCTCTCCTCTTCCAACAATG
ACGAAGAGGCAAGACATACTGCTGGAGACAAGCTACTTCCTTGGCACAAAAATGAACAGGCA
ATAAGAGGTCAAGGGAAGTGTTTAAGTTGGTCTCAGGTTTAAACCACTTTTCAACACCCACA
AAACAGTAGCAAGCAGGGAAAAAAAAAGCAAAAACAAAAACAAAAACTCCCAACACCTCAAG
ACTCCAGAAAAAAAAAAGGGAGGAGGAGGATTTAAAACTTGATCCTAACTATTCCTAACAAAT
TGCAGCATGACCATAAACAAAACCAGCTCGGTCAAACTGACGGCAATCCGTTGTTGATATAT
CGCAAAGACAGTGTGGTTCACTTTTATTTTTGGCTCTGAAGAGGGAAGAAAAACTTTTGGGG
GGAACTTAATGGTACAACATAAATAAATCTCCACTGTGTGGTATTTGAGACAAGATTACATC
TATGCATTCACACAGCTTGTCTGTAGATCTGCTGAGAGCTCCAAGGGAGTGGCCCAGCCCCA
TTCCTCTGACTTAGCCTTCTGAAAAAGACAAGTCAAACCTGATATGAAAAATAATGCCTGAA
TCAAAATGGTGTTTTCTATACAATGGGACTAGGAATTAGAATCCTGCTCAATTCCTCAGCTC
CCTATTTGGCTAAGTTAAAAATTACTAACTAAATTTAATGTGATGAACTTTTGGGCATCAAA
AGCCCACTATCTGAGAAGTTAGTCTCGGGTAATTGGCAGTGTCGAGAAGAGTTGGGAAGGGG
AGGCAATGATCCAATGAATATAGAAAGAACTGGCCCGATTCACAGGAAACTTTTACTTTGGA
TAAGGTGGGTCAGCGTTGATGTGCAGGCAGGAAATTTCTTTGTACCAAATTAATAGCCCTGA
CCCGGGGCATAGCCCAGAGTTCAAGACCAGCCACCAAACAGAGTGAAACTCATCTCTACTTA
AAAAAAAAAAAATTAATAATAACTGTCTCTCTAGAGATAAAGCAAGGTCTGGATCAAACCAA
CAACATAATAAAGTATATCGTTTTTATTCCACATCATGAGTTGTTACCAAAGAGCACTCCCA
GTAGCCCCTCATTTTTGAGACTCAACTGGCCAACTATACTTTCAGGTGGACCTAGGAGGAAC
TGAAAAAAAGAAAGAAATCCAGCGTTTCTTTTTGGCTCAGTGGGTGGGATTTGGAGGATATC
CAGCAGAAACTATAAGAAAGCAAAATGGAGGGTCCCATCCCCTTCACAAGCCTGAGGTTTGA
GAGGTTTGTTCCAGATTAGCTCAGTGAGATGCGAATTTCAAAACAGTGGTGAGAATATGACA
ACACTCCCGCAAATGTAGCCCTTCTTCTGTTTTGATAAGAGCAATAAGAATCTGAATGAAAT
GGGACATCAGTTATTGAATTATCTTGCACTGGAAGTTACAGCAGATGCCTTCTAATACATGT
GGCATGTCCCCCTTCTACAGCAGGTGATAACGGACTGAGACAGGGCTAAAGCCTGCCATCCC
TTCTTATCCCAAAGCCTGTCAAGCATACTCAATCCGAACAGGACCACAGCTGCCCTGGTTCT
TTCGCTCAGGGACTGGCTGGTGGCCTTGACTCTACTCATTCCAGAGGCTTAGCTGAAGATGT
AGTCCTTTACTTTAATCTCCATATTCTTGGCTTTTCAGGTGAGCCATGTGCAGCTTCTCCAG
AAATCTGGCATGCCTGTCCGGAGACGAGAATCGGGATAGCTTTGACATACAAACTAACAGTA
GACTCAAAATATTCGAAGACCCTATTTCTTTGAAGTGGTAGAACTAGTTCTTCCTTATAAGG
AATCACACTTCTGACTAGCCTGTGAATAGAGCTGCCTCCCGGAATGTGTTCCCAAAGCATGG
GTTGGCAGAAACTCTGTATGTAAGAAAAAAAAGAAGAACCAGAAGAGAACGTTTTATTTCCC
CATTAGGAGAGAAAACAAAATGCTCAACTGGGTTCCAGGCCACTGCATGCTGCCCTTACTTA
CCACTGGAAACCATCCAAAATAACACAGTAGCGAGGAAACACCGTTTGGGGGATTGTCACTG

TATGTTGGTAAGTAGTCCATAAGCCATTCTGGAAAAGAAAAACAAAAATGAAGATCCTGAAT
CACCAACCACAACCTCCACTATCCAGTACCATAGAATAACTAGTCTGATTCTGGAGCAGGGA
GTGTCATTTTCTTTTTCACCCTGTAGAAGAGTTAACACTGAAAGGCCTGAGAGACTACTATC
TATAGAAAGGTCTCCTTTGTCTTATTATGGCCAACTTAGCTCCCAGTCAACAGTTAGCTGAT
ATGGGTGGCTCCTATTGCATGAACTATGCAAAACCTTTGATGGTTTATACTGAACCACAGGC
TTTGCTTTGCAGGGTTTGGAATTCTGGTATGTGCTAAGCAGAGCGGAACAGTGACCAGCTCC
CAATAAAAATTTTGGGTGGTGGGGCCAGGCATGGTGGCTCATGCCTACAATCCCAGCACTTT
GGGGGAGGCCGAGGCAGGCGGATCACCTGAGGGTCAAAGTTCAAGACAAACCTGGCCAACAT
GGGTGAAACCCCATCTCTACCAAAATACAAAAATTAACCGACCATACTGGCACACACCTGTA
GTCCCAGCTACTCGGGAGGCTGAGGCACAAGAATTGCTTGAACCCGGGAGGCGGGGTTGCAG
TGAGCCCAAGATCCCCAGCCACTGCATTCAGCCTGAAACAGAGGCGGACTCCGTCTCAAAAA
ATAAATAAACATCATCATCGTGGGTGTTGGCTTAGTGTGAGGTGGCTCATGTACTATAATCC
CAGCACTTTGGGAGCCAAAGGGCAGGCAGATTGCCCGAAAGGACCAGAGTTTGACCAAGCCT
GGCCAACATGGTGAAATCCTATCTGGTTAAAAATTATGTGTGGTGGTACTGCGCCTGTAATC
CAGTGCCTTGGGAGTCTGAAACTGGGAGAATCGTTTGAACCGAGGAGCACAGAGGTTGCAGT
GAGCTGGAATTGAGCCACTGTACTCCAGCCTGGGTGACAGACCAAAGACTTTTTTTTTTTTT
TTTTTTTTGGGAACAGAGTTTCGTTCTTGTTGCCTTGGGATGGAGAACAATAGCACAATCTT
TCGACTCACCACAGCCTGCCTCCTGAGTTCAAGTGATTCTGCCTCAGCCTCCCCCAGCCCGC
TGAGTTTGAGCATGCACCACCATGCCCAGCTAATTTTGTATTTTCGGTAACTGGGGCTTCTC
CATCTTGGTCAGGCTGGTCTCGGACTCCGACCTCAGAGAGTGATCCACCCTGTGGCCTCCCC
CAATGCCTGAGTTAGCGTGTGCCACTGCCAGCCAGACTCAGTCTTTAAAACACTTTGGGCCT
TGGGTGTGGTGGCTCACGATCTGTAATCTCAGCACTTTGGGAGGCTGGTGATGGATCACCCA
GGTCAGGGGTTTGAGACCACCCTGGCCAACATGGCAAAGCCCTGTCTCTACTGAAAATACAA
AGAAAAAAAAACTAGCGGGTGTGGTGGCACACACCAGTAATCCCAGCTACTCGGGAGGCTGA
GGCAAGAGAACTGCTTGAACCCGGGAGGCGGAGATTTGCAGTGAACCAAGATCACGCCATCG
CACTCCAGCCTGGGCAACAAGAGAAGCGAAAACTCATCTCAGAAAAACAAAACAAAACAAAC
AAAGCAAAAAAAAACAAAAAAAAAAAACACTTGGGTGTTTTTTTTTTTCTTGAGACAAAAGGAGT
CTCACTCTCATTGCACAGTCCCTGGGTGCAGTGATGCAATCGACTCAGTACAAGCCCTCCAC
CTCCTGGCTCAAACGGTCATCCCCACCTCAACCTCCTGTGTAGCAGGGACTACAGGCATGCA
CTACCACCACAACTAATTTTTTTATTTTTTGCAGGGCTGGGTCTTCCCCTGTGTTGCCCAGG
CTGATCTCCAAGCCTCCTTAGCTAAAAGAGATCACCTCCTGCCTCAGCCTCCCAAAGTGCTG
GAGTGCAGGCATGACTACCGTGCTCAGCCAGGTGTTAAATTTCTAATGGCCTTCCTAAGCAG
AAACATTGCACACATATTGCTACATTTCTGCTGGGAAGGGAAGCTTCACATCGGACTCCTCC
AGACTCCTCTGTGCCTTTTTCTCTTGCCAGTCCAGCTGCATATCTTTACTCTGACACTGTCA
TAAATCTTAACTGGGGGCACAGGCACGGTGGCTCATGCAATCCAAAAAGCACTCAAGCCGGC
GGGCGGATCACTTGGGGTCAGGGGTTCGAGACCAGCCTGGCCAACACTATGGTGAAGCCTGT
CTCTAATAAAAATACAAAAATTATGTAGGCAAGGTATGGTGCACATCTGTAGTCTCCTAGCC
TGGGAAATAAAACGGGAGAATTGGCAGACCTGTAGGTGGAGGTTGCAAGTGAGCTGCTATGG
AGTCGCTCACTGCACTCCAGCCTACAACAGAGGCAGACTCATTCTCCCAAAAAAAAAAAAAA
AAAAATCAATATTTTGTGCCTAATGTACATCCTGAAGACAAAAAAGACCACAAAATTTTATT
CTTCGTATTATTGGGTGATCTTGCTATTATTGTTATTCTGAGGCTGTGTATTTTAACCATTA
AGTAGTTTAATATTTGGGGTTCCTGGGAATTAGGTTCTACAGTACAGGGAAAGACAAAAGAT
AGAAGATAGAAATAAAAAACTCCATAGTCCTGAATTTGAATTAAAAGTGTTAAGTATATACT
TAGGATTTATTTTATCTTAAAATAAACATACCAGTAAAATGTATTACTTGGTTCCCTTGTCT
TCTGAAAAGGCCTACAAACAATAACGACCCAATAACTTAAGATATTCTTTCTAACAACCAAA
TTGTGGTATTTATTTTTATTTCACTAAAAGGAACAGGACTCCCTAAAAAAATGTTAATTTCCA
GGTGTAAAACTAAAAATATACAAGATGAACCTACAACATCTTGTAACACCAGAAAACAAGAC
AGCTTCTGAGGTCATGTCAAGAACTTGAGCCAACAAGAAAACAATGACCAAAAACTGGAATC
AATTTATAATAACCTCTATGGATAATATATTTAAAAAACTAGAAAATTTATTGAATCTGTCT
TTTTTTTTTTGACGGGAGTCTCACTGTATGCATCCACGCTGGGGTACAGCCACAGTCTTAAA
CTCCTGCAACCTCTGCCTCGGGTTCAAGCCATTCTCTCACTTGCCTCAGCCTCCCAAGTGGC

TGGGATTACAGACACGATGCCACAATGCCTGGATAATTTTGGCACTTTTAGTAGAGGCAGGG
TTTCACCATGTTTGGCCAGGCTGGTCTCAAAATCCTGACCTCAAGTTATCCACATGCCTCGG
CCTCCCAAAGTGCTGGGATTACAGGCGTGAGCCACTCGCCTGGCTTTTTATTTTTATTTTTT
TTTGAAAGAGGTCTCAGAAACTGTTGCCCAGGCTGGCATGCAGTGGCACAAACACGACTTAA
ATGCAGCCTTGCCTCCTGGACTCAAGTGATTCTCCCAACCTCAGCTTCCCAAGCAGCTGGGA
CTACAGGCACATATCACCATGCCTGGCTAATTTTTATTTTGTAGAGACCGGGTCTCTCCCAT
GCTGCCTGGCCCAGAGCTGTTTTTACAAATTTCTTGGGCTGGGCGCACAGTGACAATCCGCC
TCTAATCCCAGCATTTTTGGGAGGCTGGGGTGGGAGGATTACTAGTCAGGAGTTTGAGACCA
GCCTGCGCGCAGCATGGTGAGACTATACCACAAAAAAATACAAAAATAGCCTGACATGTTCA
AGCACCTGCGCCTAACATCCCAGCTCCTCGGGAGGCTGAGGCAGAAAATGCCTGTGAGCCAG
GAAGAAGTCCAAGGCTGCAGTGAAATGATTGTGCCACTGCACACTAACCTGAGTAACAGTCA
AGGGAGACCCTAATTTCAAAAAAACCCAAAAATCCTTATTGAGATAAATACTGAAATATATA
ATGAAAGACTGGGTTCTCCTACGGTAAAGCTAAATTTGCTTCAAAATAATCAGTGAAAGAAA
TGGAAAGGTATAGATAAAACAATTTTTGGCCATAAGTTGATAACTGGAGCTGAGGAGGATGG
ATGGGGGTTCATTATACTCTTCTGCTTCTGTTACATCTTTTGTATCTATTTCGTTACCATGA
TAAAAACCCCTACCTTAAACAAAGTCTAATTCCATTTGGCAAATCCAAACTCAGCTACATGG
TTTCGGAAATTTGCCCTCTGTACACAGTCTGGCAATTTATCACTTCTCCCCTAACCTCTACT
TTCCTCTCTAGCATATGGGTCTCTCCAGTGCATACCTGGCTCATTCCTATCTTTGTCTTTAC
TTGAATCATGTCTTCTAACCCTAGTGTTCCTACCTGCTCCTCTCTCTATCCAGTGTACTTTT
TTTTTTTGTTTTTACAAGGACCAACTCAAGTCCTTGAGGTGAGGTATAAATAAAACAACTTA
GTGAGAATAAAGAAAACTACACTAAAGAACACTGTCCCATATAGCCTAAATCGGGAACACAA
GCAATGAGGAGAAACAAAGCCATTATAAGGTGAAGTGGAACTAAAACCAGCAGCCACTAGCA
TCCCAATATCTATATACTAGTGGAATTTAGCTTAAAAGATAAAAAAATAAACACAAAAACAC
CTCAGCTAGGCTGGGAAATAAATGAATTCATCTTTTCAAAAGAAAGGTCACAGAATTTTAAA
AGCCCAGAATAGCATGGGTCCAATCTTCCCAAATGTAGGAAGCAGAAATCCAGAAAAGTTTC
TATATCGGTGCTCTGCCCACACAGGCCAAATGTTGACAGGAGGTGCTCGCTCTGGATTAAAG
TCTGCTGATGCAGGCTAATCGTCAGTGGCTATGATTCTTTTAACTATCTCTCCAGATAGAAG
AGGAACCAATGGATTACCTACTTTGTCCCTTGGGGAACTTCCAGGTACATACCCTACGTCAG
GAGTTCCCAAAAAATGGTTCCCAGACCAGTAGCATCAGACTCCCCGGCGAGAACTGTTCCTG
CATACCCCCTCCTCTAGTTCGTTATGATTATTATGAAAATTCTAGCCATGACATGTTATCTC
TTGGATGAGGGTGAAACTCACCTCATCAAATGACTTGTGAGAGAATGGATAACCATTTGGGA
TTCATGTAGTCTGACCCTGACAGACTGGAACTCAGCACCTAGGATGCTCCACAGCACTGAAG
GAGGAAAGGAGAAGCAAACTCTTAGTAATGTCTATATATAAAACAACAGGAGAGCTAAGTTA
AGGGAAGGCCTAAAAGCTGGCTGATATATTTTATTACACAAAAACCACAAGGAGTCTACTAA
GAGTAGATAAATACAAACTAGGTTCTAAAAATGGCGAAGGCAGTAGTGGTGGCTCATGCCTG
TAATCAACACTGGGAAGCCAGAAGGAAGATCACTTGAGGCCAGGGGGTCCAAAGACCAGCCT
GGACCACACTGCTTGAAACCCTGTGATACACAACAAAACAAATAGATCTATTAAAAATGAAA
TGAGTTCTTGGTAATTCGGCTCACTAGGCTGTGGGTCAAAACAACAGCACTTCTTTTTTGAT
TACCAACATTAAAATTATGGTTTAGCTAAAAGCTTGTATCTCTCCTTCTTTCTAGACAGTCA
GGTCCCTCATCTTCATGGATGGAAGGGGCGGCACTGGCTGCAGGGAAGCCTGTGGGGAGACT
CATAGGACACCTGCAGAATATACAAACTCAAGTGCGTAGGAATGAGGCCACAGCTAAAGAGA
CTCGAGCAGCAGCACAAGGCAGCCATACATACCCGCGACACCAACACCATCATGTTGTTTTT
CCTGATCCACACTATACCGCCGAACATAAACATAATCCATTGGTACATTGGATACTAAAGAA
ATGGAGGGGCAGGATTAGTGTTCTGCATCACACAAACCTGGACAGAAGAATAAATCAAGTAT
AAAAATACTCACGGAAAATGGGTTACCCAAGTGAAGACACCGGAACCACTAACCACATCCCT
CTCGATCACCTCAACTTGATTACCAGGGCATCCCATTTTTTTCTATACTCTGTGTCCAGCTG
CAGAAAGAGAAAAGACCATGAATACCCAAAGAAGACCCAAAACAAAAATGCAGAAAGCTTTC
CAGATACTTTGGTCTCTGATCCAAGAAATCTTATTACAAGGAGAGTAATCTGGCCTAACAGA
CAGTGCCAGGTGCAGAGACATGGCTGTGTTTACACAAGAATGAGCAACAAAATCACCTTATA
CAGGCTAATGTCATTACACTGAATGTGAATCTTCATTAGACAAAACCAAGGAAAAGTCTTAA
CAAAATATTTTAATAGACACATCTGGGAAAATAATATATGAGTGATATGAATGTGTAAAGAT

GCAAAACAATTCCAAAAGTTTTAAACAACAAAAGTCCAAAAATTATTAAGTAGACACTTCCA
CAAAAATACCAAGCAGCATATTTTTTTAAATTTCTGGCATCACTAGTAACCCGAAGATTTTC
TCTTCTAAGACAGATATACCATATACTATGGAAAAGCCAACAAAATTCTAAATAACAAGTCA
GTACCTCTGGGGAAGGTACTGCGTTAGAGTGAGTGTTATAACCAGCGAAGTGATTCAGGAAG
TCCAATAAAACAAATGAAAAATCCAGAAAGAAAGAATTTTGTTTCTATAGAACTCTGGCGAT
CCCACCTCCTGTTTCATTTTTTAAACCATCTTCCCCCCTACAAGGAGGAAAGCAGTCTTTGA
ACAGAATCTCTACATCTTTATGGAAATAAACATTAGGCCCAACACAGTGACTCACCTGTAAT
CCCGGCACTTTGGGAGGCAAGGCAGGAGGATCACTAGAGCCAGGAGTTCAGGAACCAGCCTA
GGAGACATGGGAGACCCTGTCTCTAAGAAAAAAAATTTTAATTAGCCAAGTGTGGTGGCGCA
TGCCTGTAGGCCTGGAAGCTGAGGTGGGAGGATCACTTGAGTCAGGAAACCAAAGGCTGCAG
CAGGAACATGATCACACCACTGTACTCCAGCCTGGGTAGCTTGGCAGGTTGTCTCCAGGAGG
GAAGAAAAAAAAGCCCTCCACTATGATGTTCTTGTTGGGTTAAACAGATGTATGTCATAATA
TAATCCCTATCTGTACTGAAATTGGAAGAAAAATACTAAAGGTTTATTGGGTCAACTGATAA
AACTGATAAATACAAACAGAGGACTACATGATGTTCCATCAGTGTTAAATTTACTAATTTCA
ATAATGGTATCATGATTGGTTAAATACGGCATATGGCTAGTTTTAGGAAATACACACTGGAA
GAATTTGGGGTAAAGGACTATGATGTATGTAAGCACCCCACTGCAGTTGTTTGGGGAAAAAA
ACAATAAAAGCCTCATAAAATACATTAAAGTATTCATATCATTCCTCTATCTGATATTCCCC
AACGGTCCTGTGGGAGGTAGAAGCTGATCTCATCCCCATGGGACAGATAAAAAATTCTAGGG
TCCAAATGTTTACCTAAATGCATACAATGTGGTGTTAAGGTAGATAGATATGGATGTGCCAA
GCTAATGTCACCTGAGTCAGATAAATGATTCATGTAGACAGTAACTAGTCAATGTGCTAGGA
GGCTCCCAAATCTGGAGAAAATTAACTCACCTGAACATTGAAGAACTGCCGAGGTGTCACAT
CTGTGTAGGTTCCAAAAACTATAGGGAATGAAAGAAAGAATGAGGATGCTGGCATACTCCCG
GTCACTGGCAGCCTGAATTTCTTTCACCTAGCGGCGTTCAGATCTAAAGTACTAAGAGTCTT
AAAGGCAGGTTCAGAAACTAGAAGTCTTAGTGAAACAACAAAAGAAAGACAAAAGGGATGTA
AGCAAAGACAACACTGAGACATTTGTCATCTGGAGAAGAAAAGCAATAAACAAAAAAACTAC
GGCAAATTTTTCTGAAAACAACCAGAACAGTTTGAGAAATTATGAAGAACAATCCCATGCAC
CTGATGCAAAACTACTGGAACCCAAAAAGATAGCCACACTGGGCCTCACCTAGGTCTTTGGT
AGGAGTGGATTTCTGTAATTGTTGTACAGCTTAAGTGTTTCTTATCCATCACCATTTCCCCA
AGGTTGCTCTTGCCCTCTGAATCTTCATTCCCCTTCTGTTTGGGCTTTTGGTTCCTGGAGGT
GGGTGCTGGGACTCAAACTCTTCTGACATTTCTTCCAACTTCATCTCATTAATAGATCTGTA
AGGGAAAAAGAAACCATGGATAGGGTGGTGCTTGAGTCAGCCCTGTGAAATGGGTGTCCACA
CAAAGGTCTGTGCACGAAGGTTATACAGTAAACCACAGTATGTGAGTATATAAACCCAGGAC
ACAGTATAGTCAATAAAAAACTGAGTTCCTGAGTTTGAATCTTAAAGGTCAACACTCACTAA
CTGAATTATTATTTAACCAGTTCTATTTCTAAAATCTATAAAATGGAAATAATATAATAGTA
CCTATCACAAGGGTTGGCTATAAGATTACAAAGAGTGTCAAGTGCTGCAGGCGTGGTGGCTC
ACGGCTGGCAATCTCAACACTGGGAGGCAGAGAGGGATTGGATCCATGGGGTCAGGGAGCTA
AATGGCCTGTCCAACATGGGCAAACCCTGTCTCTACTACAAACACAAAAATTAACCGGGCAT
GGGTGCGCGCAACTGTAATCCCAGCTACTCAGGGAGGCTGAGGCAGAGAATGACTTGAACCT
GGGAGGCAGGGTTGCAGTAAGCCGAGATCACGCCACTGCACTCTAGCCTGGGCGACAGAGTG
AGACTCTTGTCTCAAAAAAAAAAACAAAAAACAAAAACAACGAAAAAAAGTAAGTGTCCAAG
TGCTTATATCTATGGCCAGGCACAGAGGCTCACTCCTGTAGTCCCAGCACTTTGGGAGGCCA
AGGCAGGGGAGGAGGACTCCCCGCTTGAGGCCAGGTTCTGGGCTAACCTGGAAACCTGGTGA
GACCCTACCTCTAAGAAAAATTTAAAAAAATTAGCCAGGCATAATTGGCACATGCCTGTGCT
CAAGCTATAGGCTGAGATGGGAACATCACCCAGGCCCAGGAGTTGGAGGCTGTAGTGAGCTA
CAATTCTGCCACTGTACTCCAGTCTGGAGCAATACAGTGAACTCTGTCTCTTTTTTTGTTTG
TTTTTTGAAACAAGGTCTCACTCTGTCCCCAAGCCTAGGAATAAGGGAGTGCCATGATCTGG
GCTCACTGCAACTTCCATCTCACTGGGTTCAAGTATTTCTCCTACCTCAGCCTCTCCCAATG
TGCTGGGACTACAGGCCACCTGCATCTCATGCCCGGCTGATTTTTTGTATTTTAGAAATGGG
GTTTCACCGTGTTGGAGACGGTCTCAATCTCCCTGACCTGCAGTCTGCCACTTCTCTCGGCC
TCCCAAAGTTCTGGGATTACAGGGCGTAAACCACCGTGCCCAGCTACACTGTCTCTTAAAAA
TAAAATAGGCTGGCGCAGTGGCTCACGCCTGTAATCCCAATACTTTGGGGGGGCTGAGGCAG

ACGATCACCTGAGGTCAGGAGTTTGAGACCAACCTAGCTAACGATGGAGAAACCCTGTCTCT
ACTAAAAATACAAAAGTGCTTTAGACGCGATTGGCACATACCTGTAATCAGCTATAGGAACT
GAGGCAGAGAATCATAGAACACAGTGGGGGGTTTTTGAGGAGCAAGATGTTTTCTGTGCCTC
TATATTCTGGTAAGCAAGGCGAAAAAACTCCGTCATAAATAAATAAAATAAGCATAAATGAA
AATAGAAATGAAATGAAATAATAATGAACTGAAATGAAATGTAAATATAAATGAAATGAAAA
TGAAATGAAAATGAAAGTAAGAAATGAAATGAAATAAATGAAAATGAAAATGAAAATGAAAT
GAAATGAAATGAAATGAAATGAAAATAAAAAATAGAAATGAAATGAAATGAAATGAAATGAA
ATGAAATGAAATAAAGTAAATGGCGCAGAAATAATGAAATGAGGAAATGAAATGAAATGAAA
TGAAGTGAAATGAAGAATGAAATAAGAAATGAAATGAAATGAATATAATACAAATGAAAATG
AAATATGAAATGAGAAATGATGAAATGAAATGAAATGAAAATGAAAATGAAATGAAATGAAA
ATGAAAAATGAGAAATGAAAATAAAGAAATAAGAAATAAATGAAATGAAAATGAAATGAAAT
AAGAAATAAAATGAAAATAGAAATAAAATGAAATGAAGAAATGAAATGAAAATGAAATAGAA
ATGAAATGAAATGAAATGAAATAGAAATGAAATAAAAATAAATGAAATGAGAAATGAAATAG
AAATAAGAAATGAAAATAAATAAAATATAAATGAAATGAAATGAAATGAAATGAAAGAAATG
AAAATGAGAAATGAAAATGAGAAATGAAATGAAAATGTGAAATGAAAATGAGAAATGAAATG
AAATGAAATGAAAATGAAATGAAATGAAATGAAAATGAAATGTAAATGAAAATGAAATGAAA
TGAAAATGAAATGAGAAATGAAATGAAATGAAATGAAATGAAAATGAAATGAAATGAGTAAA
TGAAATGAAAATAAGAAATGAAATGAAATGAAATGAAATGAAATGAAAATAGGAAAATGAAA
TAATGTGAAATAAAGAATGAGAAGCTGAAACGAAAATGATGAAAATGAAATAAAGTAAGAAA
TGAAATGAAATGAAAATGAAATAATATAAATAGTAAAAATAATATAATAACTAAATAAAATG
AAATGATAGTGAAAATGAAAATGAAATGAAATAAATAAGTAAATGAAGTAAATGAAATAGTA
AATGAAATGAAATGAAAATGAAATAAAATGAAAATGAAATGAGAAATGAAATAAAATGAAAT
GAATAAGAAATGAAAATGAAAATGAAAATAAATGAAATGAAATGAAATGAGAAATGAAATGA
AATAGAAAATGAAATGAAATGAGAGTAGTATGAAATGAAATGAAATGAAATGAAATGAGAAA
TGAAATAATGAAATGAAATGAAATGAAGAAATGAAAGCAAATGAAGCAAAATGAAATAAGAA
ATGAAATAGAAATGAGAAATGAAATGAAATGAAAATGAAATAGAAATAAAGAAATGAAATGA
AATGAGAAATAGAAATATAAATGAAATAGAAATGAAATGAAATAAAGAAATGAAATGAAATG
AAATGAAATGAGAAATGAAAATGAAAAATGAAATGAAATGAAATGAAATGAAATGAAATGAA
ATAGAAATGAGAAATGAAATGAAATGAAATGAAAATGAAATGAAAATGAAATGAGAAATGAA
AGTAAGTAAATGAAATGAGTAAATGAGAAATGAAAATGAAATGATAAGAAATGAAATGAAAA
TGAAAATGAGAAATAGAAATGAAATGAAATGAAATGAAATGAAAATGAAATGAAATGAAAAT
AGAAAATAGAAATAAGAAATGAAATGAAATGAAATGAAATGAGGAAATAAAATAAAATAAAA
TAAAAAATAAAATAAAAATAAAAAATAAAATAAAAATAAAATAAAAATAAAAATAAAATAAA
TAAAAGCAAAATAAACATAAGCAAATATAAAATAAAAATAAAATACGACAAATAAAAATAAA
ATAAAATAAATAAAAATAAAATAAAATAAATAAAATAAAATAAAATAAATAAAATAAAATAA
AATAAGAAACAAATAAAAACTAAATAAAATAAAAATAAAAATAAAATAAAAATAAATAAAAA
ATAAAATAAAATAAAAATAAAATAAAAAATAAAATAAATAAGAAAATAAAATAAAACTAAAA
TAAAATAAAATAAAATAAAATAAAATAAAATAAAATACAAAATAAAAATAAAAAATAAAATA
AAATAAAATAAAATAAAATAAAATAAAATAAAATAAAATAAAAATAAAATAAAATAAAATAA
AATAAAAAATAAAATAAAATAAAAATAAAATAAAATAAAATAAAATAAAAATAAATAAAATA
AAAATAAAATAAAAATAAAATAAAAATAAAAATAAAAAATAAAATAAAATAAAAATAAAATA
AAATAAAATAAAAATATAAAATAAATAAATAAAAGCACAAAACAAGTAAAATAAAAATAAAA
TAAAATAAAAATAAAATAAAATAAAATAAGAAATAAAATAAATAAAAATAAATAAAATAAAT
AAAAATAAATAAACAAAATAAGAAATAAAATAAAAATAAAAAATAAAATAAAATAAAAATAA
AATAAAATAAAAATAAAAATAAAATGAAATAAAATGAAATAGATAAAGATAAATAAAATAAT
AAAATAAAATAAAATAAAATAAAATAAAAAATAAAATAAAATAAATAAAATGAAACAAAATA
TAAACCCAAGAATATAAAATAAACTAAAACAAAAAAACTAAAATAAAATAAAATAAAATAAA
ATAAAATAAAATAAAATAAATAAAATAAAAATAAAATAAAATAAAATAAAAATAAAATAAAA
TAAAATAAAAAAATAAAATAAAATGAGCAAAATAAAATAAAATAAATAAAATAAAATAAAAT
AAAATAAAAATAAAATAAAATAAAATAAAAATAAAATAAAATAAAAATAAATAAAATAAAAT
AAAATAAAAATAAAATAAAATAAAATAAATAAAATAAAAATAAAATAAGAATAAATAAAATA

TAAAATAAAATAAAATAAAATAAAATAAAAATAAAAATAAAATAAAATAAAATAAAATAAAA
TAAATAAAATAAAATAAATAAAATAAAATAAAATAAAATAAGAAATAAAATAAAAAATAAAA
TAAAATAAAATAAAATAAAAATAAAATAAAATAAAATAAATAAATAAAAGAAATAAAATAAA
ATAAATAAAATAAAATAAAATAAAATAAAATAAAATAAAATAAAATAAAATAAAATAAAATA
AAATAAGAATAAGAATAAAAATAAAATAAAAATAAAATAAAATAAAATAAAATAAAATAAAA
TAAAATAAAAATAAAATAAAATAAAAATAAAATAAAATAAAAATAAAATAAAATAAAATAAA
AATAAAATAAAATAAAAATAAAACAAATAAAATAAAAATAAAAATAAAAAATAAAATAAAAA
ATAAAATAAAATGAAAAATAAAATGAAATAAAAAATAAAATAAAATAAAAATAAAATAAAAT
AAAAATAAAATAAAATAAATAAAAAATAAAATAAAATAAAATAAAATAAGAAATAAATAAAA
TAAAAATAAAATAAAATAAAAATAAAATAAAAATAGAAATAAATAAAAATAAAATAAAATAA
AAATAAAAATAAAAATAAAATAAAATAAAAAAAATAAAAATAAATAAAAAATAAAATAAAAT
AAAATAAAAATAAATAAAATAAATAAATAAAATAAAAATAAAATAAATAAAATAAAAATAAA
ATAAAATAAAGCCAAGCACAACGGCTCACGCCTGTAATCCCAGACTTTGGGAGGCCGGGTGG
GCGGATCACCTGAGGTCAGGAGTTGGAGACCGGCCTGGGAAACATAGACAAGAAACTCCACA
TCTCTACTAAAAATACAAAAAATTAGCTGGGAGACCGTGGTGGTGGGACGGCCTTGTAATCC
CAGCTACTCGGGAGGCTGAGGCAGAAGAATCACTTGAACCCAGGAGGCTGAGGTGCAGTGAG
CCAAGATCGCACTGTTGCACTCCAGCCTGGGCAACAGAGGGGTGAGACTCCATCTCTAAAAT
AAATAAAAATAAATAAAATAAAGTGTTTGTTATCTGTACGTGGTAGCCCTCAAATGTTAACT
ATGAAGGTTTGTAGTTTTAAGATCATCAATTGATATCTTTTTGTAAAGTTTTTGTGGAGTTT
GTCTTATTATAAGTATGTATGTATTTTCCCCTTTGTTTTTAAATTGATAAAATTCTGTACCA
TGAATTCATTCTTTTACAGTGTATAATTCTGTACTTGTTAGTATATTCACAAGGTTGTGCAA
CCATCACCCTAATTCCAAACTTTTTGTTTTTATCATGCCCAAAGAAACCTCTATCTATCATT
GGTCTAACTTGTTCTGCCTCCTCCCGGTCTAAACTGTACTAATCCTACTTTCTGTCTCTATG
GGTTGCCAATTCTGGACATTTCATTAATGAATCATATGATATGTAGCTTTTGTGTTGAGCTT
CTTTTCACTTAGCATACTTTCAGTTCAACCATGCTGTACTTTATTCCTTTTTATAGCTGAAT
AATATTCCATTGTATGGATAAGATCCCATTTATTATCATTCGTAAAATAAAATTGTTTCCAC
TATTTTGTGTATAATTGCTATTATAATAGCACTATATTATGTCCTATATTATAATACTGCCT
GGCCGGACAGTGACTCATACCTATAATCCTATAGCACTTCAGGGAGGCCAGGGTGGTTGGAT
CCTCTGAGGTTGAGTTCGAGACCCAGCCTGGCCAACATAATAGAAACCCATTTCTACTAAAA
AAATACAAAAATTAGCCAGGTGTGATGGCAGGGCCTGAATCCCAACTACTTGGGAGGCTGAG
ACAGAGAAATTGCTTGAACCCAGGAGGTGGAGGGATTGCAGTGAGCCAAGGTCCACCACTTC
CACTCCAGCCTGGGGCGAAAGACTAAAGCTACAGTCTCAAAAAAAAAAAAATACTGCTATGGA
CATTACAATGTTTTCAGAAATCTTCCATTATAGACATGATTTCAATTCTCTTGGAAGTGGAA
TTCCTAGGAGCAGATTTGATGGATCATGGTAACTATGCTTAACATAAGCAACGCAAAATGTT
TTCCAAACAATTGCACTGTTTTATATTATTCTACATAAGCAATGTTTGAGGATTCTAACTGC
TCCGCATCATGGTTCCAATTGCCCACTTATTTATTGTCCATCTTTGTTATTCAGCCAACTTG
GTGGTGTGAAAATGAGTATCTCATTCGTCTTACATTGGTATTTCCCTAACTAGCACCATGTG
CTTATACTAGTCTTGATAGTATCCTTTCTGTACGCAGGTTTTTAATTTTTGATGTTTATTTT
CTGTTGCTTGTTTTGGATTATTCTGTGAAAACCTGCCTAATCAAGGTCTGAACTTATCACCT
CTCTTTTCTTCTAAAGTTTTGAGAATTTGGCTTACATTAGGTCTTCTACCCCATTTCAAGTT
AATGTTTGTGTGTAATGTGGGGTAGGAGTCTATGGCTTTCATTCTTTTGCATCTGTACTTTT
TCTCAGGTAGTGTCATTTATTCGAGAAAAAACCTATTCCTTCCCAATTAAAACTATCTAACT
TGTCCTTGTTGAAAACTGATATTAAAAAATGTAAGGTTTATTTCACAAAACCTCAAATTTTA
TTCTAACAATCTAGATGTCTATCCTATGCCAGTACCACAAGTAAGTAATTTGCACAACTTAC
AGCACTTTTGTAATAAGTTTTGAGAATGAAACAGTGAGTCCTCAAACTTGTTTCTCTTTTTT
TCCAAAACTGTTTTATATTATCGAGTTCCTTTCATATTTCCATATGACCACTCCAGAAGTGA
CACCCAATTTCTGCAAACTACTGATTTGATATCTAAAGCCACAAGTGTGATCATTTAAAGTG
GTATGACCCATATCTTGTAATATTAGAATCTTCTGTAATCAGTATCCATGAACCACGAGGAT
GTCTTTGCTTATATCTTTTAAATTTCTTTAATGATGTTTTGTTGTTTTTGGTATTTTAGTTT
TAAGATTTATTTTACTTGTTAATTTTATTAAACATGTATTTATTATTTTATACTATAACAAA
TAGGTGACAATTTTATTTTCAGATGACCAGATGTACATAAACATGACTTTATTATTGATCTT

GTATCCTGCAAACTTGATGACTCGTGTTATCTGACTCTAATTGCTTTCTTAGATTCCTTACT
ATTTTCTAAATATAGATCATGTCAGCTGCAAATGAGAGATAGTTTTATTCTTCTTTCCAATC
TAAATGACTTTCATTCCTTATGTTTGCCCTGATTTCGTTTGATTTATGACTTTCCAATTACA
ATGTTAACCAAATGGTGCGAAACAGAATCTCACATCATTTGTTCACAGTCTTGGGAGAAAAC
TACTAAGTCTTTATATAACTACACTGTTAGCTATCAATTTTTGTAGATACCTTTTATCAGCT
GCCGAGAAATTTCCTTCAACCCAAGTCTGCTGAGTGTTTTCTTTTTATCCTAAAGAGGCTGG
AATTTGTCAAGCATACTTTTTGTGTTTATTCAGATGATCCATAGTTTGTCCCTTTTTCACAA
TAATAGGTGCTTAATAAATTGATTTTTGTATGAAGAACCGCCTTACCGTTCCTTAAATAAAT
CCTACTTTTCCTGCTTTTCTCGTCAAAATTTCCTCTTCACTGAAGTTCAGGTTTGTTGAGTA
TTTTGTTCAGGACTGCAAAGTTTATTGTGGTTTTGTTTTTGTTCTGAGCCCAGGCTGGGGGT
GCGAACAGCATAATCACAGCTCACTACTGCCTCTCCCCAAACTCAGGTGATTCTCCCACTTC
AGCTTCCCAAGTAGCTGGGACACCGAACAACACCACATGCAACTAATTTGTTTTCTGTATTT
TTGGTAGAGATGGGGTTTGCGCAATGTTGCCCAGAGCTGGTGTTGAACTCCCGCAAACCTCC
ATTGATCCACTAGCCTGGGCCTCTTAAATTCTTTGAGATTCCAAGCTGGGGACTCACCGGCT
ACAAAAGTTTACTAAAACATAGAAAAACTACCCACCATATGTTGTACTATGTATCACTATAT
GCTGTCATTCAGAAATTTATATACATATCTTATAAAACATAGAATTATATGCAACAAGTGGG
ATAATGGTGACATCTTGGAAAAATAAGAAGTATAACTGGAAAAAGGTACAGAGAACCCTAAG
TATACTTGTAATATCTTATTTCTTAACATTAACTGTACGCTGTGGCAGCTACGTCATTTATT
ATTTAACTGTGCTTTTTATATCTGAAACATTTTACAATGACAATTTTTTAAACTTGGCTTGT
ATTTTCCCATAACCTCTTCTTTTGAATAAGGGGTCACTATGGTAGCCGAAACTGACTCAAAT
TACAAGACTGAGAGTCTCTTTCACTTCAGCCTCCCAAGTATAACAGTGCTATAACACCAGCT
CTCCGTGCCCTCCACACTCACGCCCTACCCCTACCTTATTTAAAGAAAGGTTACAGATAAGA
AACAAAAATTGCTGGCTGGCTGTCTTGCCACACTCTACATTCAAGACTGAAGGGGGCCAAAT
TACTGCTACACTGTCACAAATGCCTTTCACCTAACTTAAAAAACCACAAAGGTAACTGCTGA
CAGAGTTAACCATACACACTGATTTTTATTCCTTTACAGGCATATTACACTATAATTAATCT
ATCTTTATATCTCTAACTCCTTACTATAAACATAGAATTCCTTAATAGCCCAAGAATCAGTC
TTTTTCATATTTGTGGTTCCAGCTCCTGTATGTCCGCGTGCAAGCATTACATGTCCTGGTAA
ACGTACATACATTATGAATAAAATTTTGGTAAATCAAGAAATAATAAAATGAAAATGAGCCT
CGTAATCGAATTTTTATTAAAAAGTTGCACGTAGTAAGGCCTGGGGCAGTGGTTCACGCCTG
ATACTCAGCATTTGGGAGGCTAAGGTGGGCAGATCACTTGAGCCCAGGACTTCAAGACCAGC
CTGGGCAACATAAGAAACTCCACCTCTAAAAAAAAAAAAAAAAATTACACAGCAAATAGCTGG
GCGTGGTGGCATGCAGCTGTAGTCCCAGCTACTGAGGGAGAGCAGAGGTGGAGAATCACCTG
AGCCCTGGAGTCGGGACTGTGGCGAGGCGAAATCATGCCACTGCCTCCAGCCTGTAACAGAG
TGAAACTCAAAAAAAAATTGGCCAGGTTAGCGGCTCACACCTGTAATCCCAGCACTACAAAA
AAATTAGCGGGCATGGTGGCCTGCGCCTGTGATCAGCTACTCAGCAGGCTGGGGCACAAGTC
CTGTTGAACCGGAGATGGGGTTGCAGTGAGTTGGTCGTGCCACTGCACTCCAGCCTGGGTGA
AATTGAAACTGTCTCAAAAATATATATATATTGCATTAATATATTGCTGCAAAGCCAAAAAT
TCAAAGGTATAGTGTCAATCCAAGTTAAAGTTTGAAACCGAATTCAAAATAATGGGTAAGTC
TAAGATTTGTGTTCTAGTCCTGGCTCTACAATGACAGCTGTGTGATCTATTTTACCTATTTA
AGTGAGGACGTTATTTCTTGAAGTCCTAGTTTCTAAAATTGAATTTGCTGAATTTTTAAGCT
TTGGACTTGGATGAAAGAAATAACATATAAGGGAATATTTGTCTTTTGAGCTGCACAGAATC
TTTCTATTTGGAAAGAACCCAAATAGGTGAATGCAGCCTCCCGCCCACAAGCTTAGTTAAAG
CACAGCTCTAGGTATTTAGTGAGACAAAGGAACTTCTAGAAGGGGCTTTGCATCTTGAAGAG
AAATAACACAAAGGCACAAGGGGATCTTGAGGGAGCTGGGGTCCAGGTACAAGCCAATCTGG
GGGAAAGAATGCAGCAACACTACTCTAAGTAGATTATTCTGCTGCAGGGTCTTGGTCAACCT
GTTACCCTGGCCTTGTCAATACTGAGTGACCTAATACCGTTTTGATTAATTCCTTTTTGGCT
TAATTAGCAAGACCGGTTTCTTTGTTACAACCAAGAACCCTGACCAGCTGCAAAACCATGAA
AGCAGAGGAACCTTAGGATAGGCATTTAGATGTTGCAGAAGAGAGCAAAAGCAAAACCGCAT
AGCTAGATTTGCTCGAAGAACAATCAGGCGCAGACGCCGATTACAGTTTTGAGGTCTATAAA
GCATCTTCAACAATAACTAAGCACAAACTCTTAGGTGTCATTCTTTAAAAAATTTTTTTCTT
CATTGCCCAAGCTGACCTCAAATTCCTGGGCTCAAGTGATCCTCAAAGCCTCCCAAGTACAG

GCACATGCCACAACACCTGGCCAGTTATCATTCTTGCCACATAATAAGGGAGCATGTTTCCA
CTGGTTGGAATGTCATTACTAAAACTTGTCAGAGGCTCATAAAGTAATTACACTAGTTAATA
AAGTTTTAAGAAAATTATTAGCTATAGGCAACATTTTTCATGACCTTCTAGAATCAAGGTGG
TTCAGGCATCTGACCCACTGCTTAAATCAAGCTCTCTATATAATTAAGGGTTACTAGGTGGC
TTTGGCTAAAATTATGAAAAGGGAATGAAATGTCTTGTGGAGACACAGTATGAATGATAGAG
CAAGACTGCTTCACGAAAATGCAATAAAGTAGTCAAGTTATTTTTTCCCAAGGTTTAGGAAT
CTAAGAAGGGTCTGAACTTCTAAATGCTAACATGACAAGACCAATTAACATACAGGAGCACA
AAGTCATTCACTAACATATATAACAGGAAAAGGGAAAAACCTCTCATGACACATCTCTTTAG
GCTGCTGTACCCAATCTTGTTTGGATGGGATGGGTGGCTAAACCTTCATCTTAAGAGACTTA
AGAGAGGACAGGGACAAAAGGAATTATTCCCAAAATGGTAAAAATCAGCAGATTGAAAGGAT
CAAGAAAAGAGAGACCATGCCTTTCTTTCTCCAAAGCGAAGAAATCATTTTCAATATCTGAA
AAAGGCTGGACCGGAAAGCTATAGAAACTGAAAACAAACAAACCATGAAAACAAAGTAAGTG
AAACAACATTTGGTTCATCTGGGGGTCCCTGCCCTCAGCATGAACTTGGTGTAAGGCTCCAA
AATTACTTAAATGGAAACCATCACTACAATTAGGAATTTCAGTCCAGACAACAGCCTCACTT
CCCTTACATAATTCTCCTCTCTTCAAATTCAAGTTATTGTCAAGTCAGGTTCTACTGATACG
GGACACACCTAGAAATAAAAGGTATAGAGGTCAGGCGCGGTGGCTTGTGCCTGTAATCCCAG
CACTTTGGGGGAGGCCAAGGCAGGCGGATCACTTGAGATCAGGTTGGAGATCAGCCTGGCCA
AGCACAGGTGAAACCTTCTCTTCACAAAATAAACACACACACAAAATTAGGTGGGCGTGGTG
GGGCATGCCTGTGTCCCAGCTACTTGAGAGGCTGAGGCAGGAGAACTGCCCGAAACCAGGAG
GGTGGATGCTTCAGTAAGCCAGATCACACCGCTGCACTCCCAGCCTAGGCAACAGAACAAGA
CTCCGCCACCCCAAAGAAAGGAACCTTAAAACCAGGCGCCTGGCTCTCAACACCCAAGAGAG
CCAGGCGTGAGGACTGTTTTGAGGTCAAGGAGCAGGAGTTCGAGACCAGCCTAGCCAACCAT
GGGTGAATCCCGATCTCCACTAAAAATACAAAAATTAAGCAGGCGTGGTGGCACCCCGCTGT
AGACCCAGCCACTATGCTGAGCTGAGAGCCTGAGAAATAGCTGGAACCTACGGAGGCGGGGC
TGCGGGTGAGGTGAGGAGATCACACCACTGCACTCCAGCCTGGCGAATGGAGAGAGAGAGGG
GAGAGGTATGTGTAAGAAAAAGGAGGTCTGGGCGTGATGTCTCACTGCCACTGCGTAATCCC
AACTCTTTGGGAGGCCGAGACAGGCGGATCACGAGGTCAAGAGCTGGAGACCAGCCTGTCCA
GCCAATACAAAGCGAAGAAAAAAAAAA

**#Read from Family 5; III-37**

>276436a0-b04f-4edf-b4b5-bcd1baccb342
runid=dc02366810d2b802a745c669295f0b5aa972cc39
sampleid=LLAAA044274 read=14872 ch=93 start_time=2018-04-13T16:23:32Z
CAGTGTACTTCGTTCAGTTACGTATTGCTACTAAGTGACCAGCTCCCAATAAAAATTTTTGG
GTGTTAAGAGCCCAGGCATGGTGGCTCATGCCTAATCAGCACGCTTTTTGGGAGAGCCGAGG
CAGGCGGATCACCTGAGGTCAAAGTTCAAGACAAACCTGGCCAACATGGTGAAACCCCATCT
CTGCAAATACAAAAATTAACCCGACCATACTGGCACACACCTGTAGTCCCAGCTACTCGGGA
GGCTGAGGCACAAAATTGCTTGAACCGGCAGGGTTGCAGTGTGGGCCAAGATCTAGCCACTG
CATTCCAGCCTGAAACAGAGGCGAGACTCCGTCTCAAAAATAAATAAATAAATAAATAAATT
GGTTGTTGGCTGGGCGTGGTGGCTCATGCCTTATAATCCCAGCACTTTGGGAGGGCCAAGGC
AGGCCCGGGTGCACTTGAGGACAAGCCGAGACCAGCCTGGCCAACATGGTGAAATCCCATCT
CTACTGAAAGTTAAAATTAGCTGGGCGTGGTGGTACACGCCTGTAATCCCAGTTACTTGAGT
CTGAGGCAGGAAGATCGTTTGAACCCGGAGAGCAGAGGTTACACATGTTGGGATCCAGTTTC
ACTGTACTCCAGCCTGGGTGACAGACCAAGACTTTTTTTTTTTTTTTTTTTTTTTTTTTGAG
ACAGAGTTTCGTTCTTGTTGCCCAGGATGGAGGGTGGCAATCTCGGCTCACCTCCTCCTGGG
ATTCAGTGATTCTGCCTCCCAGCCTTTGAGTTGAGCATGCCTATAATGCCCAGCTAATTTTG
TATTTTCAGTAGGGCTTCTCAGGCTGGTCTCGACTCCCGACCTCAAGTGATCCACCACCTTG
GCCTCCCAAAGTGCTGGGATTACAAGCGTGCCACTGCACCCAGCCAAGACTCGTCTTTAAAA
AGCACTTTGGAGCTGGGTGTGGTGGCTCACGTCTGTCTCACGCCACACACCTGGGCACCTGA
ATCAGGAGTTCTCCACCCGCAACATGAATAAACCCTGTCTCTACTGAAAATACAGCTTAGCC

AGGTGTGGTGGCACACACCAATAATCCCAGCTACTCGGGAGGCTGTGAGAGAACTGCTTGAA
CCGGGAGGCGGAGATTGCAGTGTGGAGCCAAGATCACGCCATCGCACTCCAGCCTGGGCAAC
AAAATGAAAACTCATCTCAAGAAAAACAAAACAAAACAAACAAAAACAAAAAAACAAAAAAA
ACTTACTTTGGTGTTTTTTTCTTACGAGACAAGTCTCACTCTGTTTACTGATCTGGAGTGCA
GTGATCTCGACTCAGTACAGCCTCCACCTCCTGGGCTCAAGCAGTCATCCACCTCAACCTCC
TGTGTAGCAGGGACTACAGGCATGCACTACCACCACAACTAATTTTTTTATTTTTTGCAGAG
ACTGGGTCTTCCTGTGTTGCAGGCTGATCTCAAACTCCTTGTTAAAGAGATCCTCCTGCACT
CAGCCTCCCAAAGTGCTGCGATTACAGGCACATGAGCTACCGTGCTCCAGCCAAGGTGTTAA
ATTTCTAATGGCCTTCCTAAACAGAAACGTGCACACATATTGCTACACATTTTCACTGCTGG
GAAGGCTTCACATGGACTCCTCCAGACTCCTCTGTGCCTTTTCTCTTATGATCCAGCTGCAT
ATCTTTACTCCTGAACACTGTCATAAATCTTAGCTGGGGCTGGGCACGGTGGCTCATGCCTG
TAATCCCAGCACTCAACAACCGAGCGGGCGGATCACTGCAATCAGGAAGTTCGAGTGCTGCA
ACCCATGGTGAGAAACGTCTCCTAACAAACACAAAAAATCAGGCGTGGTGGTGCACCGTGTA
ATCCCAGCTACTTGGGGCTGAAGCAGGAAATTGCTTGAACCTGGCAGGAGATGGAGGTTACC
TGGTGAGAAGCCAGATGCACCGCCAGCCTGCTGAAACGAGATCCGTCTCAAAAAAAAAAAAA
AAAATCAATATTTTTGTGCACAGTATGCACATCCTGAAGACAAAGACCCTGGGAAATTTTAT
TCTTCGTATTGGTGATCTTACTGTATTGTTGTTCTGAGCTGTGTATTCAACAAATTAGTAGT
TGTGTTGGGGTTTCACAGAATGGATTCTATGAAACAGGAAAAACAAGGTATAAAGATAAGAA
ATAAACTCCTGAATCTGAGTTTGAGTCAAAATATTAATATCATCTTAGGATTATTTTATCTT
AAAAATAACCATACCAAATAAAATAACATGATACTTGGTTCTGTCTTCTGAAAGACTACAAA
CAATAATTGACTAATAGCTATGAATATTCTTAGCAGCCAAATTGTGTTATTTAAATGCCATT
TTCCGCTCCCTAAAGAAATGTTAATTTCAGGTGTAAAACTAAAAATATACAAGATGAACCTA
CAACATCTTGTAACACCAGAAAAGCAAGACAGCTTCTGGGGTCATCAAAAGGAGCGAGCCAA
CAAGAAAACTCCCAATGACCAAAACTGGAATCAATTTGAGCATCAATAGGATAATATATTTA
AAACTAAGTACATTTAAGTCTGTGTCTTTTTTTTTTTTTTTTTGAGACGAGTCTCACTGTGT
CGCCACGCTAGGTACAGCTGACACGATCTTGGCTCACTGCAACCTCTGCCTCCCACAGGTTC
AGAAGCTGCATTCTCTCTCCTGCCTCAGCCTCCCAAGTGGCTGGGATTACAACACGTGCCAC
AATGCCTGGATAATTTTGTACTTTAGTAGAGACAGGGTTTCACCATGTTGGCCAGGCTGGTC
TCAAAATCCTGACCTCAAGTTATCTACATGCCTCGGCCTCCCAAAGTGCTGGGATTACAGAG
CGTGAGCCACTGCGCCTGGCTTTTTGTTTTTATTTTTTTTTGAAGGGGTCTCAAACTGTTGC
CCAGGCTGGCACATGCAGTGGCACAAACACGGCTTAATGCAGCCTTGACCTCCTGGACTCAA
GTGATTCTCCCAACCTCAGCTTCCCAAGCAGCTGGGACTACAGGCACATATCACCATGCCTG
GCTAATTTTTTATTTTTGTAGAGACAGGTCTCTCCGCTGCCTGGCCCAGAGCTTTGTTTTAC
AGGTGTTCTTACTTGGGCTGGGCACAGTGTGGCTCACGCCTCCTAATCCAGCCTTTTTGGGA
GGCTGAGGTGGGAGGATGCTGAGTCAGAGACTGGGCTCAGCCTGCGCAGCATAGTTGAGACC
CCATCTCTACAAAAAAATACAAAAAAATTAGCCTGACATGTTGGCTTCTGCATACAATCCCA
GCTCCTCAGGAGGCTGAGGCAGGAGGATCACCTGAGCCCAGGGGGAGAGAGTCAAGGCTGCA
GTGAGTTGTGATTGTACCACTGCACACCGCCTGGAATGGCACAGTGGGAGGCCTGTTTCCAA
AAAAACCCAAAATCCTTATTTGAGATAAATAACTGAAATATATAATAGAAGAACTGGGTTCT
CCTACAGTAAAGCTAGAATTTGTAAAATAATCGGGGAGTGAAAGGGAATGGGAAAGGGGTAT
AGATAAAACAATTTTGGCCATAAGTTGATAACTGGAGCTGGGGATGGATACAGGGGTTCATT
ATACTCTTCTACTTCTGTATCTTTGTATCTATTTCAAATTTTCCATGATAAAACCCCCTACC
TTGGCTCAAGATCTAATTCCCATTTGTGGAAATCAAACTCAGCTACATGGTTTTAGTGCCCT
CTGTACACAGTCAATGCAATTTATCCTTCTCCTAACCTCTACTCCTCTCTAAGCATGAGTCT
CTCAGTGCATGCCTGTCATTCCTATCTTTGTCTTTGCTTGAATCATGTCTTCTAACCCTAGT
GCCCTGCCTGCTCTCTCTATCAATTGTACTTTTTTTGTTTTTTTACAAGGACCAACTCAAGT
CCTTGAGGTGGTATAAATAAAACAACTTGATGAAAGAAAACTACACTAAAGAACACTGTCCA
TATAGCCTAAATCGAACACAAAAAACAGCCAGGGAGAAACAAAGCCAAATTATAAGGTGAAG
TGGAACTAAAACCAGCAACCACTAGCATATCTATATACTTTCTCAGTGGAGATTTAGCTTAA
AAAGATAAAAATAAACACAAAACACCTCATTGGGCTGAAATAAATATTCTTTTCAAGAAAGG
TCACAGAATTAAAGAAGGCAGACAGCATGATCCAATCTTCCGTACTCTGGCAAGGAAGCAGA

AATCAGCTTCCATCCAGTGCTCTGCCACTGTAGTAAAATGTTGACAGAAGTACTCGCTCTAG
TTCCTGAAGAATGCTACTGATACAGGCTAATGCGTCAGTGGCTATGATTCTTTAACTATCTC
TCAGATAAGAGGGAACCAATGGATTCATACTACTTTGTCCTTGAGGAACTTCAGGTACATCA
CCTCGATCAGGAGTTTAAAAAATGGTTCCCAGACCAGTAGCATCAGATATTTGGCGAGAACT
GTTCCTGCGCCACCCCTCCTCCTAGTTCAGGTAAGACATCCTTGAGAAAAATTCCACAGCCA
TGACATGTTATCTCTTGGATGAGGTAAAACTCACCTCATCAAATGACTTGTGGGACGGATGT
ATTGGGATTCATATGATCTGACCTAATTCTGGAGACTCTGGCACTCGGTGATGCTCCACAAC
ATAGTAGGAGAAGAAGCAAACTCTTAGTAATGTCTATATAAAACAACAGGAGAGCTAAGTTA
AGGGAGGCCTAAAAAGCTGGCTCTGATATTTTTCAATGCACAAAAACAAAGGGAGTCACTAA
GATGAATAAATACAAACTAAGTTCTAAAAATGAAGCCGAAGGCCAGACGTGGTGGCTCATGC
CTGTAATCCCAACACTACAGGAGAGCCAAGGAAGGAAGATCACTTGAGGCCAGGAGTCAAGA
CCAGCCTGGACCACTGTCAAAAACCCTAGTTACAATAAACAAATGAATTAATTAAAAATGAA
GTGAGTTCCTTTGGTAATTTACGGCTCACTAGGCTGTGGGTCAAAACAACAGCACTTCTTTT
GATTACCAACATTAAAATTAAAAAAAAACAGTTTAACTAAGCTTGTATCTCTCCATTCTTTC
TAGACAGTCAAGTCCCTCATCTTTAGCTGGATGGAAGGGGGCACTGGCTGCAAGGGAAAAGC
CTGTGGGAGACTCATGAGACACTAGAATATGTACAAACTCAGTGCACGTAGGAGAATGAAGG
CCACAGCTAAAGGAACTGAGCAGCAGCCACGGGCAGCCATACATACCCGCTGACACCAACAC
CATCATGTTGTTTTCCTGATCCACACTATACCGCCCGAACATAAACATAATCCGTGAGTACA
TTGGATACTAAAGAAATGGAGGGCAGGATTAGTGTTCTGCATCACACAAACCTGGACAGAAA
TAAAATCAGAAGTAAAAATACTCACAGAAAATGGAATTACCCAGTGAAGAACCTCGGAACCA
CTAACCACATCCCTCTCTCTCGATCACCTCCAGCTTGATTACCAGGGCATCCCATTTTTTTC
TATACTCTGTGTCCAGCTGCAAACTATAGATACAAAGACAAGCTCATCTGAAAAGCTTTCAG
ATACTTTGGTCTCTCTGATCAAATCTTATTACAAGGAAAGTAATCTGACCTAACAGACTAGT
GCCAGGAGTGAGACATGGCTAAGTATTTTACTACAGATGACAACAAAATCACCTTATACAGG
CTGTGTCATTACACTGAATGTGAGATCTTCATTAGACAAACCAAGGAAAGATGTAACAAAAT
ATTTTAATAGACACATCTGGGGAAAATAATATATGGTGATATGAATGTGTAAGAGATAAGAC
AATTCTAAAGTTTTAAACAACAAAGGATCAAAAGTGTTAAGTAGACCTTCACACCTAAAAAT
GCAAGCAAAACATATTTTTTTAAGGCTACCGAAAATCACTGGTGGCTGAAGATTACTCAACA
AGACAGATATACCATATTCTCTGGCTCCAACAAAGTCATTCCCTAAATTAACAAGATCAGTA
CCTCAGGAGAGGATTGCACTTGCGATTAGTGGGTGATAATAGCGAAAGTGGTTAGCGGAGTC
AATAAAAAACAAATGAATCAGAAAAAAATTTGTTCTAGAACTCTGGGCGATCCCACCTCCCTG
TTTCATTTTTCGCTCCATCTTCCTAAGGAGGAAGCAGTCTTTGAACAGATACTCTTCATCTT
TATGAAATAAACATTAGACCAGCCTGAGTGACTCACACCTGTAATCAGCACTGGGAGAACAA
AAACAGGAGAGGATCACTAGAGGCCCAGGGTTCAAGACCAGCCCCTGGGAGACATAGGAGAC
CCTGTCTCTAAGAAAAAAAAAAAAAAATTTTAATTAATAGGGTGTGGTGGCGCATGCCTGTGT
AGGCTGCGCGAGGTGGGATGGTCAGGAAACAAAGGCTGCAGCAAGACATGATCACACCACTG
TACTCCAGCCTGGGTAACACAGCGGGACCCTGTCTCAGGGAGAGGAAAAAAAAAAACCCTCC
ACTATGGAAATATTCTTGATTGAGGGTTAAACAGATGTGACAACTAAATATAATCTATCTGT
ACTGAAGTGGAAAATGCTATAAGGAGGCTTGATGAGTCAACTGATAAAACTGATAAATACAA
ACAGAGGACTACATGAAAGTGTTCCATCGGTGTTAAATTTACTAATTCAATAATCGTGATTA
GTTAAGAAATACGGCCATATAGCTAGTTTTAGGAAATACACACTGGAAGAATTTGGGAGTAA
GGACTATGATGTATGTAACTTACTCTTAAGTTGTTTGAAAAAGCAATAAAGCCCTCATAAAA
TACATTAAAGTATTCATATCCATTCCTCTATCTGATATTCCCAACAGTCCTGTGGGAGGTAA
AACGATCTCTCATCCCCATGGGACAGATAAAGAAATTCTAAGTCAATGTTTACCTAAATGCT
ATGCAATGTGGATGACAGGTAGATAGATGGATACTTAAGCCAATGTCACCTGAGTCAGATAA
ATGATTCGTAGACAGTAGCAATTGATATGCTGGAGGCTCCAAATCTGGAGAAAAAAATTAAC
TCACCTGAACATTGAAGAACTGCCGAGGTGTCACATCTTGTGTAGGTTCCAAAAACTAAGAA
TGAAAGAAAGAAATAAGGAGATGCTGGCCCCCTTATAGTGTACAAATCTTTCGCCTGTTTCT
TTCACCTAGCGGCGTTCAGATCCTAAAGTACTTTAAGAGATCTTAAAGGCAGGTTCAGAAAC
TTAAGTCTTAGTAGAGCAACAAAGAAAAGGTAAAGAGGATGTAAGCAAAGACAACACTGAGA
CATTTGTCATCTGGGAGAAGAAAAAAACAATAAACAAAACTACGGCAAATTTTCTGAAAGTA

GAACAGTTTAGAGAAGTATGAAGAACAGAAATCCCCATGCACCTGTGCAACTATGGAACAAA
AGATAGCCACACTAGGCTCACCTCGGTGCAGTAAAGGTGGGTGCCTGTAGTGAGCCGCCACA
GCTTAAAGTGTTTTCTTATCCATCACCATTTCCCAAGGTTGCTCTTTGCCCTCTGAATCTTC
ATTCCCTTCTGTTTGGGCTTTTGGTTCTGGAGAGTGGTGGACTCAGAGCTCTGAAACATATT
TGACATTTCACCAACCGCTTCATCTCATTAATAGATCTGTAAAAGGGAAAAAGAGCCATGGT
GAGAGGTGGTTAGTCAGCCCTGTGAAATGAAGTGTCCACACAAGGTCTGTGCAAGAAGGTTA
TACAGTAAACCACAGTATGTAGTATAAACAGGACACAATATAGTCAATAAAAACTGAGTTCC
TGAGTTTGAGATCTTAGTCCAACACTCACTAACTAGATTATTATTTAACCAGTTCCTATTTC
TAAATCTATAAAATGGAAATAATAATGGTACCTATCTCGAGGTTGGCTATAAAGATTACAAA
GTGTCAAGTGCTGCAGGGGCGTGGTGGCTCACGACTGTAATCTCAGCACTGGGGAGAGCAGA
AAGGGTGGATCCTCAGGAAAGCTCAAAACCAGCCTGATCAACATGGTGAAACCCTGTCTCTA
CTACAAACACAAAAAGTGTTAACCGGGCATGAGGTGAGCGGCGCAACTGTAATCCCAGCTAC
TCAGGAGGCCCGAGGCAGAATGACTTGAACCTGGGAGGCGGGGTTGCGTCAAGCCGAGATCA
CGCCACTGCACTCCTAGCCTGGGCGACAGAATTGAACTCTTGTCTCAAAAAAAAACAAAAAA
CAAACAACGAAAAAAGTAGGGTGTCAGGAGTGCTTAATATATCTATGGCCAGGCCTGAGGCT
CACTCCTGTAGTCCCAGCACTTTGGGAGGCCAAGGCTCGGGAGGAGGACTTTCCCTTGAGGC
CAGGTTCAAGATAACCTGGAAACACAGTCGAGACCCTACCTCTAAGAAAAATTTAAAAAATT
AGCCAGACATAGTGGCACATGCCTGTACTCAAGCTATAGGCTGAGATGGGAACATCACTTGA
GCCCAGGAGTTGGAGGCTGTAGTGAGCTACAATTCTGCCACTGTACTCAGTCACAGGCAATA
CAGTAAGACTCTGTCTCTTTTTTTGTTTGTTTTTGAAACAGAGTCTCACTCTGTCACCCAAG
GCTAGAATAGAGTGCCATGATCTGGGCTCACTGCAGCTACGCCTCCTGGGTTCAAGTATTTC
TCCTGCCTCAGCCTCCCAGTAGCTGGAGACTACAGGCCGCAGCTATCATGCCCGGCTGATTT
TTGTATTTAGCAGAGACAGGGTTTCACCGTGTTACCCAGGGCGGTCTCAATCTCCTGACCTC
ATGATCTGCCCGCCTCGGCCTCCAAAGTTCTGGGATTACAGGCGTAAACCACCGATGCCCAG
CTACACTGTCTCTTAAAAAATAAAATAGGCTAGGCGCAGTGGCTCACGCCTGTGAATCCTAA
TACTGGAGGCTTTGGGCTGGCAATCACCTGAGGTGTCAGGGTTTGGCAGCTAGCTGCGTGGA
GAAACCTGTCTCTGCTAAAAATACCAAAATTAACTGAACACTGGTGGCTTATACCCATATAA
TCCCAGCTGCCTCAGTGAGACAAAGTAACAGGCTGAAGGCAGTACAAGGGACATTATAGCAC
AGACCAACAAGCGAAAACTCCGTCTCAAATGAAATGAAATGAAATGAAATGAAATGAAATGA
AATAAATGAAATAAATGGTAAAGAAATGAAATAAATGAAATATAAGAAATGAAATAAAAATG
AAAATGAAATAATGAAAATGAAATGAAATGAAATAAATGAAGTAAAGAAATGAAATGTGAAA
TGAAATGAAATGAAATGAAATGAAATATGAAATGAAATGAAATGAAATGAAAGAAATGAAGT
AAATGAAATAAATGAAATAAAAATGAAATAAAGAAATAAAGAAATGAAATAAATGAAAATGA
AAATGAAATAGTAAATGAAAATAAGAAATGAAATGAAATGAAATGAAATGAAATGAAATGAA
ATGAAAATAAGAAATGAAATGAAATGAAATGAAATGAAATGAAATGAAATGAGAAATAAAGA
AATGAAATAAATGAAAGTAAATGAAATGAGAAATAGAGAAAGAAATGAAAATGAAAGTAAGA
AATGAAATGAGAAATGAAATGAAATATGAAATGAAAATGAAATGAAATAAATGAAATGAAAT
GAAATGAAATGAATGAAATGAAAGTAATGAAAGTAAATGAAATGAAATGAAATGAAATGAAG
AAATAAATAAAGAAATAAAGAAATGAAAATGAAATGAAATAAGAAATGAAATAAGTAAAAAT
AAATAAGAGAAATAAGAAATAAATAAATAAATGAAGTAAGTAAATGAAAATGAAAGAAATGA
AGTAAATGAAATGAAATGAAATGAAATGAAATGAAATGAAATGAAAATGAAGTAAATGAAAT
GAAATGAAGTAAATGAAATGAAATGAGAAATGAGAAATGAAATGAAATGAAATGAAATGAAA
TGAGAAATGAAATGAAAATGAAATGAAATGAAGTAAATGAAATGAAATGAAATGAAATGAAA
TGAAATGAAATGAGAAATAAAGAAATATGAAATGAAAGTAAAGAAATAAGAAATAGAAATGA
AATGAAATGAAAGTAAATAAATATAGAGAAATAAAAATAAAGAAATGAAATAAAGAAATGAA
ATAAAGTGAAATGAAATAATGAAATGAAATGAAATGAAATAGAAATGAAATGAAATGAAATG
AAATATGAAATGAAATGAAATGAAATGAAATAAATAAATGAAATGAAATAAAAATAAGAAAT
GAAATGAAATGAAATGAAATAAAGAAATGAAGTAAAATGAAATGAAATGAAATGAAATGAAA
TGAAATAAATAATCAAAGCATGAAATGAAATGAAATGAAATGAAATGAAATGAAATGAAATA
ATGAAATGAAATGAAATGAAATGAAATGAAATGAAATGAAATGAAATGAAAATGAAATGAAA
TGAAATGAAATGAAATGAAATGAAATGAGAAATGAAATGAAATGAAATGAGAAATGAAATGA

AATGAAAATGAAAATAAATGAAATGAAATGAAATGAAATGAAATGAAATGAGAAATGAAATA
AAATGAAATGAAATGAAAATGAAATGAAATGAAATGAAATGAAATGAGAAATGAAATGAAAA
TAAATGAAATGAAATGAAAATAAGAAAATAAAGAAATAAGAAATAGTAAAAATGAAAGTAAA
TGAAATAAGAAATGAAATGAAATGAAATGAAATGAAATGAAATGAAATGAAATGAAAACAGA
AATAAAAATGAAATAAATAAATGAAAATGAAATGAAATAAATGAAATGAAATGAAATGAAAT
GAAATGAAATGAAATAGAAATGAAGTAAATGAAATGAAATGAAATGAGAAATGAAATAGAAA
TGAAATAGTAAATGAGAAATGAAATGAAATGAAATGAAATGAAATGAAAATGAAATGAAATA
TGAAAATGAGAAATGAAATGAAAATGAAATGAAATGAAATGAAAATGAAATGAAATGAAATG
AAATGAAATGAAATGAAATAGAAATGAAATGAAATGAAATGAAATAAATAAGAGAAATAAAG
AAATAAGAGAAATGAAATGAAAATGAAATGAAATGAAATGAAATGAAATGAAATGAAGAAAT
GAAATGAAAGCAAGAGAAATAAAGAAGTAAAATAAATGAAAAATGAAAATGAAATGAAATGA
AATATGAAATGAAATGAAATGAAATAAGAAATAAATAAGTAAATGAAATGAAAATAAAGAAA
TAAAAATGAAATGAAAGTAAATAAATAAATGAAATGAAATGAAATGAAATGAAATGAAATGA
AATGAAATGAAATGAAGTAATGAAGTAGTAAATGAAATGAAATGTAAATAAAAATGAAATGA
AAATGAAAATGAAATGAAATGAGAAAATAGTAAGAAATGAAATGTGAAATGAAATGAAATGA
AATGAAGTAAATGAAAATGAGAAATGAAATGAAATAAGAATGAAAATGAAATGAAATGAAAT
AAAGAAATAAATGAAATAGTAAATGAGAAATGAAATGAAAATGAAATAAATAAATGAAATAA
GAGAAATGAGAAATAATGAAGAAATGAAATGAAATAAAGAAATGAAATGAAAATGAAAATGA
AATGAAATGAAATGAAATGAAATGAAATGAAATGAAAGTAAGAAGTAAATGAAATAAATGAA
ATGAAATGAAATGAAATGAGAAATGAAATGAAATGAAAATGAAATGAAATAAAGTAAAAATG
AAATGAAATGAAATAAAGAAAGTAAATAAATGAGAAATGAAATAGTAAATGAAGAAATGAGA
AAGAAATAAAGAAATAAATAAGAAAATAAGAAATAAAGAAATAAATAAAAATAAGAAAATGA
AATAAGAAATGAAATGAAATGAAATGAAATGAAAAATGAAAATGAGAAATGAAATGAAATGA
AATGAAATGAAATGAAATAAATGAAAATGAAATGAAATGAGAAATGAAATGAAGAAATGAAA
ATGAAGTAAGAAATGAAATGAAATGAAAATGAAAATGTGAAATGAAAATGAAATGAAATAAA
AAAATAAAATAAAATAAAATAAAAATAAAATAAAATAAAAATAAAATAAAATAAAATAAAAT
AAAATAAAAATAAAATAAAATAAAATAAAATAAAATAAAATAAAATAAAATAAAATAAAATA
AAATAAAATAAAATAAAATAAAATAAAATAAAAATAAAATAAAAATAAAAAATAAAATAAAA
AATAAAATAAAATAAAAATAAAAATAAAATAAAATAAAATAAAATAAAATAAAATAAAAATA
AAATAAAATAAAATAAAATAAAATAAAATAAAAATAAAATAAAATAAATAAAAATAAAATAA
GTAAAATAAAATAAAATAAAATAAAATAAAATAAAATAAAAATAAAATAAAATAAAAAATAA
AAATAAAATAAAATGAAATAAAATAAAAATAAAATAAAATAAAATAAAATAAAATAAAATAA
AAAATAAAATAAAATAAAACAAAATAAAATAAATAAAATAAAAATAAAATAAAATAAAAATA
AAATAAAATAAAATAAAATAAAATAAAAATAAAATAAAATAAAATAAAATAAAATAAAGAAA
CAAAATAAAATAAAAATAAAATAAATAAAATAAAATAAAAATAAAATAAAATAAAATAAAAT
AAAATAAAAAATAAAATAAAATAAAATAAAATAAAATAAAAATAAAATAAAATAAAAAATAA
AAATAAAATAAAATAAAAATAAAATAAAATAAAATAAAATAAAAATAAAATAAAAAATAAAA
TAAAATAAAAATAAAATAAAATAAAATAAAATAAAATAAAAATAAAAATAAAAATAAAATAA
AATAAAATAAAAATAAAATAAAAATAAAAAATAAATAAATAAAATAAAATAAAAATAAAATA
AAATAAAATAAAAATAAAATAAAAATAAAAATAAAAATAAAATAAAATAAAATAAAAATAAA
ATAAATAAAATAAAATAAAATAAGAAATAAAATAAAAATAAAATAAAATAAAATAAAATAAA
ATAAAATAAAATAAAATAAAATAAAATAAAATAAAATAAAATAAAATAAAATAAAATAAAAT
AAAATAAAATAAAATAAAATAAAAATAAAATAAAAATAAATAAAATAAAATAAAATAAAATA
AAATAAAATAAAATAAAATAAAATAAAAAATAAAATAAAATAAAATAAAATAAAATAAAATA
AAATAAAAAATAAAATAAAATAAAATAAAATAAAATAAAATAAAATAAAATAAAATAAAAAA
TAAAAATAAAATAAAAATAAATAAAATAAAAATAAAAATAAAAATAAAAATAAAATAAAATA
AAAATAAAATAAAATAAAAAATAAAAATAAAATAAAATAAAAATAAAATAAAAATAAAAATA
AAATAAAATAAAAAATAAAATAAAATAAAAATAAAATAAAATAAAATAAAATAAAAAATAAA
ATAAAATAAAATAAAATAAAATAAAATAAAATAAAAATAAAAATAAAGCAAAATAAAAATAA
AATAAAATAAAATAAAATAAATAAAATAAAATAAAATAAAGCCAAAATAAAATAAAATAAAA
TAAAAAATAAAATAAAAAATAAAATAAATAAAATAAAATAAAATAAAAATAAAATAAAATAA

AATAAAAATAAATAAAATAAAAATAAATAAAATAAAAATAAAATAAATAAAAATAAAATAAA
ATAAAATAAAATAAAATAAAATAAGAAGAAGCCAAAGCACAACGACTCCGCATACCTGTAAT
CCCAGACTTTAGGAGGAGGAGGCCGAGGTGGGCGGATCACCTGAGGTCGGAGTTCGAGACCG
GCCTGGGAAACATGAAACTCCATCTCTACTAAAAATACAAAAATTAGCTGGGCATGGTGAGG
TGGGCGTAATCCCAGCCTACTCGGGAGGCTGAGGCAGAAGAGAATCACCAGACAGGAGGCTG
AGAGGTGCAGTGAGCCAAGATAAGCACCATTGCACTCCAGCCTGGGCAACAGGGGGTGAGAC
TCCATCTCTAAAATAAATAAAAAAATAAAATAAAATATAAGTGTTTGTTATCTGGCACGTGA
AGTAGCCTCAAATGTTAACTCTATGAAGGTTTATGGTTTTAAAATGTCAATTGATATCTTTT
GTCCGTTTTTATTGGGTTATATTATAAGTATGTATGTATTTTCCCCTTTGTTTTTAAATTGA
TAAATTCACATACCATAAGATTCATTATTCTTTACAGTGTACAATTCTGTACTTGTTAGTAT
ATTCTACAAGGTTGTGCAACCATCACCCAATTCAGAACATTTTTATCACCCCAAAAAGAAAC
CTCTGTATCTGTCATTAGTCACTTCCCCATTCCTGCCTCCTCCCAGTCTAGGCAGCCACTAA
TCTTTCTGTCTCTATGGAGTTGCCAATTCTGGACATTTCAAGTAAATGGAATCATATGATAT
AATAACTTTGTGTTGAGCTTCTTTCACTTAGCATACTTTCAAGTTCAACCATGCTGTACTTT
ATTCCTTTTTATAGCTGAATAATATTCCATTGTATGGATAGATCCCATTTTGTTTATCCATT
CATCAAATAAAATTTGTTTCACTATTTTATTATAGTGCTATATAATAGCACTATATTATATT
ACTGCTATTATAATACTGCTGGCGGACGTGAGCTGACTCATGCCTATAATCCTAGCACTTGG
AGGCCAAGGTGGGTGGATCCTCTGAGGTTGAGTTCGAGACCCAGCCTGGCCAACATGGCAAA
ACCCCATTTCTACTAAAAATACAAAAATTAGCCAGGTGTGATGGCAGGAGCCTGGAATCCCA
GCTACTTGGGAGGCTGAGACAGGAGAATTGAGACCCAGAGGTGGAGGTTGCAGTGAGCCAAG
ATTGCACCACTTCACTCAGCCTGGGCGAAAGACAAAACTGTCTCAAAAAAAGAATACTGCTA
TGGACATTTGTGTACAAATCTTCATATAGACATGTTTTCAATTCTCTTGGGTAGGTCCTAGG
AGCAGATTTGATGGATCATGGTAACTATGCTTAACTTTTAAGCAACTGTAGAAATATTTTCC
AAACATTACACTATTTTATATTCACATCAGCAATGTTTGAGATTCTAACTGCTCCACATCCT
TGGTTCAATTGCCCCACTTGTTGTGTCCATCTTTGTTATTCAGCCAACTTAGTGGGTGTGAA
ATGGTATCTCATTGCGTCTTGACTGGTATTTCCTAACCTTTTCATGTGCTTATACTAGTCTT
GATAGTATCCTTTCATGCATAAAAGTTTTTAATTTTGATGTTTATTTTTCTTTTGATTGCTT
GTTTTTGGTGTTATAGCCAAGAAAACCACTGCTAATCAAGGTCATAAGAACTTATGCTATAT
TTTCTTCTAAGGATTTTAGAATTTTAGCTTACATTAGGTCTTTGCCCATTTCAAGTTAATGT
TTGTGTATGATAATGAGGTAGGAGTCTAAGCTCATTCTTTACATCTGCTTCTCCAGGTGACC
CAGTGTCATTTGTCGAAAAACTATTCCTTCCCAGTGAACTATCCTGGCATCCTTGTTGAAAA
CTGACCAAAAATGTAAAGGTTTATTTCCTAGACTCTCCAATTTTATTCTAACAATCTGTAGA
TGTCTATCCTTATGCCAGTACCACAATGTCATTACTGTAACTGTAATAAGTTTTGAGGTAGG
AGACAGTGAGGTCCGTCAAACTGTTCTCTTTTTTCAAGACTGTTTTATATTCTGAGGTTCCT
TTCATTTCCATATGACTATCAGAAGTGGCTTGTAATTTCTGCAAGAACTGCCTAAGTTTTGA
TAAAGACTACACTAGATCACTTTTAAGTGGTATTGCCATCTTAGCAATATTAAGTGTTCTAA
TCTAATCCATGAACACTTGAGGATCTTTCATATGTCTTTTTAAATTTCTTCCAATGATGTTT
TGTTGTTTTTAGTGTTACAGTTTTAGTTGTACTTGTTGAATTATTAGTATGTATTTATTCCT
TTTATGCTACTGTAAATAAATTGTTAATTTTATTTTCAGATTCGATGACAGTGTACATAAAC
ATGACTTCGTGTATTGATCTTGTATCCTGCAAACTTAGTACAGAACTCATTTATCAGCTCTA
ATTACTTTCTGTAGATTCCTTACTATTTTTCTAAATATAAGATCATGTCAGCTACAAATAGA
GATAGTTTTATTTCTTCTTTCAATCTAAATGACTTTCATTTCCTTATGTTGCCTGATTTCAT
TGGTTATGACCTCCAGTACAATGTTGAACAGAAATGGTGAGAGCAGAATCCTCATCTTGTTT
TCTGATCTTAGGAAAAGCACTAAGTCTTTTGCCATTGCTACACTGTTAGCTATGTTTTTGTA
GATGCCTTTTTATCAGCTGCAAGAAATTTCCTTCTATTCCCAGTCTGCTGAGTGTTTTCTTT
TTAATCACAAAGAGGGCTGGAATTTGCAATCAAATCTTTTTTGTGTTTATTCAGATGATCAC
GCAGTTTTGTCCTTTTTCTATTAATATGGTATGTCGCATTGATTTTGTATGAAAGAACCCGA
CCTTGCCGTTCATAAGTAAATCCTACTTGCATGGCATATAATCCATTTCGCTGAGTTCAGTT
TGTTGAGTATTTTGTTCAGGACTGCAAAAAAATGTGGTTTTTTGTTTGTTCTGAGCGGAAGC
TAGAGTGCAGTGGCATAATCACAGCTCCTACAGCCTTAACTCCCAAGCTCAGGTGATTCTCC
ACTTCAGCTTCCCAAGTAGCTGGGACTACAGACAAGCACCACCACATGCAGCTAATTTGTTT

TCTGTATTTTTAATGTTGGAGATGCGGTGCACCATGTTGCCCAGGCTGGTGAACTCTGGCCT
CCAAGTGATCCACTAGCCTGGGCCTCCCAAAGTGCTGGGATTCAGGCGGGACTCACACAGGC
TGCAAAGTTTAAAACATGAAAAACACCACCATATATTGTACTATGTATCACTATGCTCATTC
AGGGAGTAGCATATACATATGTATAAAAACATAAGGAATTATATACAACAAATTGGGATAAT
GGTGACATCTTGGGAAAAATAAGAATACAACTGGAAAAGGTACAGAGGAACCCTAAGTATAC
TTGTAATATCTTATTTGTAACATAGCTGTACTTACTGTAGCTAAGTGTTATTATTTAAACCT
TTTTTTATATATCTGAAGCATTTATAATAATAATTTTTAAAACTTGGCTTGTATTTTTCCAT
AACCTCTTCTTTTTGAGACAAAGTCTCACTATGTCGTGAAAACCTTGGCCTCAAATTCCTGG
GCAAGAGATCCTTTCACTTCAGCCTCCCAAGTAGCTGGCACAATAGGCGTGTGCTACCATAC
CCAGCTTCTCCGTGCCCTCCACCTCACCCTACCCCTGCAACATTTAAAGAAAGGTTACAGAT
AAGAAACAAAGTGCTGGCTGGCTGTCTTATACACTCTATTGCAATTCAAGACTGAGAGAAGA
CCCAAATTACTGCTACACTGTCTGAAATGCCTTTTCACCCTTCAAGCTTCAAAAACCACAAA
GGTAACTGCTAGGCCGGGATTAACCATACACACTGATTTTTATTCCTTTACACAGGCATATT
ACACTATAATTAATCTATCTTTATATCTGCTTTCCTTACTAAACTATAAATTCCTTAATAAG
CCAGAATCAGTCTTTTTCATATTTGTAGTTCCAGCTCCTAACATGTCTGGTAAGCTTGAACA
TGTCCTGGTAAACATTACATACCACATTACATGAATAAATTTAGTAAATCAAGAAATAAAAT
GAAAATAGGCCTCAGTAATCAGAAATTTTTATTAAAAAGATTGCACGTAAGGCCTGGGGCAG
TCGGTTCACGCCTATAATCCTCAACATTACGGGAGGCTGGAGTGGAAACGAATCCCACTTGA
GAACCCAGGACTTCAGACCAGCCTGGGCAACATCTCTAAAAAAAAACATTACACAACAAATA
GCTGAGCACAGTGGCAATACAGCTGTAGTCCAGCCCCTACTGAGGAGAGGGTGGGAGAATCA
CCTGAGCCCTGGAGGTCGGGACTGCATGGCGAGCCAAAATCATGCCACTGCACTCCAGCCTG
GCAACAAGAGTAAGACTCTTAAAAAAAAAAAAAATTGACCCGGGTGCAACGGCTCACACCTGT
AATCCCAGCACTACAAAAATAGCCAGGCATGAGGTGGCCTGCGCCTGTAATCAGCTACTCAG
CAGGCTGAGGCACAAATCTGTTGAACCCAGGAGATGGAGGTTACAGTGAAGAGTTGAGATCG
TGCACTCCAGCCTGGGTGAGAATAGCTGTCTCCAAAAATATATATATATTGCATTAATACTA
TATTGCTGCAAAAAGCCAAAAATTCAAAGGTATAGTGTCAATCAAGATTAAAGTTTAGAGCA
ATTAAAAATAATGGTAAAGTCTAAAGATTTGTGTTCTAGTCCTGGCTCTACAGCTGACCAGC
TGTGTGATCTTATTTTTATTTTTACCTTTAAAGTGAGGACGTTATTTCTTGAAGTCCTAGTT
CTAAAATTCAGATTTGCTTTGAATTTTAAGCTTTGGACTTGGATAAAAAATATTTATATAAG
AATATTTGTCATTTTGACTGCCTAATCCTTTCTATTTGGAAGAACCCAAATAGGTGAATGCA
GCCCCTCTCCGCCCACAAGCAGTTAAAGCACGGCTCTAATTTAAGTGAGGTTAGAGACTTCT
AGGGGCTTTGCATCTTGAAAGATGAGCACAGGATCTTGAGGGAGCTGGGAGGAGTCCAGGAG
TACAACCAATCTGGGGAAAGAATGCCAGCACTGCTCTAAGTAGATTATTCCTGCTGCAGGTC
TTGGTCAGCAACGCTGTCAATACTGGTGACCCTAATACCGTTTTGATTAATTCCTTTTTGAA
ATTAACCAAGACAGTTTCTTTAATTTGCAGCCAAGAACCCTGACCAGCTGCAAAACCATAGA
AACAGAGAAGGAGCCTTAGGATAGGCATTTAGATGCAGAAGAGAGAGCAAGCAAGCCATAGC
TAGATTTGCTCGAAGACAATCGGAAGCATATAGGGCATCGATTACAGCATTTTTGAGGTCTA
TAAAGCATCCTTCAACAATAACTAAGCACAACTCTTGAGAGTGTCATTCTTTAAAAAATTTT
TCTTCATTGCCCAAGCTGGCCTCAAATTCTGGGCTCAAGTGATCCTCCAGCCTCCCAAGTAG
GCACATGCCTGCAACACCTGGCCGAATTATCATTCTTGCCACACTTAATAAAAGGGAGCATG
TTTCCACTGGTGGAATGTCGTACTAAACATCGAGGCTCATAAAATAATTACATGGTTAATAA
GAAGTTTTTAAAAATTATTAACTATAGGCAACATTTTTCATGACCTTCTAAGAATCCAAGGT
GGTTCAGAGCATCTAGCAATACGTAACCT
