# Supplementary material for: Intronic ATTTC repeat expansions in STARD7 in familial adult myoclonic epilepsy linked to chromosome 2
Source: Nat Commun. 2019 Oct 29;10:4920. doi: 10.1038/s41467-019-12671-y (PMC6820779; doi:10.1038/s41467-019-12671-y)
Supplement: Supplementary file 4 — Reporting Summary [file 41467_2019_12671_MOESM4_ESM.pdf]

nature research

Corresponding author(s): Jozef Gecz

Last updated by author(s): Sep 5, 2019

Reporting Summary

Nature Research wishes to improve the reproducibility of the work that we publish. This form provides structure for consistency and transparency in reporting. For further information on Nature Research policies, see Authors & Referees and the Editorial Policy Checklist.

Statistics

For all statistical analyses, confirm that the following items are present in the figure legend, table legend, main text, or Methods section.

n/a	Confirmed	
☐	☒	The exact sample size (*n*) for each experimental group/condition, given as a discrete number and unit of measurement
☐	☒	A statement on whether measurements were taken from distinct samples or whether the same sample was measured repeatedly
☐	☒	The statistical test(s) used AND whether they are one- or two-sided<br>*Only common tests should be described solely by name; describe more complex techniques in the Methods section.*
☐	☒	A description of all covariates tested
☐	☒	A description of any assumptions or corrections, such as tests of normality and adjustment for multiple comparisons
☐	☒	A full description of the statistical parameters including central tendency (e.g. means) or other basic estimates (e.g. regression coefficient) AND variation (e.g. standard deviation) or associated estimates of uncertainty (e.g. confidence intervals)
☐	☒	For null hypothesis testing, the test statistic (e.g. *F*, *t*, *r*) with confidence intervals, effect sizes, degrees of freedom and *P* value noted<br>*Give P values as exact values whenever suitable.*
☐	☒	For Bayesian analysis, information on the choice of priors and Markov chain Monte Carlo settings
☒	☐	For hierarchical and complex designs, identification of the appropriate level for tests and full reporting of outcomes
☒	☐	Estimates of effect sizes (e.g. Cohen's *d*, Pearson's *r*), indicating how they were calculated

Our web collection on statistics for biologists contains articles on many of the points above.

Software and code

Policy information about availability of computer code

Data collection	No unpublished software was used for data collection

Data analysis	The following programs were used in this analysis:<br>BWA-MEM v.0.7.15<br>htslib v1.9<br>Genome Analysis Toolkit v3.8 and v4.0<br>Integrative Genomics Viewer (IGV) v2.4.5<br>VarSeq v1.4.7<br>Lumpy v0.2.13<br>Manta v1.2.2<br>SMRT Link software v5.1.0<br>NGM-LR v0.2.6<br>Sniffles v1.0.7<br>Canu v1.7<br>Circular consensus calling software v3.2.1<br>MinKNOW Core 1.11.5<br>Albacore v2.1<br>ExpansionHunter v2.5.5<br>exSTRa v0.88.3 with Bio-STR-exSTRa v1.0.1<br>HISAT2 v2.1<br>StringTie v1.3.3<br>R v3.4.3<br>edgeR

For manuscripts utilizing custom algorithms or software that are central to the research but not yet described in published literature, software must be made available to editors/reviewers. We strongly encourage code deposition in a community repository (e.g. GitHub). See the Nature Research guidelines for submitting code & software for further information.

Data

Policy information about availability of data

All manuscripts must include a data availability statement. This statement should provide the following information, where applicable:
- Accession codes, unique identifiers, or web links for publicly available datasets
- A list of figures that have associated raw data
- A description of any restrictions on data availability

Source data for figures are provided in Supplementary and Source Data files with this manuscript. Whole genome sequencing data and RNA-seq data are available from the corresponding author on request, subject to human research ethics approval and patient consent.

Field-specific reporting

Please select the one below that is the best fit for your research. If you are not sure, read the appropriate sections before making your selection.

☒ Life sciences ☐ Behavioural & social sciences ☐ Ecological, evolutionary & environmental sciences

For a reference copy of the document with all sections, see nature.com/documents/nr-reporting-summary-flat.pdf

Life sciences study design

All studies must disclose on these points even when the disclosure is negative.

Sample size	Sample consisted of 272 individuals comprising 29 controls, 158 affected individuals, 26 individuals examined but without a FAME diagnosis, 51 individuals with a FAME like diagnosis and 8 individuals with an uncertain diagnosis.
Data exclusions	DNA samples that were not able to be amplified by repeat primed PCR due to DNA quality were excluded
Replication	Tests results were confirmed with duplicate (technical) replication.
Randomization	All samples were tested, randomization was not necessary as there was no treatment applied.
Blinding	Where possible tests for the repeat were carried out with the researcher blinded to the diagnosis however for some samples where we have researched for many years the identity was known and difficult to blind. Given the clear difference between positive and negative test results it is reasonable to assume that there is no bias in the interpretation. All test data are shown in Supplementary Table S2.

Reporting for specific materials, systems and methods

We require information from authors about some types of materials, experimental systems and methods used in many studies. Here, indicate whether each material, system or method listed is relevant to your study. If you are not sure if a list item applies to your research, read the appropriate section before selecting a response.

Materials & experimental systems

n/a	Involved in the study	
☒	☐	Antibodies
☒	☐	Eukaryotic cell lines
☒	☐	Palaeontology
☒	☐	Animals and other organisms
☐	☒	Human research participants
☒	☐	Clinical data

Methods

n/a	Involved in the study	
☒	☐	ChIP-seq
☒	☐	Flow cytometry
☒	☐	MRI-based neuroimaging

Human research participants

Policy information about studies involving human research participants

Population characteristics	Individuals tested in this study were from Australia (European), New Zealand (European), Italy, France, Spain, South Africa (Dutch heritage), Iraq (Jewish), Israel (Sephardic) and Syria.
Recruitment	Individuals were recruited with a prior diagnosis of Familial Adult Myoclonic Epilepsy.
Ethics oversight	This study was approved by the Human Research Ethics Committees of the University of Melbourne and the University of Adelaide. Written, informed consent was obtained from all participants in the study.

Note that full information on the approval of the study protocol must also be provided in the manuscript.
