# Supplementary material for: Intronic ATTTC repeat expansions in STARD7 in familial adult myoclonic epilepsy linked to chromosome 2
Source: Nat Commun. 2019 Oct 29;10:4920. doi: 10.1038/s41467-019-12671-y (PMC6820779; doi:10.1038/s41467-019-12671-y)
Supplement: Supplementary file 5 — Description of Additional Supplementary Files [file 41467_2019_12671_MOESM5_ESM.pdf]

Title: Supplementary Data 1
Description: Contains Supplementary Data 1: Combined summary of RP-PCR results from all families.
Title: Supplementary Data 2
Description: Contains all long read sequences that span the ATTTC repeats in fasta format.
